# Selective Delivery of Anticancer Natural G‑Quadruplex Ligands by the AT11 Aptamer for Gastric Cancer Treatment

**DOI:** 10.1021/acs.jmedchem.5c02521

**Published:** 2025-12-15

**Authors:** Chiara Platella, Marko Trajkovski, Mariarita Brancaccio, Rosita Di Palma, Andrea Calcaterra, Mattia Mori, Geppino Falco, Janez Plavec, Daniela Montesarchio

**Affiliations:** † Department of Chemical Sciences, 9307 University of Naples Federico II, via Cintia 21, Naples 80126, Italy; ‡ Slovenian NMR Centre, 68913 National Institute of Chemistry, Hajdrihova 19, Ljubljana 1000, Slovenia; § Department of Biology, 9307 University of Naples Federico II, via Cintia 21, Naples 80126, Italy; ∥ Department of Chemistry and Technology of Drugs, Sapienza University of Rome, Piazzale Aldo Moro 5, Rome 00185, Italy; ⊥ Department of Biotechnology, Chemistry and Pharmacy, 9313 University of Siena, via Aldo Moro 2, Siena 53100, Italy; # Faculty of Chemistry and Chemical Technology, University of Ljubljana, Večna Pot 113, Ljubljana 1000, Slovenia; ∇ EN-FIST Center of Excellence, Trg Osvobodilne Fronte 13, Ljubljana 1000, Slovenia

## Abstract

Searching for G-quadruplex-selective ligands as anticancer agents, we recently identified the natural compounds bulbocapnine, chelidonine, dicentrine, ibogaine, and rotenone as novel interactors of G-quadruplexes. Herein, to investigate their ability to interact with a specific carrier for selective delivery to cancer cells, the dimeric G-quadruplex-forming aptamer AT11 was used as a model. NMR spectroscopy, molecular modeling, circular dichroism, and fluorescence spectroscopy allowed the preferential interaction to be proven with the 3′-end G-quartet for bulbocapnine, chelidonine, dicentrine, and ibogaine, whereas with the 5′-end G-quartet region for rotenone. The anticancer activity of the AT11/natural compounds complexes was evaluated on gastric cancer cells using the free aptamer and free natural compounds as controls. Notably, all complexes caused a significant decrease in cancer cell viability, also producing synergistic effects. Remarkably, no relevant effects were detected on noncancerous cells, denoting the importance of delivering the natural compounds by AT11 G-quadruplex to obtain selective antiproliferative effects on cancer vs. normal cells.

## Graphical abstract

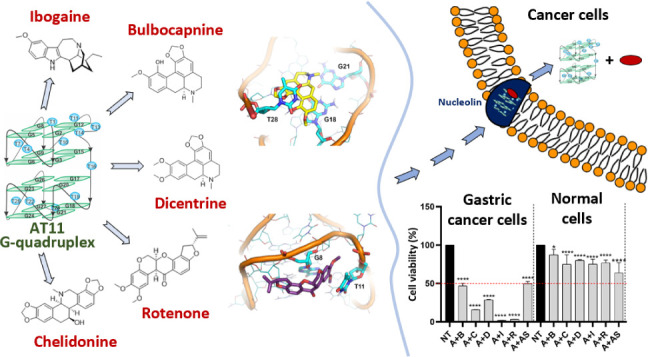

## Introduction

G-quadruplexes are 3D structures formed by folding of guanine-rich oligonucleotides, whose location is nonrandom in human as well as in viral and bacterial genomes.^1–3^ Indeed, G-quadruplexes can act as modulators of functional processes involved in the cell cycle,^4,5^ being mainly found in replication origins, (onco)­gene promoters, and telomeres.^6,7^

The high interest in G-quadruplexes is not limited to natural, genomic G-quadruplex structures as targets for potential anticancer drugs, but also extended to synthetic G-quadruplexes, which can find relevant applications in therapeutic, diagnostic, and biotechnological applications. Over the past two decades, numerous G-quadruplex-forming oligonucleotides have been discovered to be active as aptamers—i.e., nucleic acid-based molecules forming peculiar structures capable of specifically binding cellular targets—for cancer-related proteins.^8,9^

Among them, AS1411 has reached Phase II clinical trials, showing promising activity against metastatic renal cell carcinoma (ClinicalTrials.gov Identifier: NCT00740441) and acute myeloid leukemia (ClinicalTrials.gov Identifier: NCT00512083) with minimal toxicity.^7,10^ AS1411 is a synthetic G-rich 26-mer oligonucleotide, first discovered by Bates and coworkers, able to specifically recognize nucleolin,^11^ a multifunctional protein playing essential roles in cell survival, growth, and proliferation, mainly located in the normal cell nucleus. However, in cancer cells, this protein is also present in the cytoplasm and on the cell surface. This feature confers a tumor-selective cytotoxic behavior to AS1411 which preferentially targets the external domain of surface nucleolin of cancer cells.^7,10,12^ Interestingly, the applications of AS1411 are not only restricted to its use as a potential anticancer drug *per se*, but it has also been studied as a carrier for delivering other drugs into cancer cells.^12,13^

Notwithstanding relevant efforts, the effective bioactive conformation of AS1411 is still unknown essentially because of its high polymorphism, due to the coexistence of multiple different G-quadruplex structures.^14^ Aiming at developing analogues of AS1411 maintaining similar bioactivity profiles but with lower conformational heterogeneity, the 28-mer oligonucleotide d­(TGG-TGG-TGG-TTG-TTG-TGG-TGG-TGG-TGG-T), named AT11, derived from AS1411 and exhibiting similar antiproliferative activity,^11,15^ was identified as a very promising drug candidate. AT11 differs from AS1411 since thymidines were added at both 5′- and 3′-ends, as well as in position 10 of the AS1411 parent sequence, and these modifications allow AT11 forming a unique major species, i.e., a dimeric parallel G-quadruplex, whose structure was resolved by NMR.^15^

AT11 was recently proven to act as an effective active targeting agent in drug liposomal formulations for novel anticancer approaches.^16–19^ The interest in AT11 and its analogues is also further motivated by recent studies proving their ability to act as supramolecular carriers for the selective delivery to cancer cells of small synthetic molecules with *in vitro* anticancer activity, e.g., acridine-based ligands^20^ or Zn­(II)-phthalocyanine derivatives,^21^ able to selectively bind and stabilize G-quadruplex structures.

In the context of research focused on the identification of G-quadruplex-selective ligands as anticancer agents,^22–26^ we have recently evaluated libraries of natural compounds and discovered novel interactors of G-quadruplex structures.^27–32^

In detail, the natural alkaloids bulbocapnine, chelidonine, dicentrine, ibogaine, and the natural isoflavone rotenone (Figure 1) proved to strongly bind monomeric G-quadruplex models by both affinity chromatography^33^ and biophysical studies.^29,31^ Additionally, chelidonine, dicentrine, and rotenone showed anticancer activity in the low micromolar range, which correlated well with their ability to target telomeric and oncogenic G-quadruplex structures in cancer cells.^29,31^

**Figure 1:**
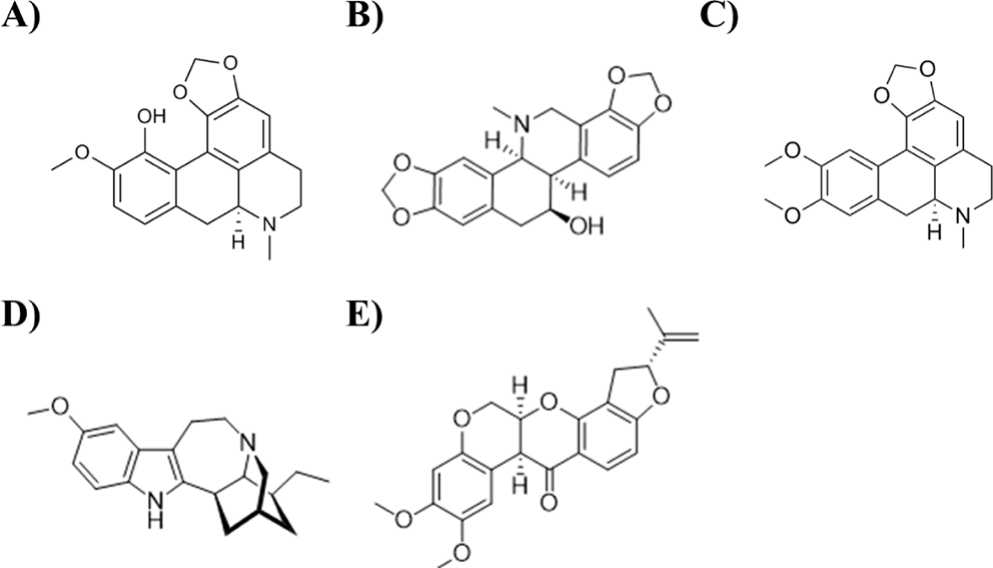
Chemical structures of (A) bulbocapnine, (B) chelidonine, (C) dicentrine, (D) ibogaine, and (E) rotenone.

Herein, AT11 was selected as the model of a G-quadruplex-based drug delivery carrier to favor the selective uptake into cancer cells of bulbocapnine, chelidonine, dicentrine, ibogaine, and rotenone, transported in the form of complexes by this nucleolin-targeting G-quadruplex-forming aptamer. The combined use of NMR spectroscopy, molecular modeling, circular dichroism (CD), and fluorescence spectroscopy allowed us to obtain information on the binding mode of the above natural compounds to the AT11 G-quadruplex. Moreover, fluorescence spectroscopy was also exploited to evaluate the natural compounds release from the AT11 G-quadruplex. Finally, the anticancer activity of the complexes formed between the AT11 G-quadruplex and each natural compound was evaluated using the free natural compounds and the free aptamer as controls. Taking into account that gastric cancer (GC) is still highly challenging to treat and the current therapies are associated with non-negligible adverse effects,^34–37^ new effective and low-toxicity anticancer agents need to be discovered. Thus, GC was used here as a proof-of-concept disease model to evaluate the importance of targeted therapeutic treatments based on the combination of antiproliferative G-quadruplex ligands of natural origin and G-quadruplex-based carriers.

## Results and Discussion

### NMR Characterization of Interactions between the Natural Compounds and AT11 G-Quadruplex

To analyze in detail the interaction of the selected natural compounds with the G-quadruplex-forming AT11 aptamer, an in-depth^1^H NMR investigation of the complexes obtained at different ligand/DNA ratios was carried out. As a preliminary study, analysis of AT11 alone was performed in an aqueous solution at 70 mM KCl and 20 mM potassium phosphate buffer (pH 7.0), corresponding to the conditions previously used for the structural characterization of AT11.^15^ In the ^1^H NMR spectrum of AT11, 16 imino proton signals were observed, consistent with the formation of a unimolecular G-quadruplex structure containing four G-quartets (Figure S1, bottom). In detail, AT11 folds into a G-quadruplex with two parallel-stranded subunits stacked one on top of the other, forming a peculiar G-quadruplex-G-quadruplex interface and connected through the T16 residue (Figure 2). The first subunit consists of the two G-quartets G2:G5:G8:G12 and G3:G6:G9:G15, while the second subunit consists of the two G-quartets G20:G23:G26:G17 and G21:G24:G27:G18. The 5′- and 3′-flanking ends comprise T1 and T28, respectively, while the propeller-type loops involve T4, T7, T10-T11, T19, T22, and T25 residues. Finally, a two-residue bulge, comprising T13-T14, is formed between G12 and G15 of the first subunit (Figure 2).^15^

**Figure 2:**
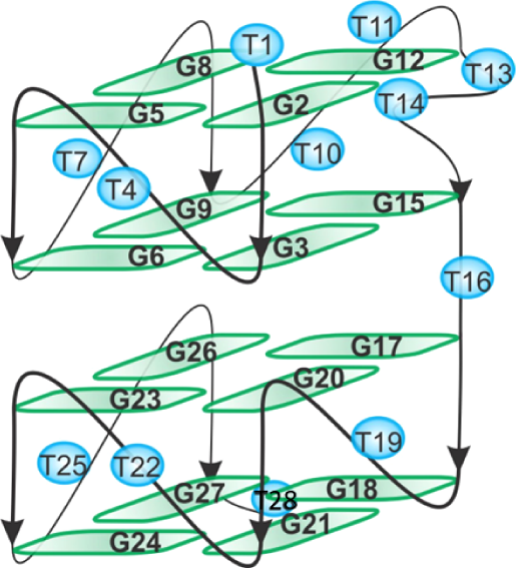
Schematic representation of the G-quadruplex structure formed by AT11. Guanine and thymine residues are depicted as green rectangles and blue spheres, respectively.

Since the below-discussed titrations were carried out using the natural compounds dissolved in DMSO, with the only exception of bulbocapnine, the free AT11 G-quadruplex was also analyzed in 70 mM KCl, 20 mM potassium phosphate buffer (pH 7.0), to which up to 3% of DMSO was added, corresponding to the highest amount of DMSO added during titration experiments with the natural compounds (Figures S1–S3). The obtained ^1^H NMR chemical shifts for the free AT11 G-quadruplex are reported in Table S1 and are in full agreement with the ones indicated in the previous study describing AT11 structure under the same experimental conditions.^15^ In parallel, the ^1^H NMR chemical shifts for the AT11 G-quadruplex in the DMSO-containing buffers have been reported in Table S1 as well. Interestingly, ^1^H NMR chemical shift differences for the AT11 G-quadruplex in the presence of DMSO compared to those in the DMSO-free buffer are within the estimated experimental error, i.e., 0.05 ppm, for all residues, including the thymines of the flexible loops that are oriented toward the bulk solvent.

The interactions between AT11 G-quadruplex and the individual natural compounds were assessed by ^1^H NMR-monitored titrations of the DNA solution with the ligands, added up to a 1:6 DNA/ligand ratio (Figures S4–S18). However, due to the lower solubility of rotenone in the explored NMR conditions compared to the other ligands, the titration above a 1:3 DNA/ligand ratio was precluded for this compound.

Moreover, to get a deeper insight into the interactions between the AT11 G-quadruplex and the natural compounds investigated here, NOESY experiments were performed on the samples at different DNA/ligand ratios, thus allowing the assignment of the ^1^H NMR chemical shifts of the AT11 G-quadruplex in the presence of different molar equivalents of each compound (Figures S4–S18), which are reported in Tables S2–S4.

Apart from rotenone, addition of each of the other ligands to the DNA in aqueous solution was coupled with perturbations in the ^1^H NMR signals attributed to the AT11 G-quadruplex. Interestingly, most of the imino signals shifted upfield, although differently for each ligand, hence suggesting formation of complexes with different features.

More in detail, in the case of the 1:1 AT11/bulbocapnine system, the most affected imino protons were G2, G5, G15, G18, G21, and G24, while the highest changes in ^1^H NMR chemical shifts for the aromatic and methyl protons were observed for T28 (Figures 3A, S4–S6 and S19A). Interestingly, the ligand-induced deshielding of aromatic and methyl ^1^H NMR signals of T28 was indicative of a weaker stacking of T28 onto the nearby G-quartet compared to T28 in the free G-quadruplex, as a consequence of ligand binding. At a 1:6 AT11/bulbocapnine ratio, significant ^1^H NMR chemical shift differences were observed for all imino protons except for G8, G9, and G12 (Figures 3A and S4) indicating that their chemical environment was not altered after addition of the ligand. For the aromatic and methyl protons, upfield shifts were observed for the G-quadruplex-G-quadruplex interface residues G3, G6, G9, T14, G17, G20, G23, and G26, as well as for G21 in the 3′-end G-quartet, indicating that the ligands promoted a stiffer packing of the central part of the AT11 G-quadruplex (Figures S5–S6 and S19A). On the other hand, the ligand interactions were coupled with a downfield shift for aromatic and methyl protons of T1, T11, G27, and T28 (Figures S5–S6 and S19A), suggesting that these residues, initially capping the 5′- and 3′-end G-quartets, were destacked from the outer G-quartets. These results are consistent with stacking of bulbocapnine onto both the 5′- and 3′-end G-quartets at a 1:6 DNA/ligand ratio, thus perturbing the original stacking interactions of flanking segments on the external G-quartets. Notably, the primary binding sites of bulbocapnine on the AT11 G-quadruplex are the two outer G-quartets, with a preference for the 3′-end G-quartet at a 1:1 DNA/ligand ratio, whereas the grooves can also be involved in the binding of bulbocapnine when this compound is in large excess compared to the G-quadruplex.

**Figure 3:**
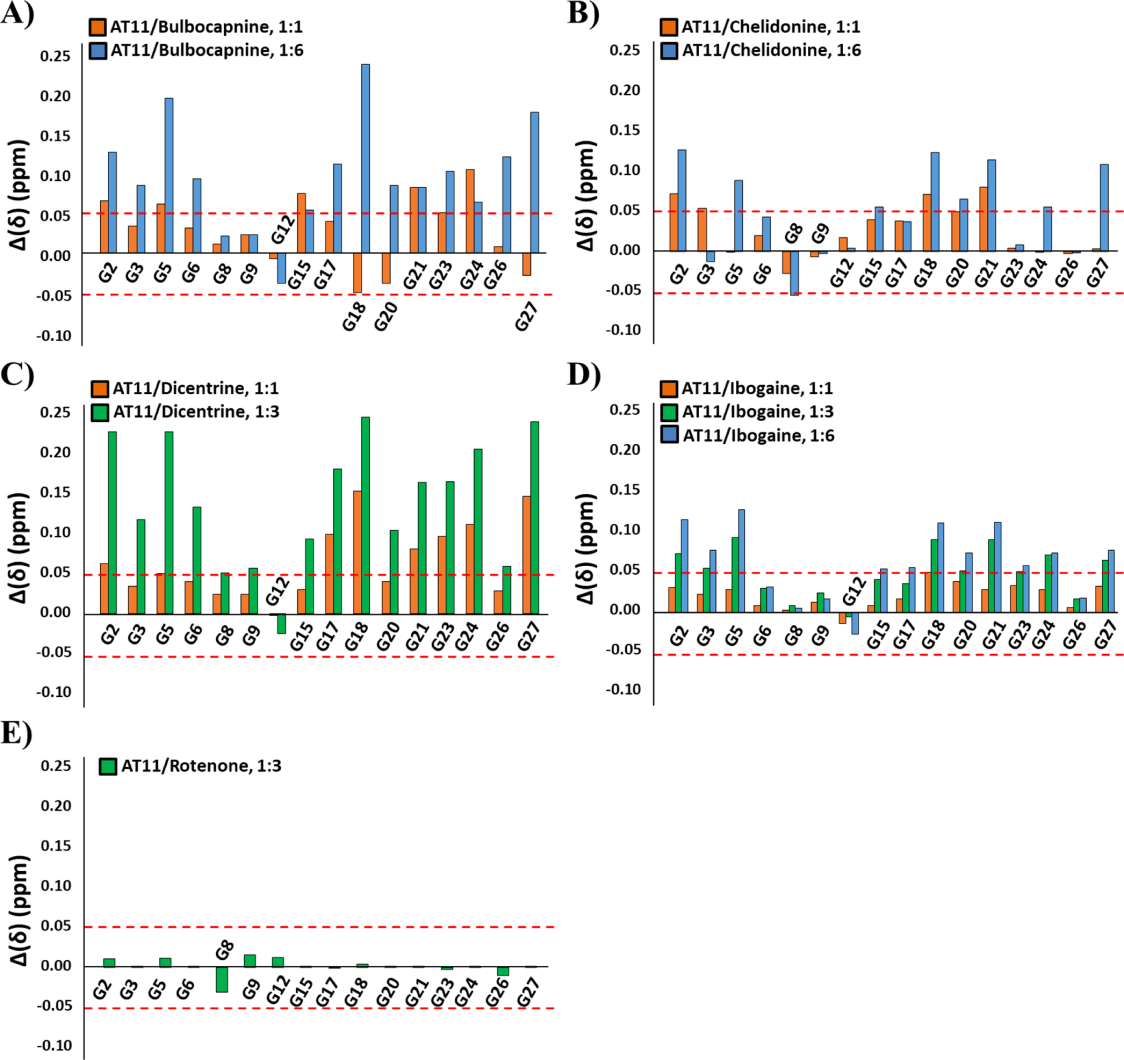
^1^H NMR chemical shift differences for imino protons of the AT11 G-quadruplex in the presence of 1 (orange bars), 3 (green bars), or 6 (blue bars) molar equivalents of (A) bulbocapnine, (B) chelidonine, (C) dicentrine, (D) ibogaine, and (E) rotenone with respect to the free AT11 G-quadruplex. The errors associated with chemical shift differences are within ± 0.05 ppm. Threshold lines were drawn as dashed red lines.

As far as the 1:1 AT11/chelidonine system is concerned, the most perturbed ^1^H NMR chemical shifts were observed for the imino signals of G2, G3, G18, G20, and G21, and for the aromatic and methyl protons of T11 and T28 (Figures 3B, S7–S9 and S19B). Noteworthy, the downfield shift of ^1^H NMR resonances for T11 and T28 is consistent with their destacking from the nearby G-quartets upon chelidonine binding. At a 1:6 AT11/chelidonine ratio, significant chemical shift differences were observed for imino protons of G2, G5, G8, G15, G18, G20, G21, G24, and G27 (Figures 3B and S7). A ligand-induced deshielding was observed only in the case of G8, indicating that this guanine was less stacked upon ligand binding. For the aromatic and methyl protons, upfield shifts were observed for G5, G6, G9, G20, and G26, and downfield shifts were observed for T1, T11, G27, and T28, indicating that these residues were, respectively, more and less stacked in the chelidonine-bound AT11 compared to the free AT11 G-quadruplex (Figures S8–S9 and S19B). These findings are consistent with chelidonine binding at both the 5′- and 3′-end G-quartets, accompanied by a rearrangement of the flanking end residues on the outer G-quartets. A secondary binding site at the groove in proximity to G9 and G26 cannot be excluded when the ligand is in large excess compared to the G-quadruplex. Notably, the binding of one chelidonine molecule at the 5′-end G-quartet was also supported by several AT11/chelidonine intermolecular NOE cross-peaks (Table S5). Interestingly, from inspection of the observed NOE cross-peaks, it seems that chelidonine can adopt different poses within the binding site at the 5′-end of the AT11 G-quadruplex, considering that protons such as a’,a’’ and m’,m’’ on opposite sides of the molecule appeared close to the same G-quadruplex residues.

In the titration of AT11 with dicentrine, the most perturbed imino ^1^H NMR signals at a 1:1 AT11/dicentrine ratio were G17, G18, G21, G23, G24, and G27, while the highest changes in ^1^H NMR chemical shifts for the aromatic and methyl protons were observed for G18 and T28 (Figures 3C, S10–S12 and S19C). Particularly, the results indicated ligand-induced deshielding for T28, suggesting weaker stacking of T28 onto the 3′-end G-quartet when the ligand was bound. At a 1:3 AT11/dicentrine ratio, significant ^1^H NMR chemical shift differences were observed for all imino protons (Figures 3C and S10), except for G12, which is one of the residues most hidden from the solvent of the AT11 G-quadruplex due to stacking of T1, T11, and T14 on the 5′-end G-quartet. For the aromatic and methyl protons, dicentrine-AT11 interactions were coupled with an upfield shift of the ^1^H NMR signals for G2, G3, G5, G6, G9, G15, G17, G18, G20, G21, and G23 and downfield shifts for T1, T11, G27, and T28 consistent with the higher or lower stacking of these residues, respectively, compared to the free AT11 G-quadruplex (Figures S11–S12 and S19C). On the other hand, NMR spectral analysis of the sample at a 1:6 AT11/dicentrine ratio indicated the coexistence of different complexes. In this regard, a detailed inspection of this sample was precluded by a severe signal overlapping. Altogether, these data demonstrated that dicentrine preferentially binds the 3′-end G-quartet at a 1:1 AT11/dicentrine ratio, resulting in the rearrangement of the T28 flanking end residue. Additional binding sites of dicentrine on the AT11 G-quadruplex, such as the 5′-end G-quartet and the grooves, can also be present when the ligand is in excess compared to the G-quadruplex. Remarkably, the position of dicentrine at the 3′-end G-quartet at a 1:1 AT11/dicentrine ratio, as well as the additional binding sites at a 1:3 AT11/dicentrine ratio, was also supported by several AT11/dicentrine intermolecular NOE cross-peaks (Table S5). Particularly, at a 1:1 AT11/dicentrine ratio, protons i and j of the ligand appeared close to G18, G21, G24, and G27 forming the 3′-end G-quartet, thus suggesting a dynamic behavior of dicentrine within the binding site at the 3′-end of the AT11 G-quadruplex.

Upon titration of AT11 G-quadruplex with ibogaine, the largest changes in imino, aromatic, and methyl ^1^H NMR chemical shifts at a 1:1 AT11/ibogaine ratio were those of G18 and T28 (Figures 3D, S13–S15 and S19D). Interestingly, ligand-induced deshielding was observed for T28, indicating a weaker stacking of T28 onto the nearby G-quartet in the ibogaine-bound AT11 compared to the free AT11 G-quadruplex. At 1:3 and 1:6 AT11/ibogaine ratios, significant ^1^H NMR chemical shift differences were observed for imino protons of G2, G3, G5, G18, G20, G21, G23, G24, and G27 (Figures 3D and S13). For the aromatic and methyl protons, upfield shifts were observed for G3, G5, G6, T14, G20, G23, and G26, while only T28 showed a downfield shift (Figures 3D, S14–S15 and S19D). These results suggested that ibogaine preferentially binds to the 3′-end G-quartet at a 1:1 AT11/ibogaine ratio, accompanied by the rearrangement of the T28 flanking end residue. However, secondary binding sites of ibogaine on the AT11 G-quadruplex, such as the grooves, can also be present when the ligand is in large excess compared to the G-quadruplex. Notably, the position of ibogaine at the 3′-end G-quartet at a 1:1 AT11/ibogaine ratio, as well as the additional binding sites at 1:3 and 1:6 AT11/ibogaine ratios, was also supported by several AT11/ibogaine intermolecular NOE cross-peaks (Table S5). Particularly, at a 1:1 AT11/ibogaine ratio, protons a, m, and o of the ligand were close to G21 and/or G27, thus suggesting dynamic equilibrium with ibogaine exhibiting different poses within the binding site at the 3′-end G-quartet of the AT11 G-quadruplex.

Finally, in the case of the AT11 G-quadruplex titration with rotenone, DNA-ligand interactions were coupled with only minor ^1^H NMR chemical shift perturbations, i.e., within the range of experimental error. The most affected imino proton was G8, while the highest changes in ^1^H NMR chemical shifts for the aromatic and methyl protons were observed for T11 at a 1:3 AT11/rotenone ratio (Figures 3E, S16–S18 and S19E).

### Molecular Modeling Studies of the Interactions between the Natural Compounds and AT11 G-Quadruplex

Based on the NMR data discussed above, the interaction between the natural compounds and the AT11 G-quadruplex was further investigated by molecular modeling, exploiting the available three-dimensional structure of the AT11 G-quadruplex.^15^ Specifically, molecular docking simulations were carried out with AutoDock,^38^ while molecular dynamics (MD) simulations were run with AMBER.^39^ MD simulations gave access to the analysis of the intrinsic flexibility of nucleotides not included in G-quartets and the global conformational landscape of G-quadruplexes, thus leading to explore ligand poses that might not be accessible by molecular docking wherein the G-quadruplex is treated as a rigid body.^29,31^

For all ligands, the 1:1 binding ratio to AT11 was investigated *in silico*. Docking-based binding poses generated by AutoDock were further relaxed by unrestrained MD simulations in explicit solvent, which were run for 500 ns on each system (up to two replicas for each AT11/ligand complex). The most representative MD frame extracted by cluster analysis, i.e., the centroid frame of the cluster with the highest frame population, was then selected for structural discussion (Figure 4).

**Figure 4:**
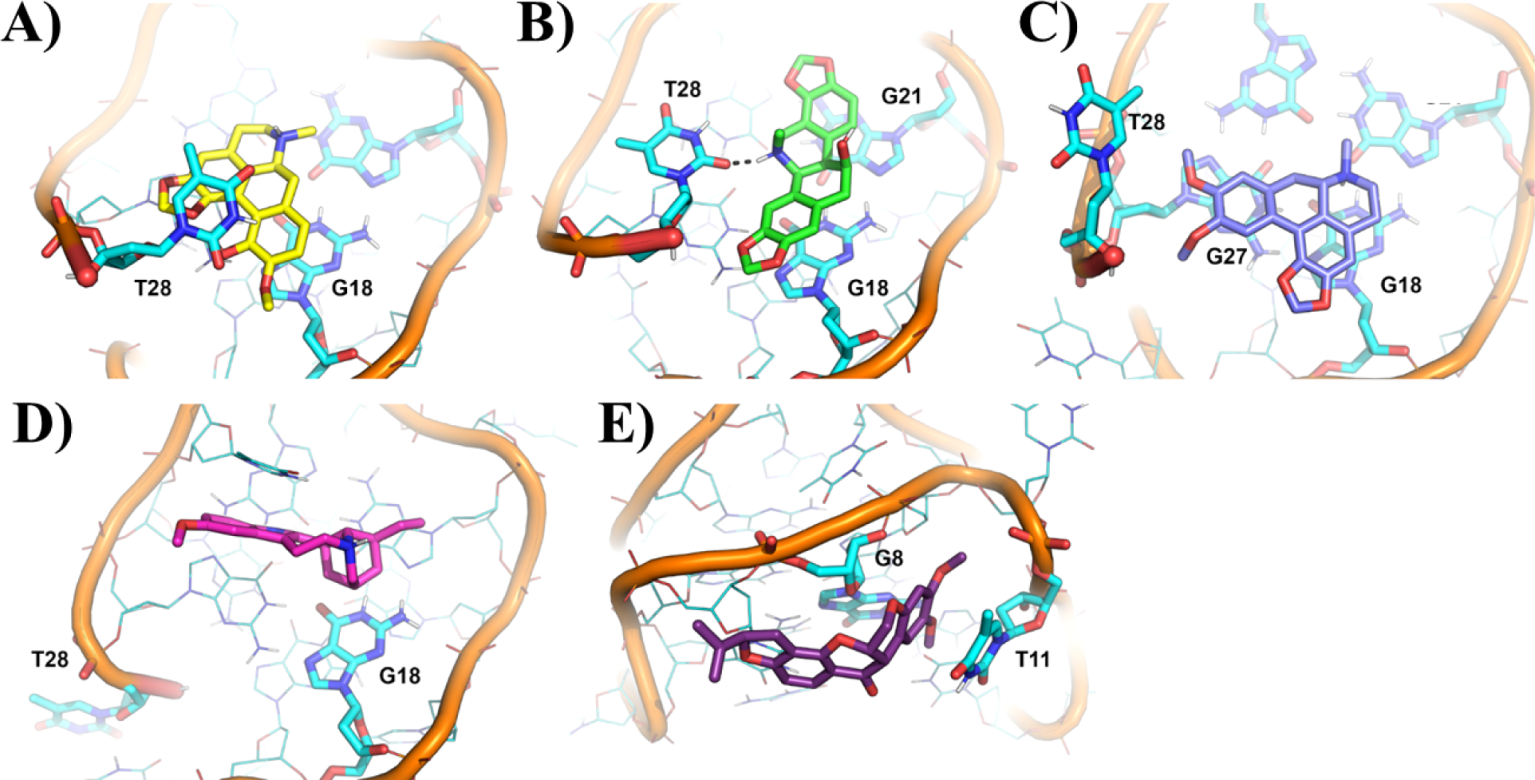
Binding mode of (A) bulbocapnine, (B) chelidonine, (C) dicentrine, (D) ibogaine, and (E) rotenone to the AT11 G-quadruplex as obtained by MD simulations. The most representative frame extracted from the MD trajectories is shown. The natural compounds and the AT11 G-quadruplex are shown as sticks and ribbon, respectively. Bulbocapnine, chelidonine, dicentrine, ibogaine, rotenone, and the AT11 G-quadruplex are colored in yellow, green, indigo, magenta, purple, and cyan, respectively. Polar interactions are shown by black dashed lines. Nucleotides involved in the interactions with the natural compounds are labeled and shown as sticks.

Bulbocapnine showed a remarkable preference for the 3′-end G-quartet, in agreement with NMR data for the 1:1 AT11/ligand complex. In detail, bulbocapnine was sandwiched between G18 and T28, thus explaining the weaker stacking of T28 onto the nearby G-quartet in the presence of the ligand compared to that of the free G-quadruplex, as observed by NMR (Figure 4A).

As far as chelidonine is concerned, it also showed a preference for binding to the 3′-end G-quartet. In addition to stacking interactions with G18 as in the case of bulbocapnine, the extended structure of chelidonine allowed the π-π stacking interaction with G21, in accordance with the NMR data. Interestingly, T28 moved from its initial position where it was stacked onto the nearby G-quartet, which can explain the weaker stacking interactions in the presence of the ligand compared to the free G-quadruplex as observed by NMR. Moreover, the O2 of T28 formed a hydrogen bond with the protonated amino group of chelidonine (Figure 4B).

Dicentrine showed a binding mode to AT11 G-quadruplex similar to bulbocapnine, as expected considering that the two ligands are structural analogues. As depicted in Figure 4C, the planar aromatic moiety of dicentrine fitted well on top of the 3′-end G-quartet forming stacking interactions with G18 and G27. The steric hindrance of the methoxy groups of dicentrine pushed T28 out of the plane, thus justifying the weaker stacking of T28 onto the nearby G-quartet in the presence of the ligand compared to the free G-quadruplex, in agreement with NMR chemical shift and NOE cross-peaks analysis.

In the case of ibogaine (Figure 4D), a preferential binding to the 3′-end G-quartet was found, in agreement with ^1^H NMR chemical shift perturbation analysis and the observed NOE cross-peaks, with stacking interactions formed between the ligand and G18. However, a lower overlap of ibogaine on this terminal G-quartet was observed compared to bulbocapnine, chelidonine, and dicentrine, most likely as a consequence of the reduced flatness of ibogaine compared to the other ligands. Moreover, T28 was flipped out from the G-quartet in agreement with the rearrangement of this flanking end residue, as observed by NMR.

Finally, rotenone showed a completely different binding mode compared to the other investigated compounds, mainly due to its V-like shape, which cannot be well accommodated on the flat surface of the G-quartets. Indeed, it was found to be located in proximity to the 5′-end G-quartet in correspondence with G8 and T11, in line with NMR data (Figure 4E).

Altogether, MD results confirmed the overall stability of the formed AT11/ligand complexes within the MD time scale (Figure S20). MD outcomes also suggested the preferential interaction of bulbocapnine, chelidonine, dicentrine, and ibogaine with the 3′-end G-quartet of the AT11 G-quadruplex, although forming complexes with different features, as well as the preferential targeting of the region in proximity to the 5′-end G-quartet for rotenone, in full agreement with the NMR data.

### CD Studies of the Interactions between the Natural Compounds and AT11 G-Quadruplex

The interactions between bulbocapnine, chelidonine, dicentrine, ibogaine, and rotenone and the AT11 G-quadruplex were also investigated by CD analysis. AT11 solutions were prepared by overnight annealing the 28-mer samples at 2 μM DNA concentration in 70 mM KCl and 20 mM potassium phosphate buffer (pH 7.0). In full agreement with the NMR data, in the here used experimental conditions, AT11 adopted a parallel G-quadruplex topology,^15^ with a maximum centered at 262 nm and a minimum at 242 nm, as well as a shoulder at ca. 285 nm (Figure S21, black lines).

As far as the natural compounds are concerned, all of them presented one or more chiral centers and showed CD signals in the same range as the investigated oligonucleotide sequence. Bulbocapnine showed a minimum at 270 nm and a shoulder at ca. 300 nm (Figure S22A). Chelidonine exhibited a weak CD band in the explored concentration range with a minimum at ca. 294 nm (Figure S22B). Dicentrine showed a band with a minimum at about 307 nm (Figure S22C). CD features similar to those of chelidonine were observed for ibogaine (Figure S22D), which exhibited a weak minimum at ca. 280 nm. Finally, rotenone showed a minimum at 280 nm and a shoulder at about 303 nm (Figure S22E).

After CD characterization of the free AT11 G-quadruplex and natural compounds, the AT11 G-quadruplex was titrated with increasing amounts of each natural compound (up to 10 molar equivalents), and the corresponding CD spectra were recorded after each addition. In parallel, the CD spectra of each compound were recorded by adding increasing amounts of each ligand to the buffer alone, thus reproducing the above titration experiments but in the absence of the DNA oligonucleotide (Figure S22A-E). The contribution of each ligand was then subtracted from the CD spectra obtained upon titration of the AT11 G-quadruplex, thus obtaining a more accurate picture of the conformational changes of the DNA oligonucleotide induced by each ligand (Figure S21A-E).

A slight decrease of the CD signal intensity of the AT11 262 nm band was observed upon titration with bulbocapnine, chelidonine, dicentrine, and rotenone (Figure S21A,B,C,E). On the other hand, ibogaine did not induce relevant variations in the intensity of the CD signal of the AT11 G-quadruplex (Figure S21D).

In addition to CD titration experiments, CD melting experiments were performed on all the DNA/ligand systems in 70 mM KCl and 20 mM potassium phosphate buffer (pH 7.0) to evaluate the effects on the AT11 G-quadruplex thermal stability upon incubation with each natural compound. CD melting curves of the AT11 G-quadruplex in the absence or presence of each ligand (DNA/ligand 1:10 ratio) were recorded by following the CD changes at the wavelength of intensity maximum (262 nm) (Figure S23). A melting temperature (*T*_m_) of 46 °C was found for the free AT11 G-quadruplex. Essentially, no stabilizing effects on the AT11 G-quadruplex were observed upon binding of bulbocapnine, chelidonine, ibogaine, and rotenone (Δ*T*_m_ = +1 °C) (Figure S23A,B,D,E), while small stabilizing effects were found in the presence of dicentrine (Δ*T*_m_ = +3 °C) (Figure S23C). However, inspection of the melting curve shape suggested that the presence of bulbocapnine, chelidonine, ibogaine, and rotenone enhanced the cooperativity of the nucleobase recognition of the AT11 G-quadruplex structure. Moreover, an additional inflection point was observed in the melting curve of AT11 in the presence of dicentrine, indicating that the ligand was able to induce the formation of a highly stable secondary species, featured by a *T*_m_ value of 87 °C.

Altogether, CD titration and melting experiments further proved the binding of bulbocapnine, chelidonine, dicentrine, ibogaine, and rotenone to the AT11 G-quadruplex, showing that the investigated natural compounds do not perturb the overall parallel fold of the AT11 G-quadruplex as observed by NMR and MD simulations.

### Fluorescence Spectroscopy Studies of the Interactions between the Natural Compounds and AT11 G-Quadruplex

Fluorescence spectra of bulbocapnine, chelidonine, dicentrine, ibogaine, and rotenone were recorded at 2 μM concentration in 70 mM KCl, 20 mM potassium phosphate buffer (pH 7.0) solutions. Bulbocapnine, chelidonine, dicentrine, and ibogaine showed one strong emission band at 460, 330, 367, and 356 nm, respectively. Conversely, rotenone did not show appreciable fluorescence intensity, and thus, its interaction with AT11 G-quadruplex could not be analyzed using this technique.

Fluorescence titrations were carried out by adding increasing amounts of AT11 G-quadruplex, previously annealed in 70 mM KCl, 20 mM potassium phosphate buffer (pH 7.0), to the natural compound taken at a fixed concentration (i.e., 2 μM). Upon each addition, the corresponding fluorescence spectrum was recorded after stabilization of the signal (Figure 5A-D). Then, the fraction of bound ligand was calculated from the obtained fluorescence intensity values and plotted as a function of DNA concentration (Figure 5E,G,H). These data were then fitted with an independent and equivalent-sites model^40^ to calculate the binding constants and stoichiometries.

**Figure 5:**
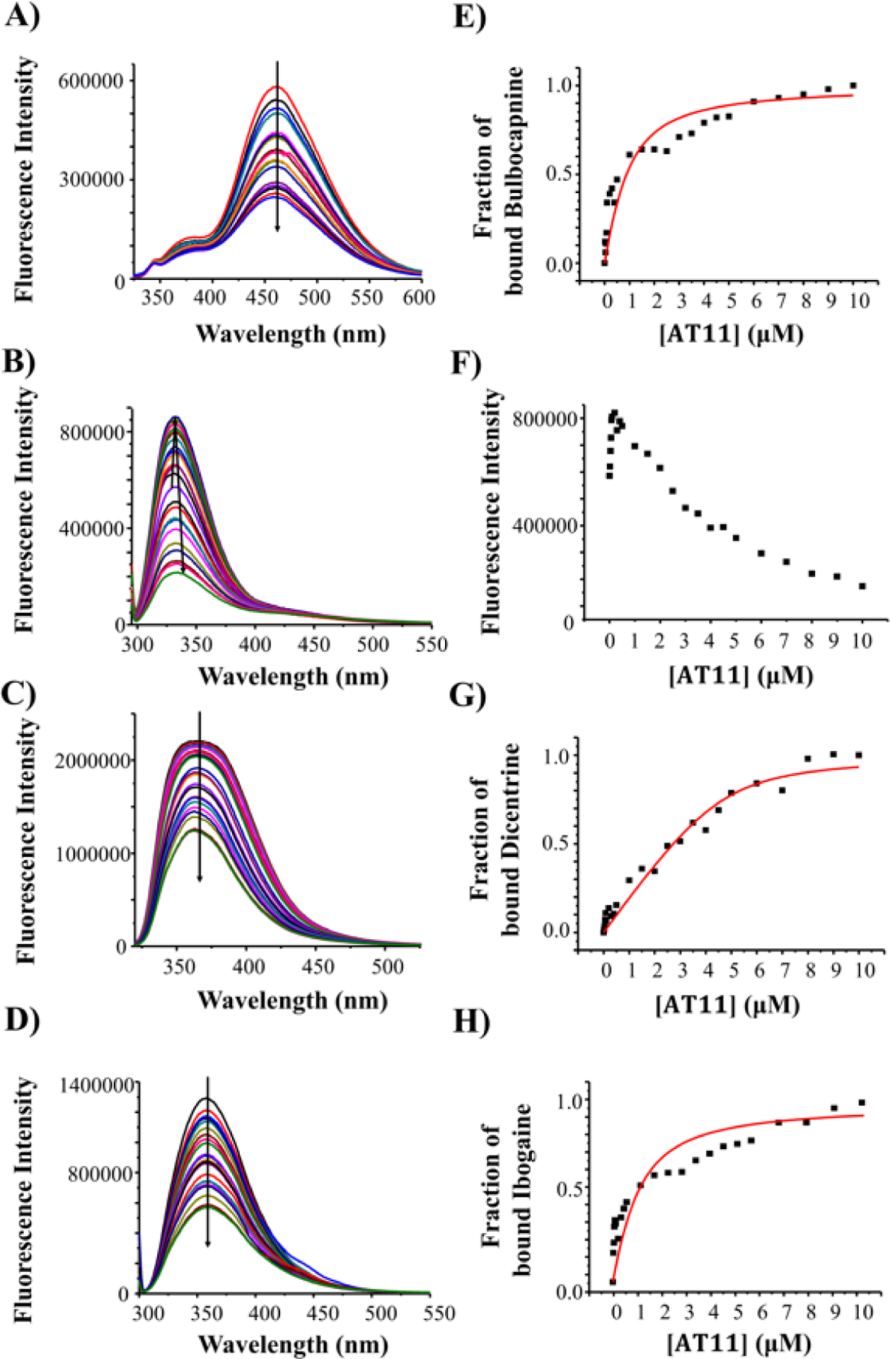
Left panels: Fluorescence emission spectra were obtained by adding increasing amounts of the AT11 G-quadruplex to 2 μM solutions of (A) bulbocapnine, (B) chelidonine, (C) dicentrine, and (D) ibogaine. Right panels: Representative binding curves obtained by plotting the fraction of bound ligand to the AT11 G-quadruplex as a function of the DNA concentration for (E) bulbocapnine, (G) dicentrine, and (H) ibogaine and a graph of fluorescence intensity vs. DNA concentration for (F) chelidonine. The black squares represent the experimental data; the red line represents the best fit obtained using an independent and equivalent-sites model.

Titrations of bulbocapnine, dicentrine, and ibogaine with increasing amounts of the AT11 G-quadruplex showed an overall fluorescence quenching (Figure 5A,C,D). Conversely, chelidonine titrations with the DNA oligonucleotide exhibited a complex behavior with an alternating trend (Figure 5B), as previously observed for this natural compound with other parallel G-quadruplex models.^29^ Indeed, plotting fluorescence intensity at 330 nm vs. added DNA increasing concentrations (Figure 5F), the obtained experimental data did not allow for applying a fitting protocol. Therefore, for the DNA/chelidonine system, neither binding constants nor stoichiometries could be determined. However, from the analysis of the fluorescence spectra, some information could be inferred. When chelidonine was mixed with the AT11 G-quadruplex, an overall fluorescence enhancement could be observed at DNA concentrations from 0 to 0.5 μM, while fluorescence quenching was found from 0.5 to 10 μM, suggesting the occurrence of multiple, different binding modes of chelidonine to the AT11 G-quadruplex as the DNA concentration.

On the other hand, the *K*_d_ values could be obtained for AT11/bulbocapnine (Figure 5E), AT11/dicentrine (Figure 5G), and AT11/ibogaine (Figure 5H) systems and are reported here in Table 1. From this analysis, a binding stoichiometry of 1:1 DNA/ligand was obtained for the AT11 G-quadruplex/dicentrine system, while for bulbocapnine and ibogaine, the fitting curves revealed higher binding stoichiometry values, i.e., 1:3 DNA/ligand (Table 1). Considering that multiple binding events occurred when bulbocapnine and ibogaine interact with AT11, as inferred by the obtained stoichiometry higher than 1:1 AT11/ligand and by the data points trend in Figure 5E,H, the obtained dissociation constant values should be considered as an average of all the constants associated with the multiple individual binding events involved in AT11/bulbocapnine and AT11/ibogaine complex formation.

**Table 1:** *K*_d_ and *n* Values for the Complexes between AT11 G-Quadruplex and Bulbocapnine, Dicentrine, or Ibogaine as Obtained by Fluorescence Titration Experiments

	*K*_d_ (μM)	*n*
**AT11/Bulbocapnine**	1.7 ± 0.3	3
**AT11/Dicentrine**	1.0 ± 0.7	1
**AT11/Ibogaine**	2.0 ± 0.4	3

Overall, all the compounds proved to strongly interact with the AT11 G-quadruplex. However, dicentrine showed a slightly higher affinity toward the AT11 G-quadruplex if compared with bulbocapnine and ibogaine, consistent with the higher stabilizing ability of dicentrine as determined by CD. Moreover, a specific binding site on the AT11 G-quadruplex was targeted by dicentrine, while three binding sites were occupied by bulbocapnine and ibogaine.

### Fluorescence Spectroscopy Studies on the Release of the Natural Compounds from the AT11 G-Quadruplex Complexes

In addition to the study of the binding of ligands to the AT11 G-quadruplex aptamer, an important point in assessing the efficacy of our AT11/ligand complexes as potential drug delivery systems is evaluating their ability to release the bound drug over time.

In this regard, the stability of the AT11/ligand complexes was tested using 70 mM KCl, 20 mM potassium phosphate buffer (pH 7.0) supplemented with fetal bovine serum (FBS), thus mimicking the physiological conditions and particularly the content of nucleases inside the cells, where the ligands should be released to exert their anticancer activity.

Fluorescence spectra were obtained in a buffer containing 10% FBS for solutions of free bulbocapnine, chelidonine, dicentrine, and ibogaine and 1:1 AT11/ligand complexes. Spectra were recorded immediately after the preparation of the samples and then after 2, 24, and 48 h incubation at 37 °C (Figure S24). The graphs of the fluorescence intensity for the 1:1 AT11/ligand complexes as a function of different conditions and incubation times are reported in Figure S25.

Notably, the quenching (for bulbocapnine, dicentrine, and ibogaine) or the enhancement (for chelidonine) of the ligand intrinsic fluorescence observed in the presence of 1 molar equivalent of AT11 G-quadruplex (Figure 5A-D) was also confirmed when in the buffer was present 10% FBS (Figures S24 and S25). After 2 h of incubation, a partial release of the ligand from the AT11/ligand complex was observed only in the case of bulbocapnine (Figure S25A). Upon 24 h of incubation, a complete release of the ligand was detected for ibogaine (Figure S25D), while the release of the ligand increased for chelidonine and dicentrine from 2 to 24 h of incubation (Figure S25B,C). After 48 h of incubation, a further release of the ligand was observed for dicentrine, which was almost completely released from AT11 (Figure S25C).

Overall, these data proved that the AT11 G-quadruplex is able to form stable complexes with the natural compounds investigated here as well as properly release the ligands more than 2 h after their incubation at 37 °C with FBS, suggesting that the ligands could be released precisely inside cells—where they are supposed to exert their anticancer activity by targeting telomeric and oncogenic G-quadruplexes—and are not lost from the AT11/ligand complexes before their entry into cancer cells.

### Biological Experiments on the Natural Compounds, AT11 G-Quadruplex, and Their Complexes

The ability of the natural compounds, AT11 G-quadruplex, and their complexes to induce antiproliferative effects on cancer cells, specifically using human gastric adenocarcinoma cells (AGS) as a model, was evaluated by MTT assay, in parallel using noncancerous transformed human cells as controls (HEK 293 and hTR8).

AT11 has been developed to recognize with high efficiency the protein nucleolin,^19,41–43^ which is overexpressed on the surface of several cancer cell lines including gastric cancer cells and specifically AGS cells,^44,45^ and whose content on normal cell surface is very low.^46^ Thus, exploiting the AT11-nucleolin recognition, the aptamer and its complexes with ligands can be selectively taken up into cancer cells.^13,44,47,48^ However, to prove that AT11 can be efficiently internalized in AGS cells in our specific experimental conditions, we first evaluated its capability to enter AGS cells by immunofluorescence assays. To this aim, AGS cells were treated for 72 h with 3 different concentrations (2, 10, and 50 μM, Figure 6) of Cy5-AT11, i.e., AT11 conjugated with the fluorescent label cyanine-5 at its 5′-end. Notably, Cy5-AT11 was found to be mainly located in the cytoplasm, confirming its effective entry into the AGS cells. Interestingly, at 10 and 50 μM Cy5-AT11 concentrations (Figure 6C,D), the nucleolin signal was more diffuse in the treated cells compared to untreated ones (Figure 6A and Table S6) and was mainly accumulated in the nuclei.

**Figure 6:**
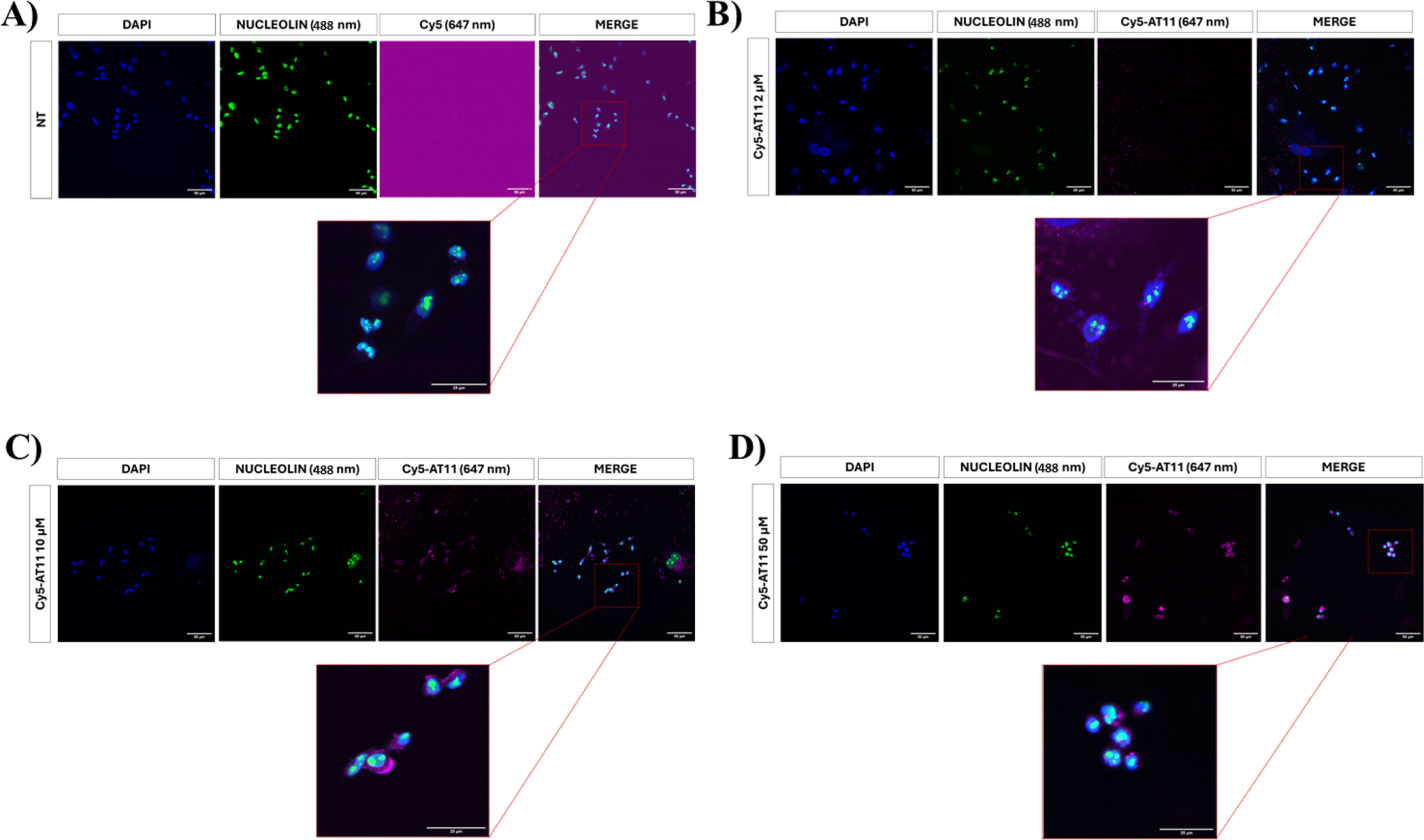
Immunofluorescence images of AGS cells (A) not treated (NT) or treated for 72 h with (B) 2 μM Cy5-AT11, (C) 10 μM Cy5-AT11, and (D) 50 μM Cy5-AT11. Nucleolin was stained with Alexa Fluor 488 antirabbit secondary antibody (green), while Cy5-AT11 was shown in violet (647 nm). Nuclei were stained with DAPI (blue). Scale bar: 50 or 25 μm. Acquisition: 20× (without zoom) or 20× (with 2× zoom) by an Olympus IX83 confocal microscope.

Then, cell viability experiments were performed by treating the cells with free AT11, bulbocapnine, chelidonine, dicentrine, ibogaine, and rotenone, and in parallel with Cy5-AT11 (Figure S26), at different concentrations (2, 10, 20, and 50 μM) for 24, 48, and 72 h (Figure S27A). AT11 and Cy5-AT11 reduced the cell viability by 50% or more at 20 and 50 μM concentrations from 24 h in the case of AT11 or 48 h in the case of Cy5-AT11. Moreover, higher effects were observed in the case of Cy5-AT11 than AT11 at all the tested concentrations upon 72 h incubation, while AT11 was more effective than Cy5-AT11 upon 24 h incubation. Overall, the decrease in cell viability upon treatment with both aptamers proved to be time- and dose-dependent.

In the treatments with the ligands, a time- and dose-dependent reduction of cell viability was observed as well. Generally, no relevant effects on cell viability were detected at 2 and 5 μM ligand concentrations over 72 h incubation, while at concentrations higher than 10 μM, the cell viability decrease was significant (Figure S27C-G). In detail, at a concentration of 50 μM and upon 72 h of incubation, a decrease in cell viability of 50% was observed for all ligands. Chelidonine and dicentrine emerged as the most effective ligands, showing stronger effects on cell viability than the other ligands of this series; indeed, a 50% decrease in cell viability was observed for these two ligands at 48 h and 20 μM concentration.

On the basis of the different active concentrations found for AT11 and ligands, as summarized in Table S7, in order to verify the effects of the AT11/natural compound complexes, cell viability assays were performed by treating AGS and HEK 293 or hTR8 cells for 72 h with the following combinations of compounds: AT11+bulbocapnine, AT11+ibogaine, and AT11+rotenone at a 1:1 ratio (each 50 μM) and AT11+chelidonine and AT11+dicentrine at a 1:1 ratio (each 10 μM) following the protocol reported in Figure 7A. All combinations showed a significant decrease in cancer cell viability at the concentrations used (Figure 7B). More notably, synergistic effects were detected for all complexes (Figure 7B), as also quantitatively estimated by exploiting the Bliss independence model (Table S8; for details, see Experimental section), if compared with the effects of free AT11 and free ligands (Figure 27B-G).

**Figure 7:**
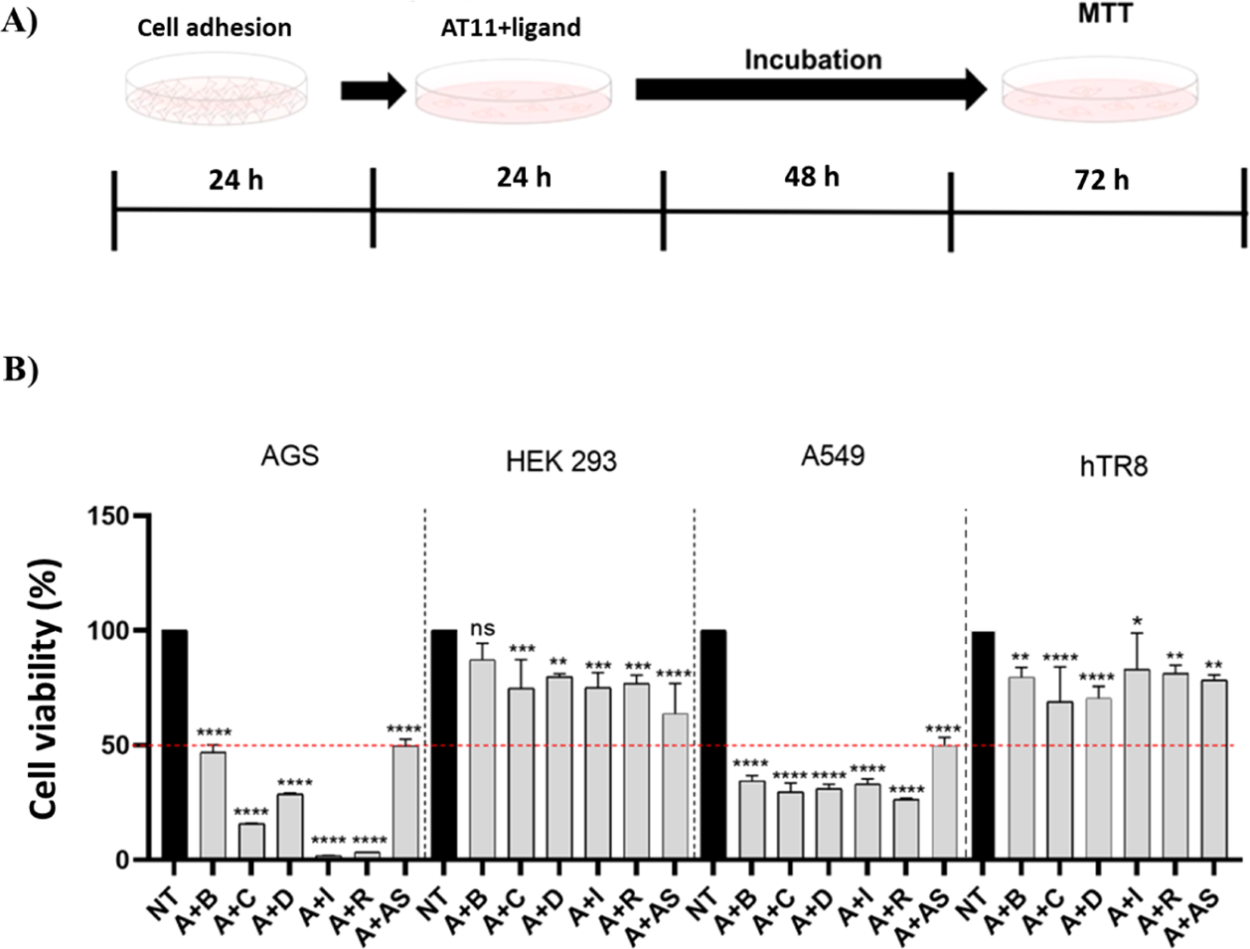
(A) Schematic representation of the experimental protocol adopted for the cell viability assay of AT11/ligand complexes. (B) AGS, HEK 293, A549, or hTR8 cells were treated for 72 h with AT11+bulbocapnine (A+B), AT11+ibogaine (A+I), and AT11+rotenone (A+R) at a 1:1 ratio (each 50 μM) and AT11+chelidonine (A+C), AT11+dicentrine (A+D), and AT11+aspidospermine at a 1:1 ratio (each 10 μM), and cell viability was measured by the MTT assay. Data are expressed as mean ± SD (standard deviation), *n* = 3. The significance was determined by one-way ANOVA, followed by Dunnett’s multiple comparison test; *­(*p* < 0.05),**­(*p* < 0.01),***­(*p* < 0.001), ****­(*p* < 0.0001), and ns (not significant). NT = not treated cells.

Moreover, the nucleolin content within AGS cells in the presence of free natural compounds and Cy5-AT11/natural compound complexes was quantified by immunofluorescence assays (Figures 8 and S28, and Table S6). Interestingly, the free compounds did not induce any increase in the nucleolin signal compared to the untreated cells, while a significant increase in nucleolin content within AGS cells was observed for the aptamer/ligand complexes, especially in the case of bulbocapnine, chelidonine, ibogaine, and rotenone, compared to both untreated cells and cells treated only with Cy5-AT11, thus further proving the higher and synergistic effects of AT11/ligand complexes compared to free AT11 and natural compounds.

**Figure 8:**
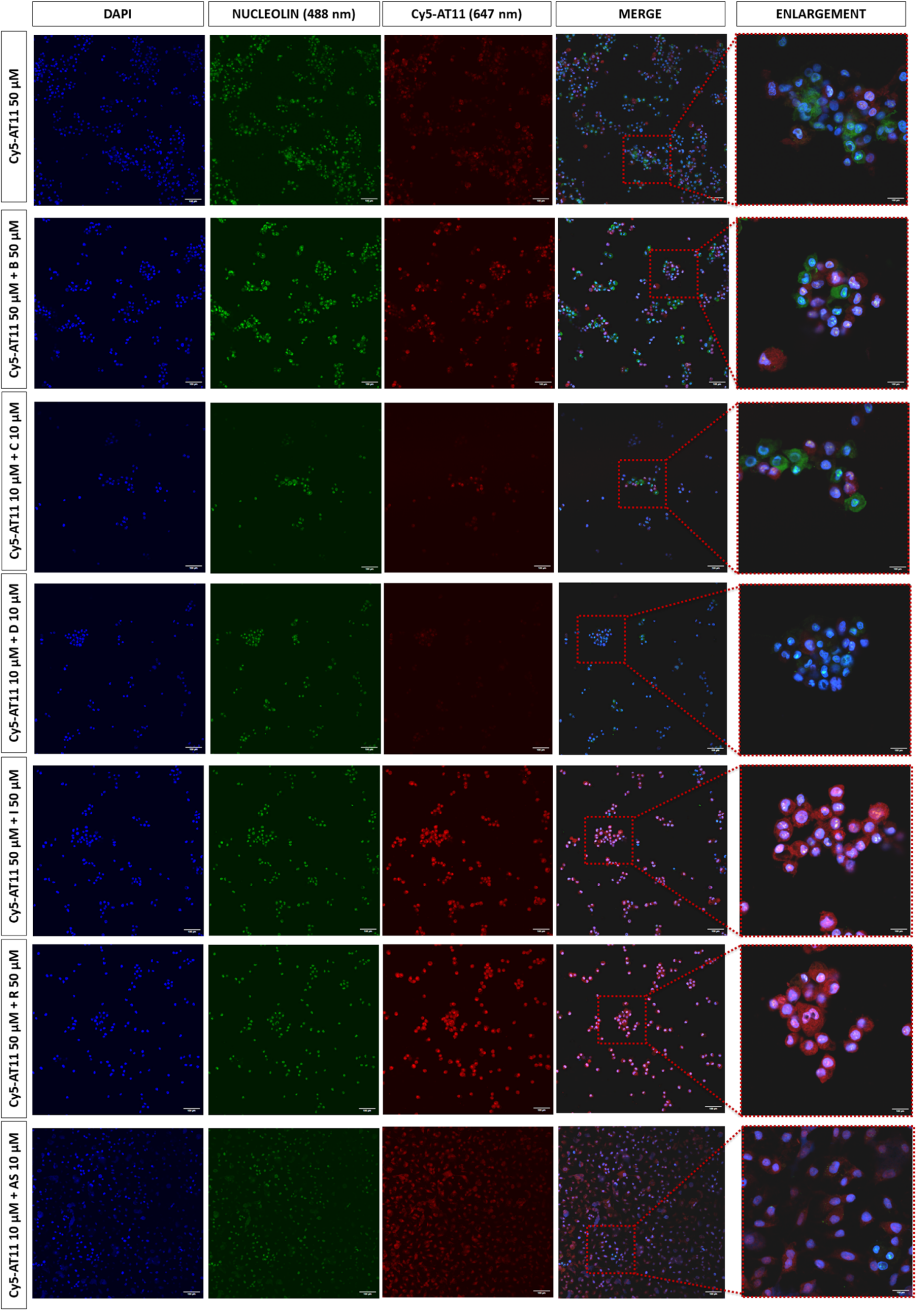
Immunofluorescence images of AGS cells treated for 72 h with the indicated concentrations of free Cy5-AT11 or Cy5-AT11 complexes with bulbocapnine (B), chelidonine (C), dicentrine (D), ibogaine (I), rotenone (R), and aspidospermine (AS). Nucleolin was stained with Alexa Fluor 488 antirabbit secondary antibody (green), while Cy5-AT11 was shown in red (647 nm). Nuclei were stained with DAPI (blue). Scale bar: 100 μm. Acquisition: 20× (without zoom) or 20× (with 4× zoom) by an Olympus IX83 confocal microscope.

In parallel, these confocal microscopy analyses allowed evaluation of the differential delivery of Cy5-AT11 or the natural compounds as free species or in the aptamer/ligand complexes (Figures 8 and S28, and Table S9). The Cy5-AT11 content within AGS cells proved to be higher when cells were treated with the Cy5-AT11/natural compound complexes compared to when treated only with the free Cy5-AT11. On the other hand, considering that the fluorescence of the natural ligands cannot be significantly distinguished from the rest of fluorescent entities in the cell, confocal microscopy experiments could not be helpful to directly explore the differential delivery of ligands as free or in complex with AT11. On this basis, we can state that synergistic effects are due to an increased delivery of the AT11 aptamer when in complex with the natural compounds, but in parallel we cannot exclude that an increased delivery of the natural compounds by AT11 is also occurring and determining the observed synergistic effects.

Notably, no relevant effect, and particularly no decrease in cell viability, was detected on noncancerous transformed cells in comparison to cancer cells (Figure 7B), denoting the importance of delivering the natural compounds by AT11 for a selective cytotoxic action of the anticancer drug/carrier systems against cancer vs. normal cells.

To further confirm the latter statement, the natural compound aspidospermine was introduced as a control in both MTT and immunofluorescence assays. This molecule has been here selected *ad hoc* since, although it was demonstrated to be an anticancer agent,^49^ we previously proved its inability to bind G-quadruplex structures.^29^ Notably, the MTT assay confirmed the anticancer efficacy of aspidospermine in AGS cells (Figure S27H and Table S7) and showed a similar cytotoxic activity to that found for the free compound when aspidospermine was incubated with the AT11 aptamer at a 1:1 ratio (Figure 7B). Moreover, the analysis based on the Bliss independence model (Table S8) proved that, contrary to the other investigated natural compounds, no synergistic but rather additive effects were observed for the AT11/aspidospermine 1:1 mixture compared to free AT11 and aspidospermine. Furthermore, the immunofluorescence assays proved that neither the free aspidospermine nor the 1:1 Cy5-AT11/aspidospermine mixture induced any increase in the nucleolin and Cy5-AT11 signals compared to both untreated cells and cells treated only with Cy5-AT11 (Tables S6 and S9, respectively), thus definitely proving the absence of synergistic effects for this compound. Altogether, these data demonstrate that compounds unable to bind G-quadruplex structures, and specifically the AT11 G-quadruplex, cannot benefit from the AT11 carrier system resulting in reduced effects on anticancer activity and related nucleolin targeting. In parallel, these results further prove that for the other investigated natural compounds able to bind the AT11 G-quadruplex, all the observed selective anticancer effects are strictly related to the improved cancer cell uptake mediated by the interaction with the AT11 aptamer.

Finally, the AT11/ligand complexes at a 1:1 ratio, using in parallel the AT11/aspidospermine 1:1 mixture as a control, were tested for their antiproliferative activity on an additional cancer cell line, i.e., human lung carcinoma cells (A549), to evaluate the possibility of extending the anticancer selective approach based on targeting the nucleolin protein overexpressed on the surface of cancer cells to other tumor forms beyond gastric adenocarcinoma (Figure 7B). Notably, the AT11/ligand complexes showed relevant antiproliferative activity on A549 cells, which was highly selective if compared to the activity found for each complex on HEK 293 or hTR8 cells. These findings proved that effective AT11-mediated recognition of nucleolin by the AT11 complexes, including G-quadruplex natural ligands, can occur on the surface of A549 cells similarly to the case of AGS cells. Remarkably, the lowest cancer vs. noncancerous cell selectivity was found for the AT11/aspidospermine 1:1 control mixture. Overall, lower anticancer effects were detected for A549 compared to AGS cells, except for the bulbocapnine and dicentrine complexes, showing a high sensitivity to our gastric systems compared to lung cancer cells.

## Conclusions

In the search for G-quadruplex-selective ligands as anticancer agents, we have recently evaluated libraries of natural compounds and discovered novel interactors of G-quadruplex structures.^28–31,50,51^ Specifically, the natural alkaloids bulbocapnine, chelidonine, dicentrine, ibogaine, and the natural isoflavone rotenone proved to strongly bind monomeric G-quadruplex models.^29,31,33^ Additionally, chelidonine, dicentrine, and rotenone showed anticancer activity in the low micromolar range, which correlated well with their targeting of telomeric and oncogenic G-quadruplex structures in cancer cells.^29,31^

Here, the interaction of these ligands with the AT11 aptamer, able to specifically recognize nucleolin overexpressed on the surface of cancer cells,^11–13^ was evaluated to exploit their binding to G-quadruplex-forming aptamers so as to obtain efficient drug/carrier systems for the selective delivery of G-quadruplex-targeting natural ligands into cancer cells.

This study proved that all the investigated natural compounds strongly interacted with the AT11 G-quadruplex without perturbing its overall parallel fold. In detail, bulbocapnine, chelidonine, dicentrine, and ibogaine showed a remarkable preference for the 3′-end G-quartet, whose binding involved a concomitant rearrangement of the flanking end residues. On the other hand, a preferential targeting of the region in proximity to the 5′-end G-quartet was observed for rotenone. Notably, although the primary binding sites of the ligands on the AT11 G-quadruplex were the two outer G-quartets, the grooves could also be involved in the binding when the ligand was in large excess.

Additionally, we proved that the AT11 G-quadruplex is able to properly release the bound ligands over time after more than 2 h from incubation at 37 °C with FBS, suggesting that the ligands could be released precisely inside the cells and are not lost from the AT11/ligand complexes before their entry into cancer cells. Thus, upon interaction of the complexes with nucleolin and their internalization, the complexes are expected to dissociate due to enzymatic digestion of the oligonucleotide aptamer,^52–54^ so that the ligands can be released and exert their anticancer activity by reaching the nucleus and interacting with their targets, i.e., telomeric and/or oncogenic G-quadruplexes.

Finally, the ability of the natural compounds, AT11 G-quadruplex, and their complexes, to induce antiproliferative effects on cancer cells, specifically using human gastric adenocarcinoma cells (AGS) as a model, was evaluated, using noncancerous transformed cells as control (HEK 293 and hTR8). AT11 G-quadruplex, as an AS1411 analogue, recognizes with high efficiency the protein nucleolin overexpressed on the surface of several cancer cell lines, and this recognition allows the selective uptake of the aptamer into cancer cells.^13,44,47,48^ Here, we proved that the AT11 G-quadruplex could be efficiently internalized also in AGS cells, being mainly localized in the cytoplasm, and promoted the entry of nucleolin into the nuclei, finally producing a time- and dose-dependent decrease in cell viability. As far as the free natural ligands are concerned, a time- and dose-dependent reduction of cell viability was observed with chelidonine and dicentrine emerging as the most effective ligands within this series. After evaluating the *in vitro* cytotoxicity of the free aptamer and free ligands, respectively, the antiproliferative effects of the AT11/ligand complexes on AGS and HEK 293 or hTR8 cells were tested. Notably, no relevant effect on cell viability was detected in noncancerous transformed cells, denoting the importance of delivering these natural compounds by means of the G-quadruplex-forming aptamer AT11, thus reducing potential toxic effects on normal cells. Moreover, all complexes caused a significant decrease in cancer cell viability at the tested concentrations compared with untreated cells. More notably, synergistic effects were detected for all complexes if compared with the effects on cell viability of the free aptamer and free ligands. The observed synergistic action can be attributed to (i) increased delivery of the ligand to the cells mediated by the AT11 G-quadruplex, which allows increasing the ligand concentration and effects in cancer cells based on the genomic G-quadruplex targeting upon release from the AT11 G-quadruplex, or (ii) increased delivery of the AT11 G-quadruplex when in complex with the natural compounds, which allows increasing both AT11 and ligand concentrations and effects inside cancer cells based on simultaneous targeting of nucleolin and genomic G-quadruplexes by AT11 and ligands, respectively. Moreover, a significant increase in nucleolin content within AGS cells was observed for the aptamer/ligand complexes compared with both untreated cells and cells treated only with Cy5-AT11, thus further proving the synergistic effects of AT11/ligand complexes compared to free AT11 and natural compounds.

Small molecules selectively binding to c-kit, telomeric, and bcl-2 G-quadruplexes were already known to be effective against GC cells.^37^ In detail, benzo­[a]­phenoxazines and quinazolone derivatives showed cytotoxic effects in HGC-27 cells by interacting with c-kit G-quadruplex and inhibiting oncogene transcription, while a 1,10-phenanthroline derivative produced DNA damage, telomere dysfunction, autophagy, and antitumor effects in AGS cells by stabilizing telomeric, c-kit, and bcl-2 G-quadruplexes.^55–57^ However, no indication of the negative effects that these potential drugs can cause in normal cells has been reported in these previous works.

Here, we provided for the first time evidence that G-quadruplex ligands, specifically of natural origin, can be selectively delivered by the AT11 aptamer to human gastric cancer cells exerting an effective anticancer activity accompanied by the absence of toxicity on noncancerous transformed cells. Therefore, AT11/G-quadruplex-targeting natural ligand complexes can be considered promising systems for the future development of alternative GC treatments endowed with low-to-null adverse effects. Additionally, we unraveled the molecular details of the binding of our natural ligands to the AT11 G-quadruplex, providing structural models of crucial importance also for the rational design of novel optimized ligands and/or derivatized aptamers to provide improved drug/aptamer complexes.

Noteworthy, the anticancer selective approach, here exploited for GC, based on targeting the nucleolin overexpressed on the surface of cancer cells by AT11/natural ligand complexes, can be extended to other tumor forms than gastric adenocarcinoma, such as human lung carcinoma, for which we here provided preliminary experimental evidence.

## Experimental Section

### Chemistry

All the tested ligands are known compounds belonging to an in-house library of natural products available from the Organic Chemistry Laboratory of the Department of Chemistry and Technology of Drugs of Sapienza University of Rome, Italy. The chemical identity of noncommercial compounds was assessed by rerunning NMR experiments and proved to be in agreement with the literature data. The purity of all compounds, checked by reversed-phase high-performance liquid chromatography (HPLC), was always higher than 95% (Figure S29).

Bulbocapnine hydrochloride (or (*S*)-11-methoxy-7-methyl-6,7,7a,8-tetrahydro-5*H*- [1,3]­dioxolo­[4’,5′:4,5]­benzo­[1,2,3-de]­benzo­[g]­quinolin-12-ol hydrochloride) was purchased from Sigma-Aldrich (CAS: 632-47-3, St. Louis, MO, USA) and used without further purification.

Chelidonine (or (5b*R*,6*S*,12b*S*)-13-methyl-5b,6,7,12b,13,14-hexahydro­[1,3]­dioxolo­[4’,5′: 4,5]­benzo­[1,2-*c*]­[1,3]­dioxolo­[4,5-i]­phenanthridin-6-ol) was purchased from Sigma-Aldrich (CAS: 476-32-4, St. Louis, MO, USA) and used without further purification.

Dicentrine (or (*S*)-10,11-dimethoxy-7-methyl-6,7,7a,8-tetrahydro-5*H*-[1,3]­dioxolo­[4‘,5′:4,5]­benzo­[1,2,3-de]­benzo­[g]­quinoline) was purchased from Biosynth Carbosynth and used without further purification.

Ibogaine (or (6*R*,7*S*,11*S*)-7-ethyl-2-methoxy-6,6a,7,8,9,10,12,13-octahydro-5*H*-6,9- methanopyrido­[1’,2’:1,2]­azepino­[4,5-*b*]­indole) showed NMR spectra identical to those reported in the literature for the pure compound.^58^

Rotenone (or (2*R*,6a*S*,12a*S*)-8,9-dimethoxy-2-(prop-1-en-2-yl)-1,2,12,12a-tetrahydrochromeno­[3,4-*b*]­furo­[2,3-h]­chromen-6­(6a*H*)-one) was purchased from Sigma-Aldrich (CAS: 83-79-4, St. Louis, MO, USA) and used without further purification.

Aspidospermine (or 1-((3a*R*,5a*R*,10b*R*,12b*R*)-3a-ethyl-7-methoxy-2,3,3a,5,5a,11,12,12b-octahydro-1*H*,4*H*-6,12a-diaza-indeno­[7,1-cd]­fluoren-6-yl)-ethanone) showed NMR spectra identical to those reported in the literature for the pure compound.^44^

The oligonucleotides AT11 and its derivative carrying cyanine 5 at the 5′-end were purchased from Biomers as HPLC-purified compounds with a purity >99%.

### NMR Spectroscopy

NMR data were collected on an Agilent NMR System 800 MHz and on Bruker AVANCE NEO 600 MHz NMR spectrometers. AT11 samples were prepared in 70 mM KCl, 20 mM potassium phosphate buffer (pH 7.0), 90%*/*10% H_2_O*/*D_2_O, at 0.1–0.3 mM oligonucleotide concentration per strand. In titration experiments, aliquots of the ligand stock solution (for bulbocapnine: 20 mM, H_2_O/D_2_O 90%/10%; for chelidonine, ibogaine, rotenone, and dicentrine: 20 mM, DMSO-d_6_) were directly added to the oligonucleotide solutions inside the NMR tube. NMR spectra were acquired with the use of the DPFGSE solvent suppression method. NOESY spectra were acquired at mixing times between 80 and 600 ms. DSS (4,4-dimethyl-4-silapentane-1-sulfonic acid) was used as a reference to calibrate the chemical shifts and its resonance set at 0.0 ppm. NMR spectra were processed and analyzed with the use of TopSpin 4.2.0 (Bruker) and Sparky (UCSF) software.

### Molecular Modeling

Molecular docking was performed with AutoDock 4.2.^38^ The first model of the NMR structure of AT11 (PDB ID 2N3M) was used as rigid receptor in docking simulations.^15^ The receptor grid was built to include all of the atoms of the NMR structure. A box of 90 × 90 × 90 points in the *xyz* space, with a spacing of 0.375 Å was used to this aim, in agreement with previous works.^29,31^ Default parameters of the genetic algorithm were used.

MD simulations were carried out with an AMBER20. The OL15 force field was used to parametrize the oligonucleotide, while the General Amber Force Field was used for small molecules.^59,60^ Each docking complex was included in a rectilinear box of TIP3P-type water molecules, buffering 10 Å from each complex, and the total charge was neutralized by adding K^+^ ions. Energy minimization was performed using a combination of steepest descent (SD) and conjugate gradient (CG) algorithms in a two-step approach: (i) energy minimization of the solvent while keeping the solute as frozen for 500 cycles SD followed by 2500 cycles CG; (ii) energy minimization of the solvated solute for 1000 cycles SD followed by 5000 cycles CG. Systems were then heated to 300 K with the Langevin thermostat at constant volume for 1 ns, and the density was subsequently equilibrated for 1 ns with the Berendsen barostat at constant pressure. A preliminary equilibration of 50 ns was run before the final production of MD trajectories acquired over 500 ns at constant pressure. In all MD simulations, no positional constraints were applied. The time step was 2 fs. Cluster analysis of MD trajectories was carried out with CPPTRAJ using a hierarchical agglomerative approach.^61^

### Circular Dichroism

CD spectra were recorded in a quartz cuvette with a path length of 1 cm on a Jasco J-715 spectropolarimeter equipped with a Peltier-type temperature control system (model PTC-348WI). The spectra were recorded at 20 °C in the range of 240–600 nm, with a 2 s response, a scanning speed of 200 nm/min, and a bandwidth of 2.0 nm, and were corrected by subtraction of the background scan with buffer. All the spectra were averaged over 3 scans. AT11 was dissolved in 70 mM KCl, 20 mM potassium phosphate buffer (pH 7.0), thus obtaining 2 μM solutions, which were then annealed by heating at 95 °C for 5 min, followed by slow cooling to room temperature. CD titrations were obtained by adding increasing amounts of the ligands (up to 10 molar equivalents, corresponding to a 20 μM solution in ligand) to the buffer alone or to the oligonucleotide solutions. For the CD melting experiments, the ellipticity was recorded at 262 nm with a temperature scan rate of 1 °C/min in the range of 10–105 °C.

### Fluorescence Spectroscopy

Fluorescence spectra were recorded at 20 °C on a HORIBA Jobin Yvon Inc. FluoroMax-4 spectrofluorometer equipped with an F-3004 Sample Heater/Cooler Peltier Thermocouple Drive, by using a quartz cuvette with a 1 cm path length. For the fluorescence titration experiments with bulbocapnine, chelidonine, dicentrine, and ibogaine, excitation wavelengths of 307, 289, 307, and 293 nm were used, respectively. The spectra were registered in the range of 315–600 nm for bulbocapnine, 295–550 nm for chelidonine, 320–600 nm for dicentrine, and 300–550 nm for ibogaine.

Titrations were carried out at a fixed concentration (2.0 μM) of the ligand. Increasing amounts of the AT11 G-quadruplex (up to 10 μM concentration) were added, taken from 120 μM annealed stock solutions of the DNA sample dissolved in 70 mM KCl, 20 mM potassium phosphate buffer (pH 7.0). After each addition, the system was allowed to equilibrate for 10 min before recording the spectra.

The fraction of bound molecules was calculated from the fluorescence intensity at 460 nm for bulbocapnine, 367 nm for dicentrine, and 356 nm for ibogaine and reported in a graph as a function of the DNA concentration. The fraction of the bound ligand was determined using the equation:

$$\alpha =\frac{Y-Y_{0}}{Y_{b}-Y_{0}}$$

where *Y*, *Y*_0_, and *Y*_b_ are the values of fluorescence emission intensity at the maximum at each titrant concentration, at the initial and final state of the titration, respectively. These points were fitted with an independent and equivalent-site model using the Origin 8.0 program.^40^

The equation of the independent and equivalent-sites model is as follows:

$$\alpha =\left(\frac{1}{2{[L]}_{0}}\right)\left\{({[L]}_{0}+n\left[\mathrm{DNA}\right]+\frac{1}{K_{b}})-\sqrt{{({\left[L\right]}_{0}+n[\mathrm{DNA}]+\frac{1}{K_{b}})}^{2}-4{[L]}_{0}n\left[\mathrm{DNA}\right]}\right\}$$

where α is the mole fraction of ligand in the bound form, [*L*]_0_ is the total ligand concentration, [DNA] is the added DNA concentration, *n* is the number of equivalent and independent sites on the DNA structure, and *K*_b_ is the binding constant.

For the ligand release experiments, fluorescence spectra of solutions of free bulbocapnine, chelidonine, dicentrine, and ibogaine and 1:1 AT11/ligand complexes were recorded in 70 mM KCl, 20 mM potassium phosphate buffer (pH 7.0) supplemented with 10% FBS. Spectra were recorded immediately after the preparation of the samples and also after 2, 24, and 48 h incubation at 37 °C. The data for the 1:1 AT11/ligand complexes were corrected by subtraction of the blank (buffer with 10% FBS) and accounting for ligand fluorescence intensity changes over time in the FBS buffer at 37 °C.

### Biological Experiments

#### Cell Cultures

Gastric adenocarcinoma cells (AGS), human embryonic kidney 293 cells (HEK 293), human lung carcinoma cells (A549), and human trophoblast cells (hTR8) were maintained at 37 °C in a humidified atmosphere of 95% air and 5% CO_2_ in Dulbecco’s Modified Eagle Medium low glucose (DMEM; Gibco) supplemented with 10% of heat-inactivated Fetal Bovine Serum (FBS; Gibco), 1% glutamine, and 1% antibiotics (100 U/mL Penicillin and 100 μg/mL Streptomycin; Gibco).

#### Cell Proliferation Assay

In the cell proliferation assay, AGS, HEK 293, A549, and hTR8 cells were cultured in a 96-well plate at a low density of 1200 cells/well. Each well contained 100 μL complete medium and was incubated at 37 °C, 5% CO_2_ for 18–20 h to allow cell adherence and growth.^62,63^ The medium was then replaced with a new complete medium containing AT11, bulbocapnine, chelidonine, dicentrine, ibogaine, or rotenone at concentrations ranging from 2 to 50 μM and analyzed at 24, 48, and 72 h. On the other hand, to test the synergistic effect of AT11 and the individual compounds, the medium was replaced with a new complete medium containing AT11 with bulbocapnine, chelidonine, dicentrine, ibogaine, or rotenone at a 1:1 AT11/ligand ratio and analyzed at 72 h. Both the aptamer and ligands were dissolved in 70 mM KCl and 20 mM potassium phosphate buffer (pH 7.0) before being tested in cells. The same protocols were used for aspidospermine used as the control.

Cell viability was assessed by the Cell Proliferation Assay, using MTT (3-(4,5-dimethylthiazol-2-yl)-2,5-diphenyltetrazolium bromide) (MTT assay) (Sigma-Aldrich) according to the manufacturer protocol. The absorbance was measured at 570 nm by using a Biotek Synergy microplate reader. All experiments were performed in triplicate. The effects on cell viability of the aptamer alone, of the free natural compounds, and of their complexes were assessed determining the percentage of viable cells compared to untreated control cells at time = 0.

IC_50_ values were estimated by using the following equation: IC_50_ = C1 + (50 – A1)­(C2 – C1)/(A2 – A1), where C1 and C2 are two consecutive compound concentrations such that the observed cytotoxic activity (with respect to the untreated control) A1 at the lower concentration C1 is higher than 50%, and the observed activity A2 at the higher concentration C2 is less than 50%.^64^

The Bliss independence model was used to evaluate synergistic, additive, or antagonistic effects for the investigated complexes. First, the viability of cells treated with each compound alone and in combination with the aptamer was measured and normalized to that of the untreated control (set at 100%). The effect of each treatment is then calculated using the following equation: E = 1 – (Viability_treated_)/(Viability_control_). In parallel, the observed combination effect (E_AB_) is calculated using the latter equation and replacing the measured viability of the combination. On the other hand, the expected combination effect is calculated using the following equation: E_Bliss_ = E_A_ + E_B_ −(E_A_E_B_), where E_A_ and E_B_ are the effects of compounds A and B alone. Comparison of the observed effect (E_AB_) with the Bliss expected value (E_Bliss_) allows classification of the observed antiproliferative activities as follows: (i) synergistic: E_AB_ > E_Bliss_; (ii) additive: E_AB_ = E_Bliss_; or (iii) antagonistic: E_AB_ < E_Bliss_.^65^

#### Immunofluorescence

In the immunofluorescence-based assay, AGS cells were cultured at a density of 5 × 10^4^ cells/cm^2^ and fixed directly on coated 13 mm glass coverslips in 24-multiwell plates by 4% paraformaldehyde (PFA) for 20 min at room temperature. Subsequently, cells were washed 3 times in 1× PBS. PBS was then removed, and cells were washed twice with freshly prepared IF Wash Buffer (1× PBS, 0.1% Tween 20). The samples were subsequently treated with IF Perm Buffer (1× PBS, 1% BSA, and 0.1% Triton X-100) for 40 min at room temperature. Then, incubation with the primary antibody was performed in IF Staining Buffer (1× PBS, 1% BSA) overnight at 4 °C. The day after, samples were washed in 1× PBS, 0.1% Tween 20, 5 times, and incubated with a secondary antibody (1:1000) in IF Staining Buffer for 2 h at room temperature. Samples were finally washed three times in 1× PBS, 0.1% Tween 20 for 10 min, counterstained with DAPI 1:1000 (4’,6’-diamidino-2-phenylindole) and mounted with Eukitt. The following primary antibodies were used: Antinucleolin, Rabbit polyclonal (Abcam, AMab129200) 1:100. The secondary antibody was conjugated with Alexa Fluor 488 (Invitrogen, 1:1000).

Immunoprofiled cultured AGS cells were photographed on an Olympus confocal microscope equipped with 4×, 10×, 20×, 40×, and 63× objectives. All images were processed by using ImageJ software.

#### Statistical Analyses

All statistical analyses were performed using Prism (GraphPad Software Inc., La Jolla, CA, USA). All data are the results of at least three independent experiments carried out in triplicate. Data were expressed as the means and standard deviations (SD).^66^ Comparisons among groups were made by analysis of variance ANOVA, followed by Dunnett’s or Tukey’s multiple comparison test. Values of *p* < 0.05 were considered significant.

## Supplementary Material







## Notes

Imino, aromatic, and methyl regions of ^1^H NMR spectra of free AT11 G-quadruplex and AT11/natural compound complexes; ^1^H NMR chemical shift differences for aromatic and methyl protons of free AT11 G-quadruplex and AT11/natural compound complexes; ^1^H NMR chemical shifts for free AT11 G-quadruplex and AT11/natural compound complexes; intermolecular NOE cross-peaks; RMSD values of MD simulations; CD spectra and CD-monitored melting curves; fluorescence spectroscopy data for FBS experiments; cell viability and immunofluorescence data for free AT11 G-quadruplex, free ligands, and AT11/ligand complexes; HPLC traces for the investigated natural compounds; (PDF)
Coordinates in PDB format of MD representative frames for the AT11/bulbocapnine, AT11/chelidonine, AT11/dicentrine, AT11/ibogaine, and AT11/rotenone complexes (ZIP)
Molecular formula strings for bulbocapnine, chelidonine, dicentrine, ibogaine, and rotenone, and related data (CSV)
