# Supplementary material for: Selective Delivery of Anticancer Natural G‑Quadruplex Ligands by the AT11 Aptamer for Gastric Cancer Treatment
Source: J Med Chem. 2025 Dec 15;69(1):352–67. doi: 10.1021/acs.jmedchem.5c02521 (PMC12794142; doi:10.1021/acs.jmedchem.5c02521)
Supplement: Supplementary file 1 [file jm5c02521_si_001.pdf]

Supporting Information

Selective delivery of anticancer natural G-quadruplex ligands by the AT11 aptamer for gastric cancer treatment

Chiara Platella,[1,*] Marko Trajkovski,[2] Mariarita Brancaccio,[3] Rosita Di Palma,[3] Andrea Calcaterra,[4] Mattia Mori,[5] Geppino Falco,[3] Janez Plavec,[2,6,7] Daniela Montesarchio[1,*]

[1]*Department of Chemical Sciences, University of Naples Federico II, via Cintia 21, 80126 Naples, Italy*

[2]*Slovenian NMR Centre, National Institute of Chemistry, Hajdrihova 19, 1000 Ljubljana, Slovenia*

[3]*Department of Biology, University of Naples Federico II, via Cintia 21, 80126 Naples, Italy*

[4]*Department of Chemistry and Technology of Drugs, Sapienza University of Rome, piazzale Aldo Moro 5, 00185 Rome, Italy*

[5]*Department of Biotechnology, Chemistry and Pharmacy, University of Siena, via Aldo Moro 2, 53100 Siena, Italy*

[6]*Faculty of Chemistry and Chemical Technology, University of Ljubljana, Večna Pot 113, 1000 Ljubljana, Slovenia*

[7]*EN-FIST Center of Excellence, Trg Osvobodilne Fronte 13, 1000 Ljubljana, Slovenia*

*Corresponding authors: Chiara Platella (chiara.platella@unina.it); Daniela Montesarchio (daniela.montesarchio@unina.it)

Contents

Figure S1. Imino regions of ^{1}H NMR spectra of AT11 G-quadruplex. S3
Figure S2. Aromatic regions of ^{1}H NMR spectra of AT11 G-quadruplex. S4
Figure S3. Methyl regions of ^{1}H NMR spectra of AT11 G-quadruplex. S5
Figure S4. Imino regions of ^{1}H NMR spectra of AT11 G-quadruplex titration with bulbocapnine. S6
Figure S5. Aromatic regions of ^{1}H NMR spectra of AT11 G-quadruplex titration with bulbocapnine. S7
Figure S6. Methyl regions of ^{1}H NMR spectra of AT11 G-quadruplex titration with bulbocapnine. S8
Figure S7. Imino regions of ^{1}H NMR spectra of AT11 G-quadruplex titration with chelidonine. S9
Figure S8. Aromatic regions of ^{1}H NMR spectra of AT11 G-quadruplex titration with chelidonine. S10
Figure S9. Methyl regions of ^{1}H NMR spectra of AT11 G-quadruplex titration with chelidonine. S11
Figure S10. Imino regions of ^{1}H NMR spectra of AT11 G-quadruplex titration with dicentrine. S12
Figure S11. Aromatic regions of ^{1}H NMR spectra of AT11 G-quadruplex titration with dicentrine. S13
Figure S12. Methyl regions of ^{1}H NMR spectra of AT11 G-quadruplex titration with dicentrine. S14
Figure S13. Imino regions of ^{1}H NMR spectra of AT11 G-quadruplex titration with ibogaine. S15
Figure S14. Aromatic regions of ^{1}H NMR spectra of AT11 G-quadruplex titration with ibogaine. S16
Figure S15. Methyl regions of ^{1}H NMR spectra of AT11 G-quadruplex titration with ibogaine. S17
Figure S16. Imino regions of ^{1}H NMR spectra of AT11 G-quadruplex titration with rotenone. S18

Figure S17. Aromatic regions of ^{1}H NMR spectra of AT11 G-quadruplex titration with rotenone. S19
Figure S18. Methyl regions of ^{1}H NMR spectra of AT11 G-quadruplex titration with rotenone. S20
Figure S19. ^{1}H NMR chemical shift differences for aromatic and methyl protons of AT11 G-quadruplex in the presence of different molar equivalents of the natural compounds. S21
Table S1. ^{1}H NMR chemical shifts of AT11 G-quadruplex. S22
Table S2. ^{1}H NMR chemical shifts of AT11 G-quadruplex in the presence of 1 molar equivalent of natural compound. S23
Table S3. ^{1}H NMR chemical shifts of AT11 G-quadruplex in the presence of 3 molar equivalents of natural compound. S24
Table S4. ^{1}H NMR chemical shifts of AT11 G-quadruplex in the presence of 6 molar equivalents of natural compound. S25
Table S5. Intermolecular NOE cross-peaks observed for the AT11/natural compound complexes. S26
Figure S20. RMSD values for the MD simulations of the AT11/natural compound complexes. S27
Figure S21. CD spectra of AT11 G-quadruplex in the presence of the natural compounds. S28
Figure S22. CD spectra of natural compounds. S29
Figure S23. Normalized CD melting curves of AT11/natural compound complexes. S30
Figure S24. Fluorescence emission spectra of free ligands and their complexes with AT11. S31
Figure S25. Fluorescence intensity for free ligands and their complexes with AT11. S32
Figure S26. Cell viability assay for Cy5-AT11 in AGS cells measured by MTT assay. S33
Figure S27. Cell viability assay for free AT11 and free ligands in AGS cells. S34
Figure S28. Immunofluorescence images of AGS cells non-treated or treated with free ligands. S35
Table S6. Intensity values measured in AGS cells non-treated or treated with free Cy5-AT11, free natural compounds or their complexes. S36
Table S7. IC_{50} values for free AT11 G-quadruplex and free ligands in AGS cells. S37
Table S8. Analysis of synergistic effects for the AT11/natural compounds complexes. S38
Table S9. Number of DAPI-positive Cy5-AT11-positive AGS cells. S39
Figure S29. HPLC traces for the natural compounds. S40

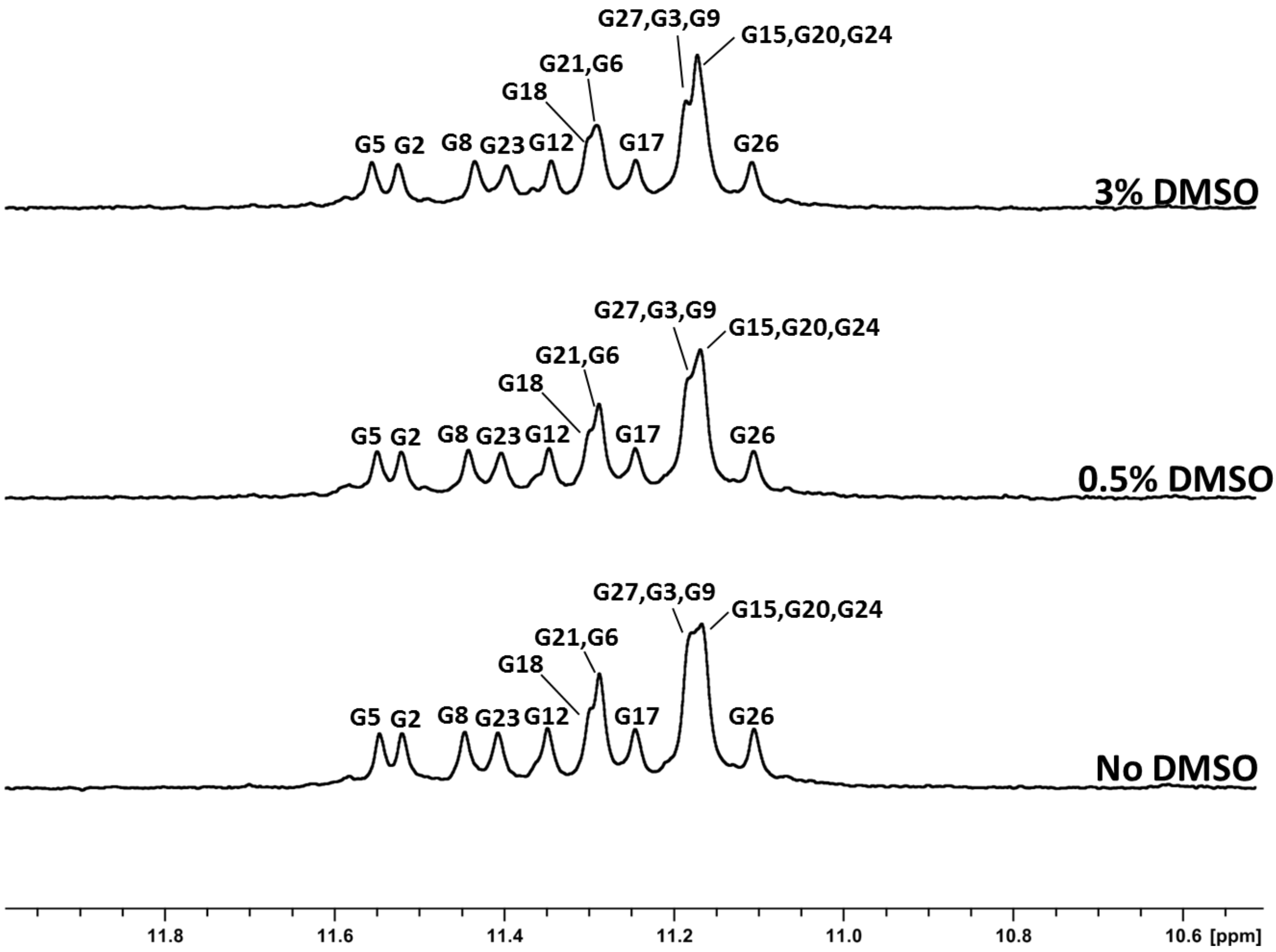

Figure S1. Imino regions of the ^{1}H NMR spectra of AT11 G-quadruplex in the absence and presence of small percentages of DMSO. DMSO percentages are shown on the right of the corresponding spectrum. The ^{1}H NMR spectra were recorded in 90%/10% H_2O/D_2O at 0.2 mM DNA, 70 mM KCl, 20 mM potassium phosphate buffer (pH 7.0) and 25 °C.

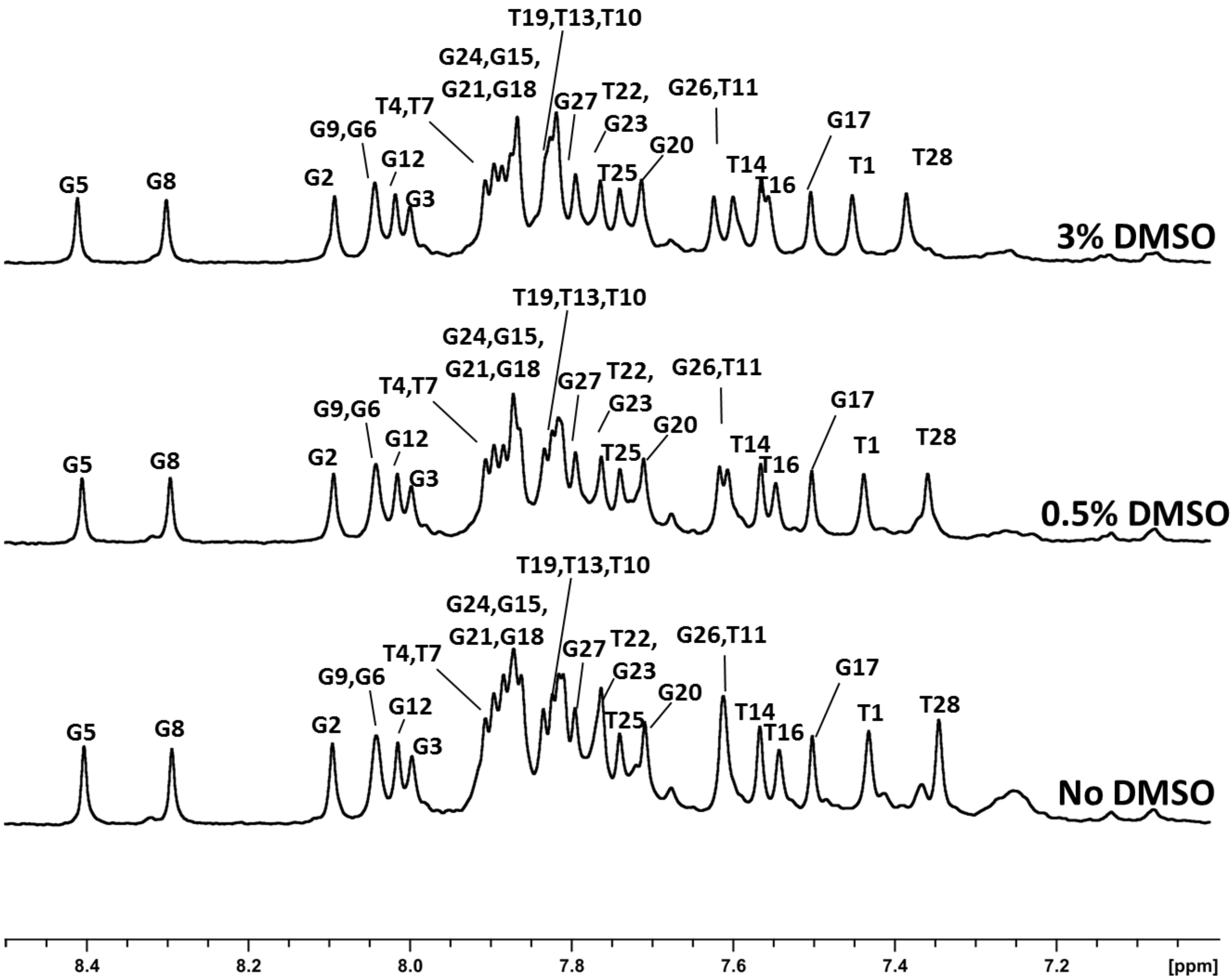

Figure S2. Aromatic regions of the ^{1}H NMR spectra of AT11 G-quadruplex in the absence and presence of small percentages of DMSO. DMSO percentages are shown on the right of the corresponding spectrum. The ^{1}H NMR spectra were recorded in 90%/10% H_2O/D_2O at 0.2 mM DNA, 70 mM KCl, 20 mM potassium phosphate buffer (pH 7.0) and 25 °C.

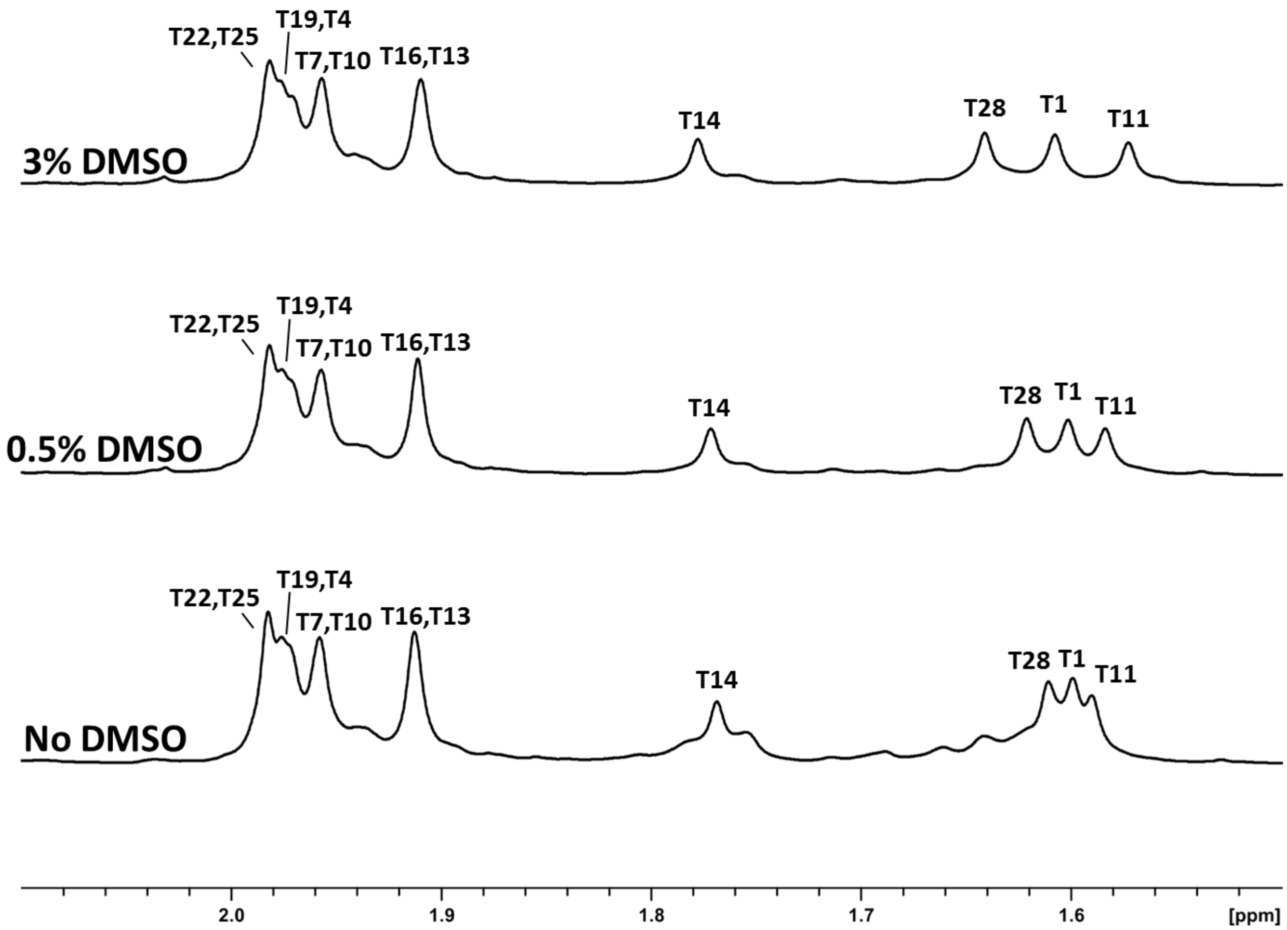

Figure S3. Methyl regions of the ^{1}H NMR spectra of AT11 G-quadruplex in the absence and presence of small percentages of DMSO. DMSO percentages are shown on the left of the corresponding spectrum. The ^{1}H NMR spectra were recorded in 90%/10% H_2O/D_2O at 0.2 mM DNA, 70 mM KCl, 20 mM potassium phosphate buffer (pH 7.0) and 25 °C.

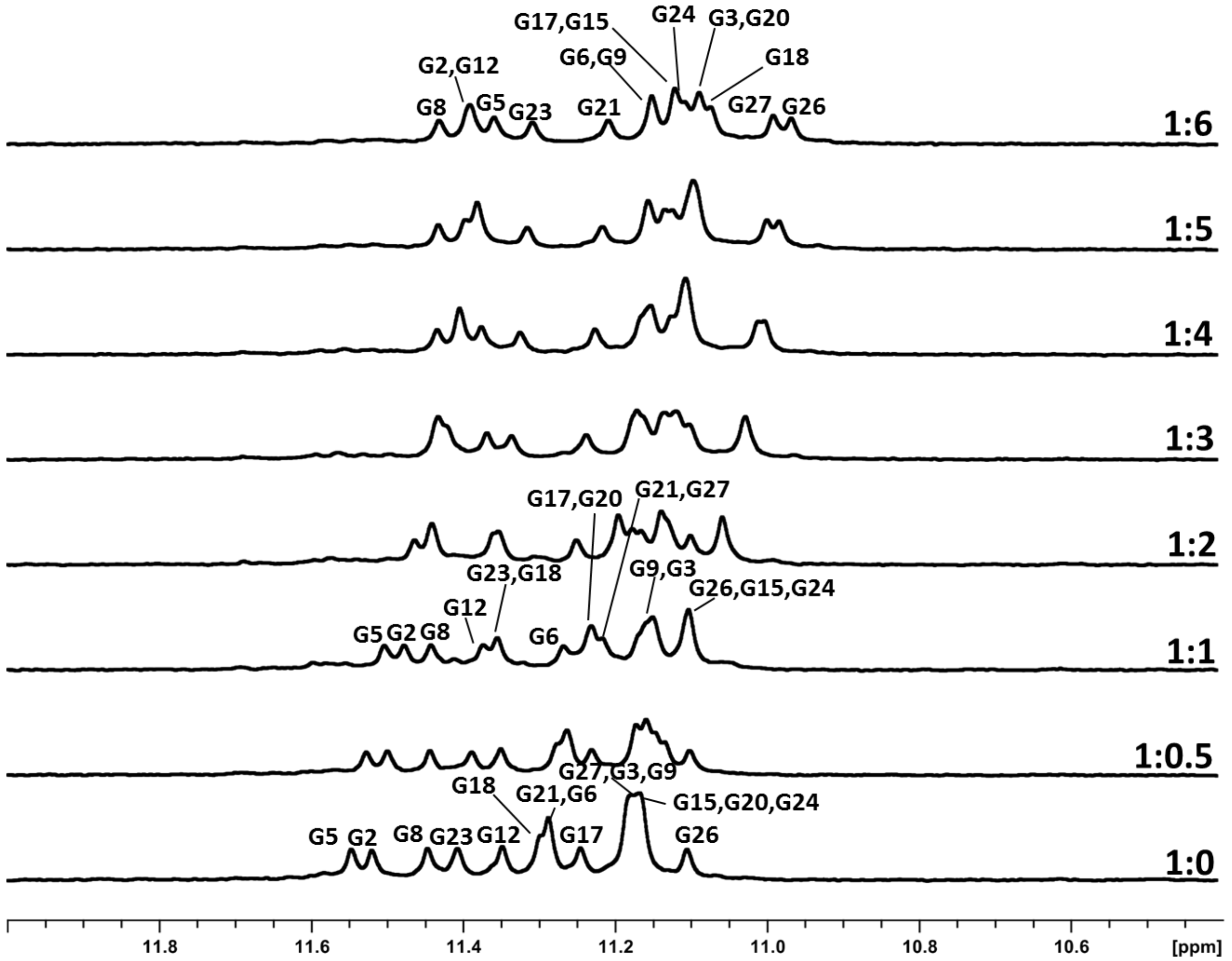

Figure S4. Imino regions of the ^{1}H NMR spectra of AT11 G-quadruplex upon titration with bulbocapnine (from 0.5 to 6 equivalents). AT11/bulbocapnine ratios are shown on the right of the corresponding spectrum. The ^{1}H NMR spectra were recorded in 90%/10% H_2O/D_2O at 0.2 mM DNA, 70 mM KCl, 20 mM potassium phosphate buffer (pH 7.0) and 25 °C.

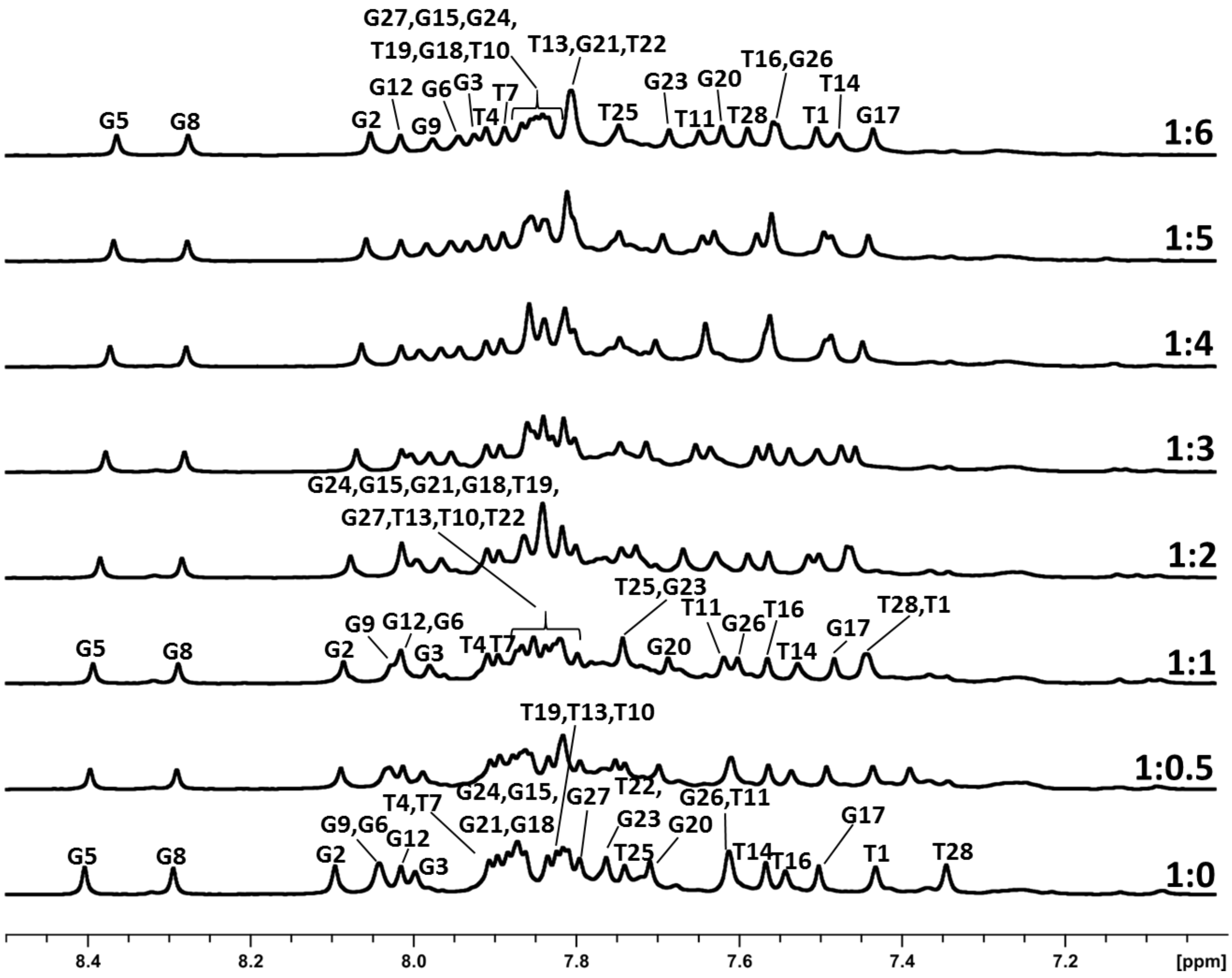

Figure S5. Aromatic regions of the ^{1}H NMR spectra of AT11 G-quadruplex upon titration with bulbocapnine (from 0.5 to 6 equivalents). AT11/bulbocapnine ratios are shown on the right of the corresponding spectrum. The ^{1}H NMR spectra were recorded in 90%/10% H_2O/D_2O at 0.2 mM DNA, 70 mM KCl, 20 mM potassium phosphate buffer (pH 7.0) and 25 °C.

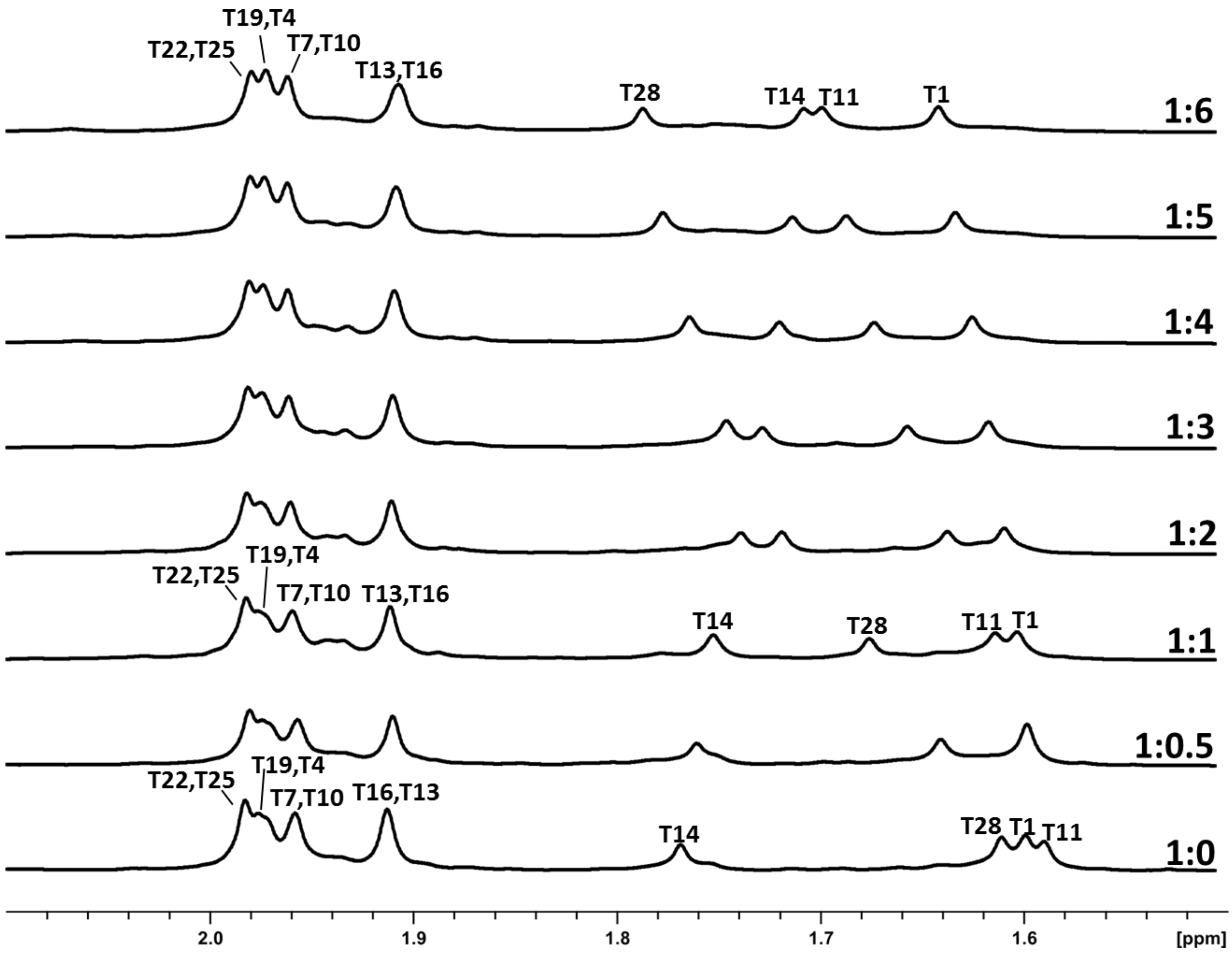

Figure S6. Methyl regions of the ^{1}H NMR spectra of AT11 G-quadruplex upon titration with bulbocapnine (from 0.5 to 6 equivalents). AT11/bulbocapnine ratios are shown on the right of the corresponding spectrum. The ^{1}H NMR spectra were recorded in 90%/10% H_2O/D_2O at 0.2 mM DNA, 70 mM KCl, 20 mM potassium phosphate buffer (pH 7.0) and 25 °C.

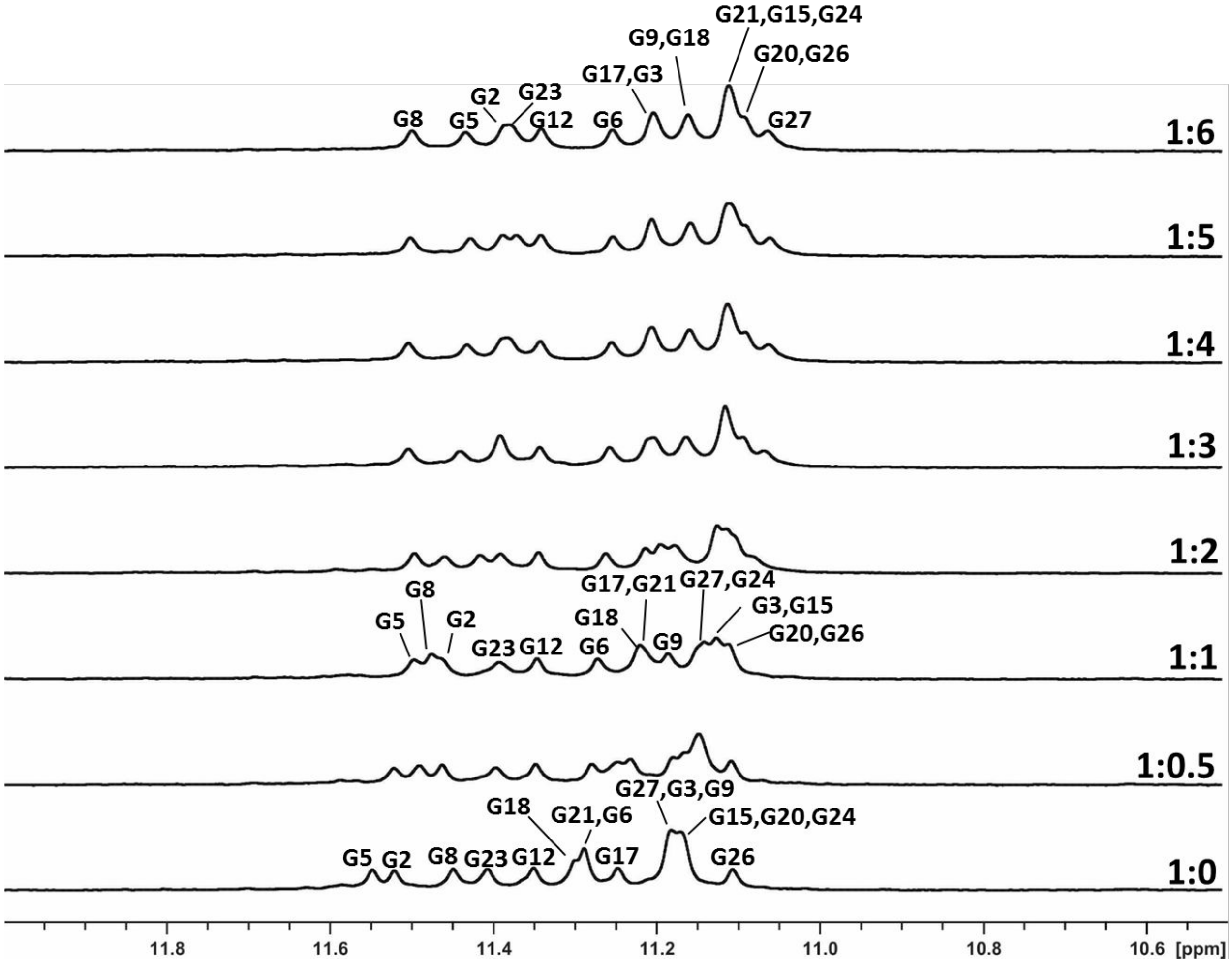

Figure S7. Imino regions of the ^1H NMR spectra of AT11 G-quadruplex upon titration with chelidonine (from 0.5 to 6 equivalents). AT11/chelidonine ratios are shown on the right of the corresponding spectrum. The ^1H NMR spectra were recorded in 90%/10% H_2O/D_2O at 0.2 mM DNA, 70 mM KCl, 20 mM potassium phosphate buffer (pH 7.0) and 25 °C.

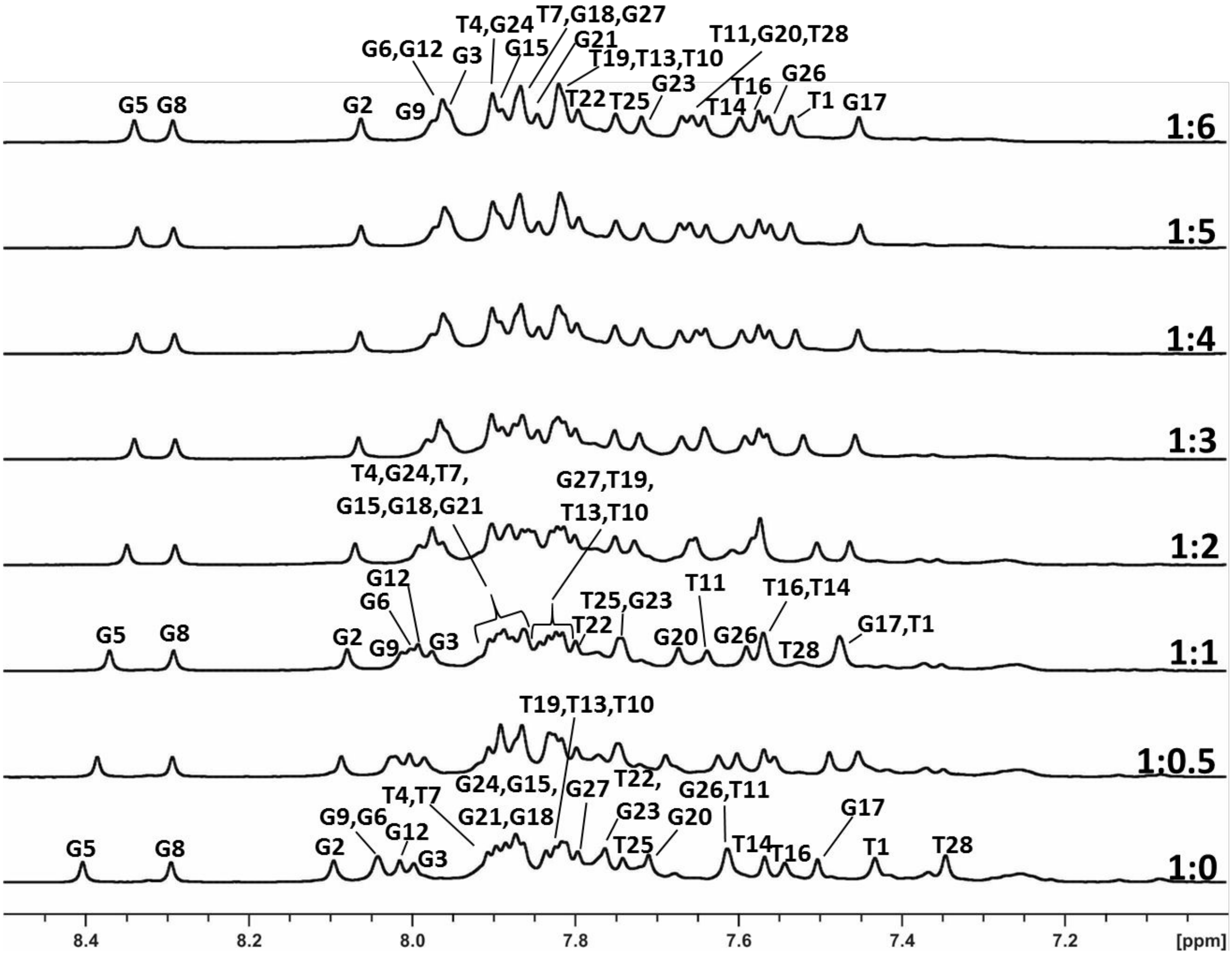

Figure S8. Aromatic regions of the ^1H NMR spectra of AT11 G-quadruplex upon titration with chelidonine (from 0.5 to 6 equivalents). AT11/chelidonine ratios are shown on the right of the corresponding spectrum. The ^1H NMR spectra were recorded in 90%/10% H_2O/D_2O at 0.2 mM DNA, 70 mM KCl, 20 mM potassium phosphate buffer (pH 7.0) and 25 °C.

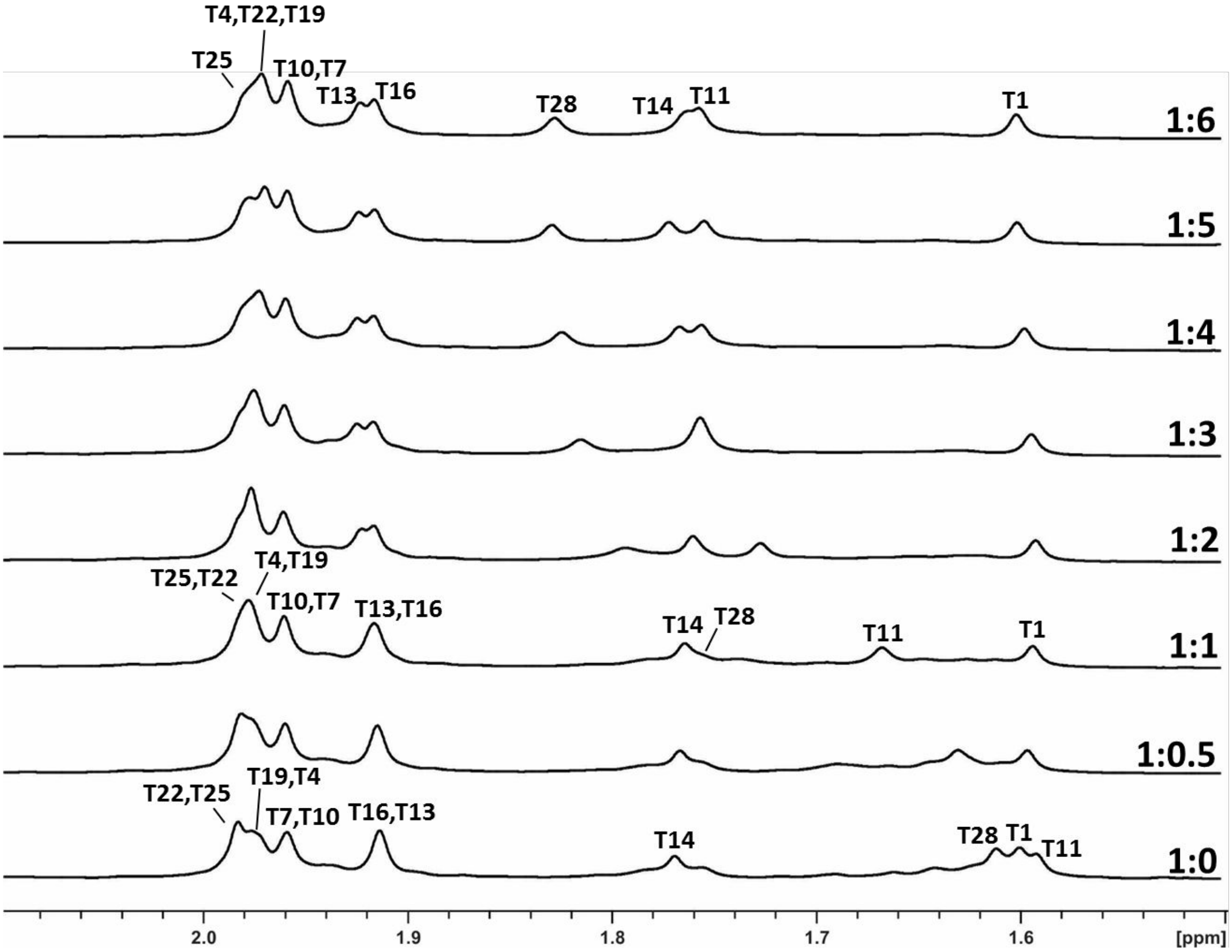

Figure S9. Methyl regions of the ^1H NMR spectra of AT11 G-quadruplex upon titration with chelidonine (from 0.5 to 6 equivalents). AT11/chelidonine ratios are shown on the right of the corresponding spectrum. The ^1H NMR spectra were recorded in 90%/10% H_2O/D_2O at 0.2 mM DNA, 70 mM KCl, 20 mM potassium phosphate buffer (pH 7.0) and 25 °C.

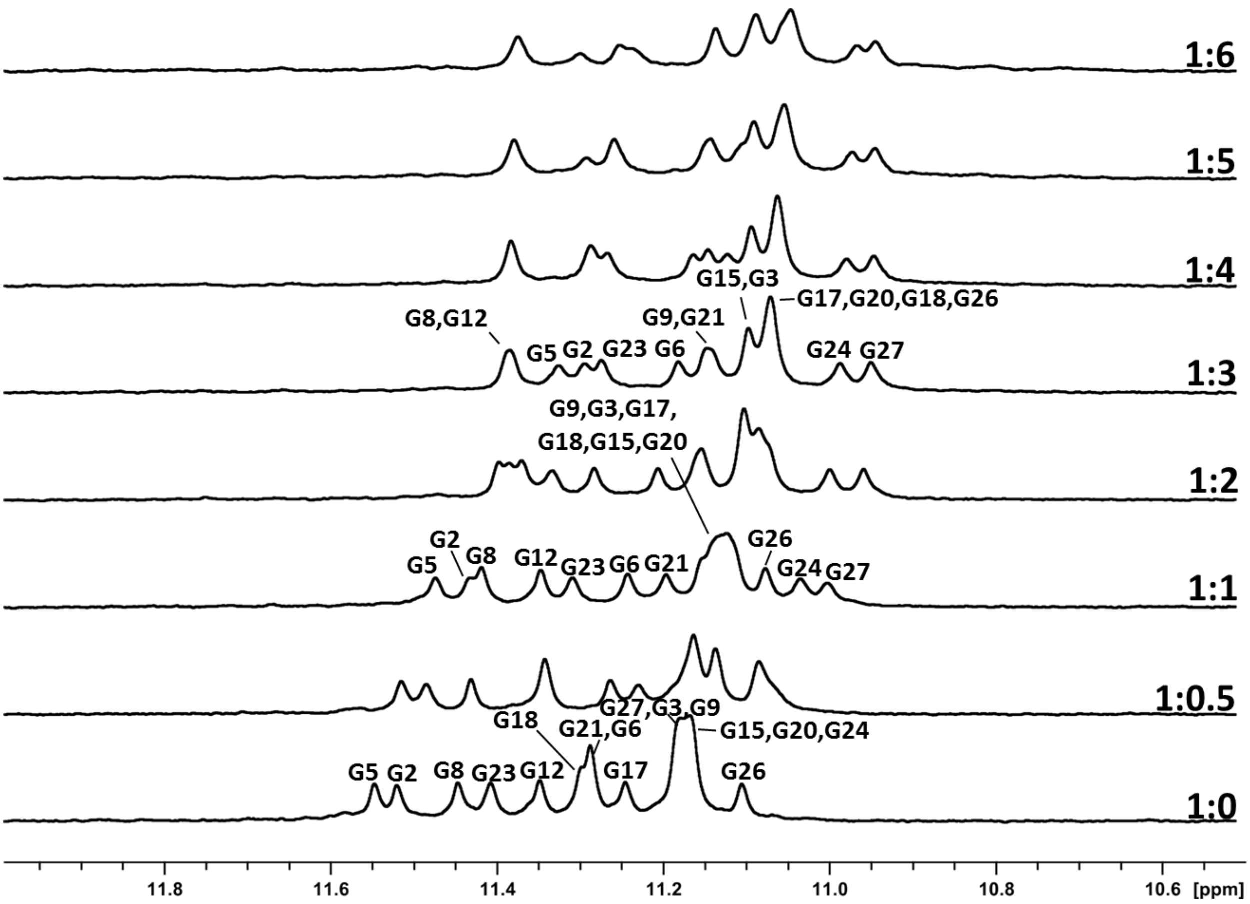

Figure S10. Imino regions of the ^{1}H NMR spectra of AT11 G-quadruplex upon titration with dicentrine (from 0.5 to 6 equivalents). AT11/dicentrine ratios are shown on the right of the corresponding spectrum. The ^{1}H NMR spectra were recorded in 90%/10% H_2O/D_2O at 0.2 mM DNA, 70 mM KCl, 20 mM potassium phosphate buffer (pH 7.0) and 25 °C.

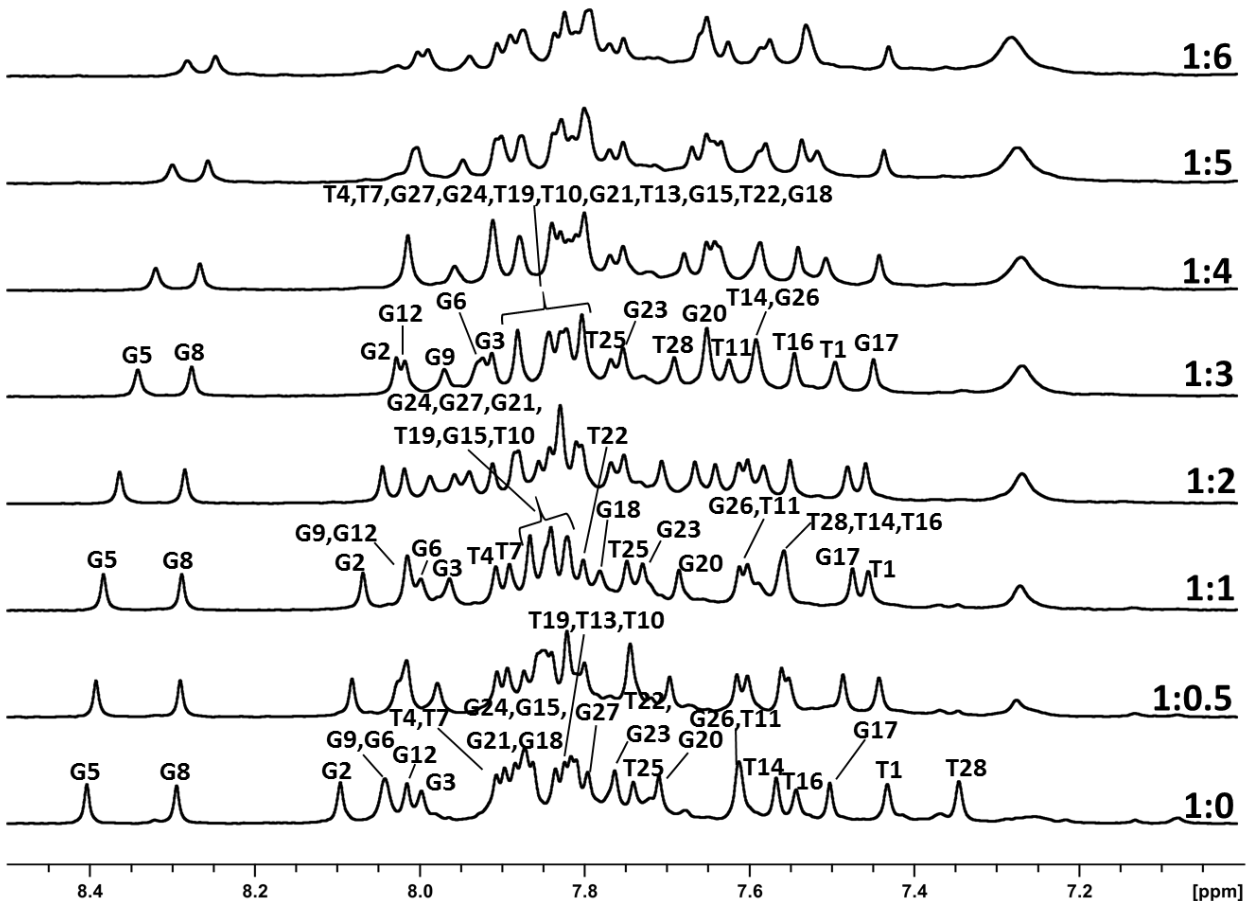

Figure S11. Aromatic regions of the ^{1}H NMR spectra of AT11 G-quadruplex upon titration with dicentrine (from 0.5 to 6 equivalents). AT11/dicentrine ratios are shown on the right of the corresponding spectrum. The ^{1}H NMR spectra were recorded in 90%/10% H_2O/D_2O at 0.2 mM DNA, 70 mM KCl, 20 mM potassium phosphate buffer (pH 7.0) and 25 °C.

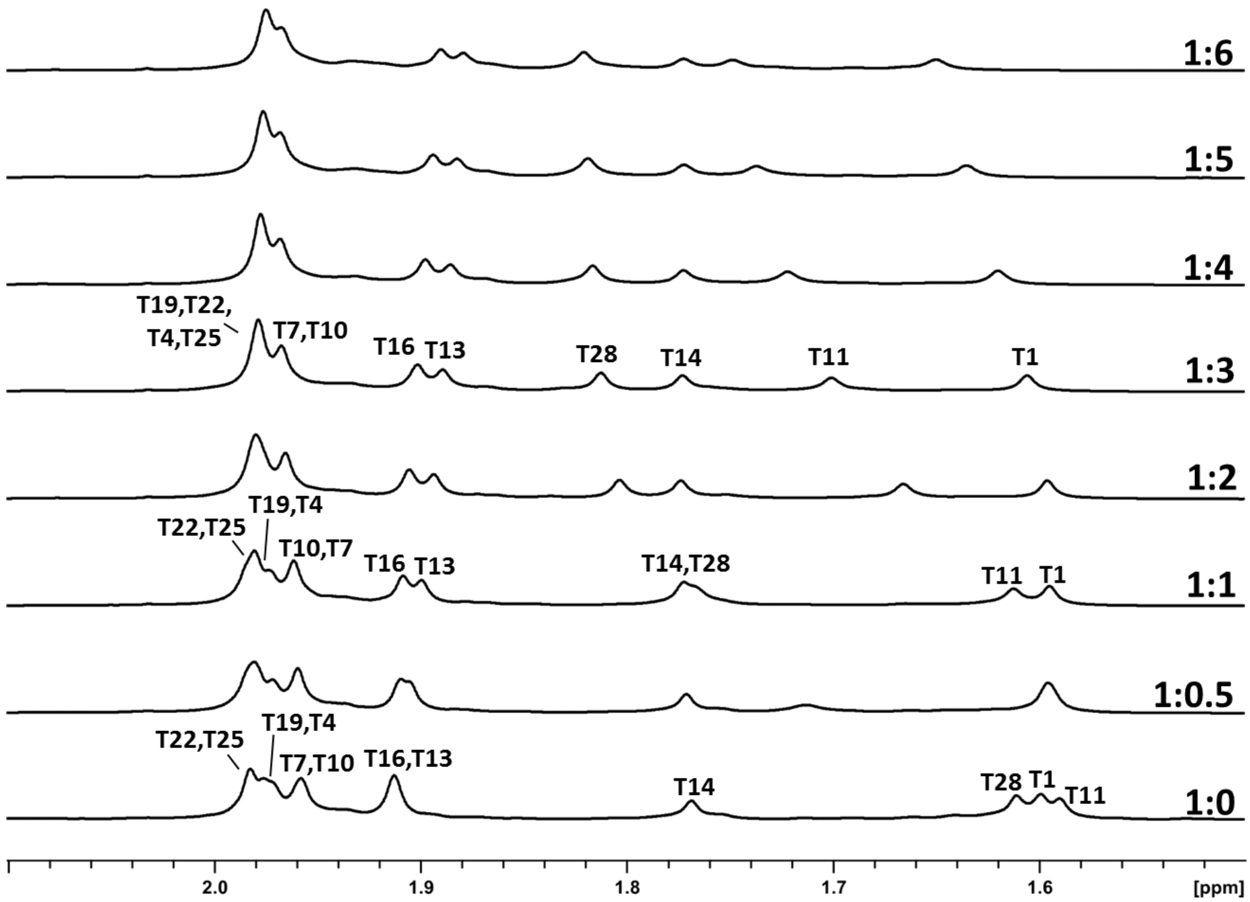

Figure S12. Methyl regions of the ^{1}H NMR spectra of AT11 G-quadruplex upon titration with dicentrine (from 0.5 to 6 equivalents). AT11/dicentrine ratios are shown on the right of the corresponding spectrum. The ^{1}H NMR spectra were recorded in 90%/10% H_2O/D_2O at 0.2 mM DNA, 70 mM KCl, 20 mM potassium phosphate buffer (pH 7.0) and 25 °C.

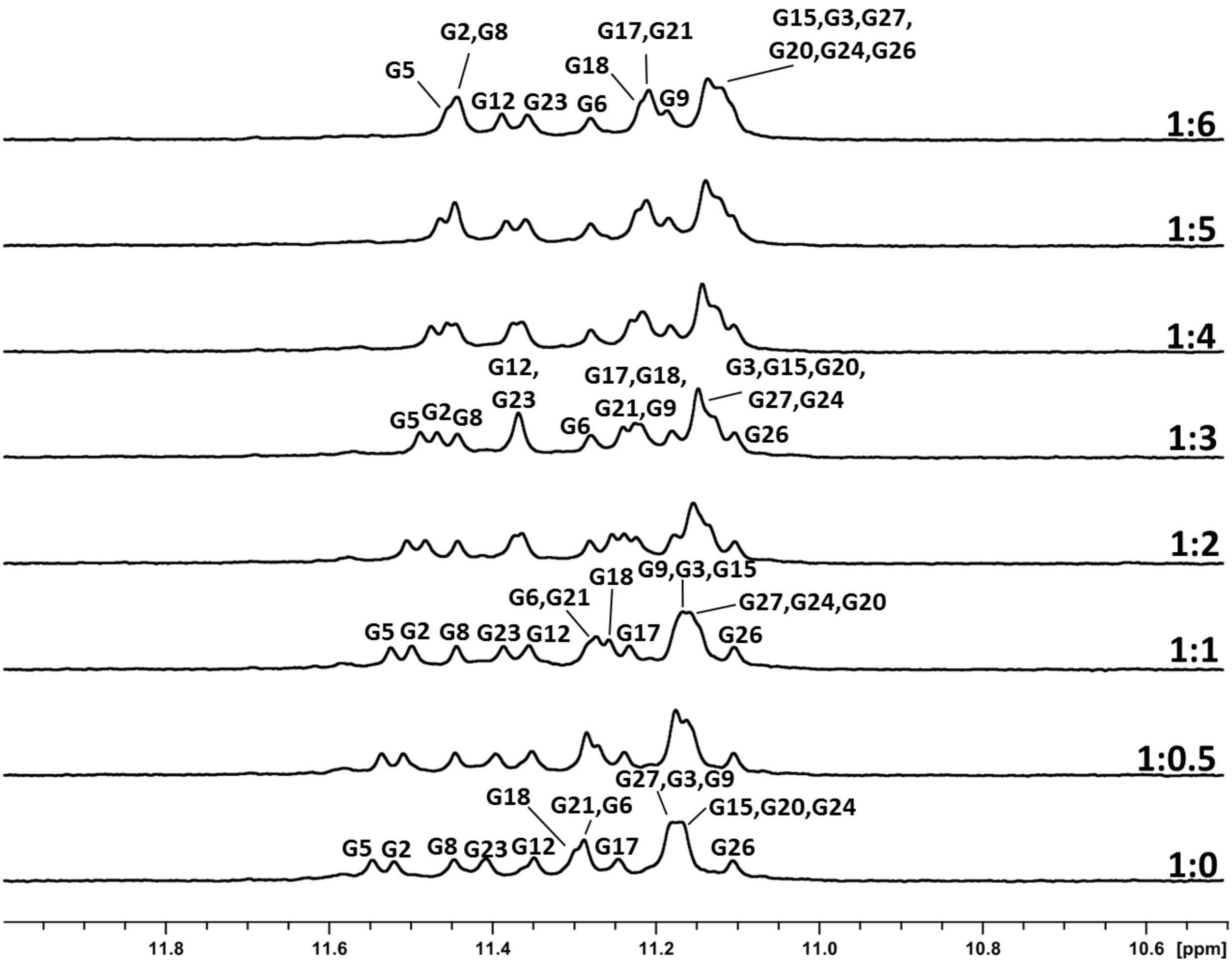

Figure S13. Imino regions of the ^{1}H NMR spectra of AT11 G-quadruplex upon titration with ibogaine (from 0.5 to 6 equivalents). AT11/ibogaine ratios are shown on the right of the corresponding spectrum. The ^{1}H NMR spectra were recorded in 90%/10% H_2O/D_2O at 0.2 mM DNA, 70 mM KCl, 20 mM potassium phosphate buffer (pH 7.0) and 25 °C.

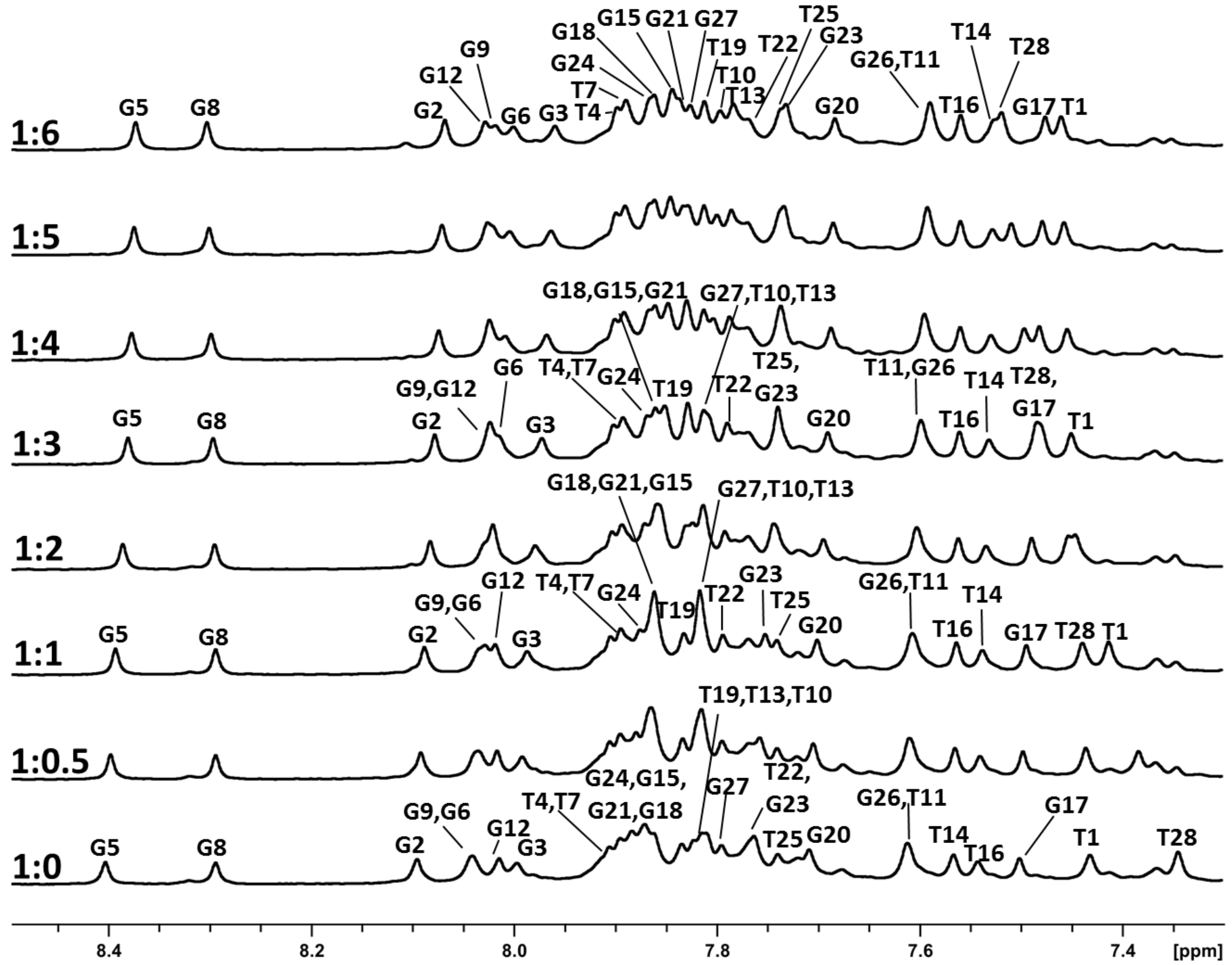

Figure S14. Aromatic regions of the ^{1}H NMR spectra of AT11 G-quadruplex upon titration with ibogaine (from 0.5 to 6 equivalents). AT11/ibogaine ratios are shown on the left of the corresponding spectrum. The ^{1}H NMR spectra were recorded in 90%/10% H_2O/D_2O at 0.2 mM DNA, 70 mM KCl, 20 mM potassium phosphate buffer (pH 7.0) and 25 °C.

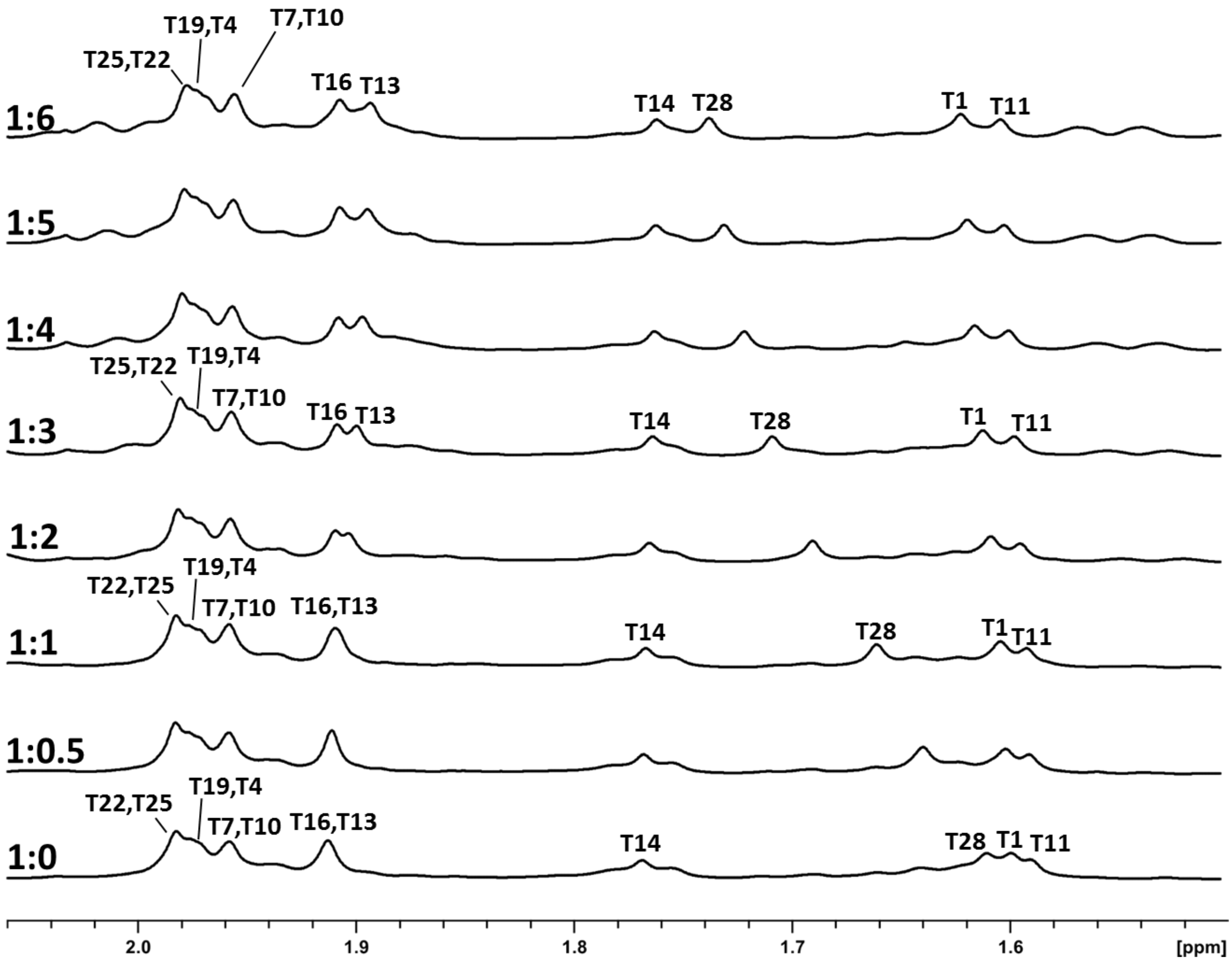

Figure S15. Methyl regions of the ^{1}H NMR spectra of AT11 G-quadruplex upon titration with ibogaine (from 0.5 to 6 equivalents). AT11/ibogaine ratios are shown on the left of the corresponding spectrum. The ^{1}H NMR spectra were recorded in 90%/10% H_2O/D_2O at 0.2 mM DNA, 70 mM KCl, 20 mM potassium phosphate buffer (pH 7.0) and 25 °C.

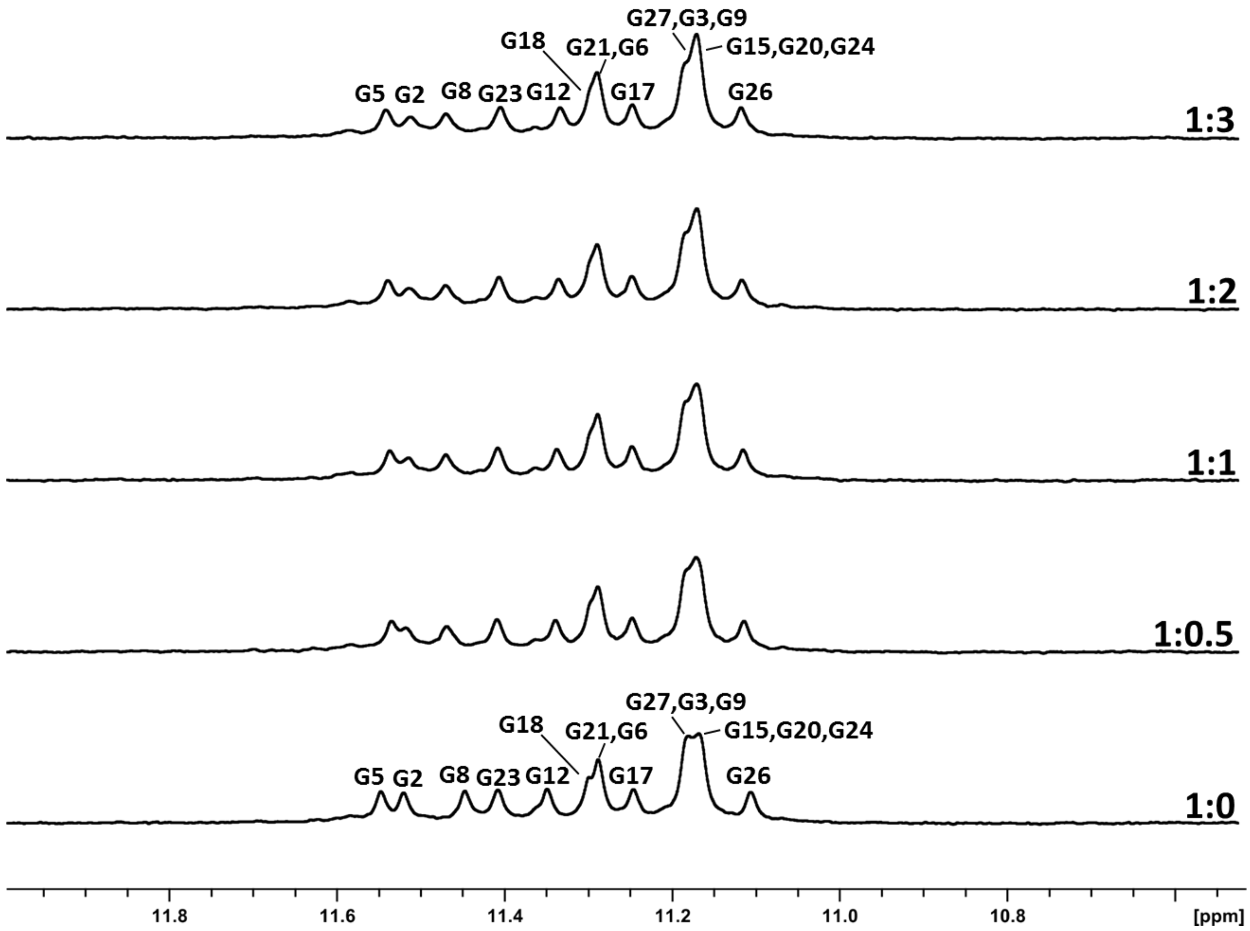

Figure S16. Imino regions of the ^{1}H NMR spectra of AT11 G-quadruplex upon titration with rotenone (from 0.5 to 3 equivalents). AT11/rotenone ratios are shown on the right of the corresponding spectrum. The ^{1}H NMR spectra were recorded in 90%/10% H_2O/D_2O at 0.2 mM DNA, 70 mM KCl, 20 mM potassium phosphate buffer (pH 7.0) and 25 °C.

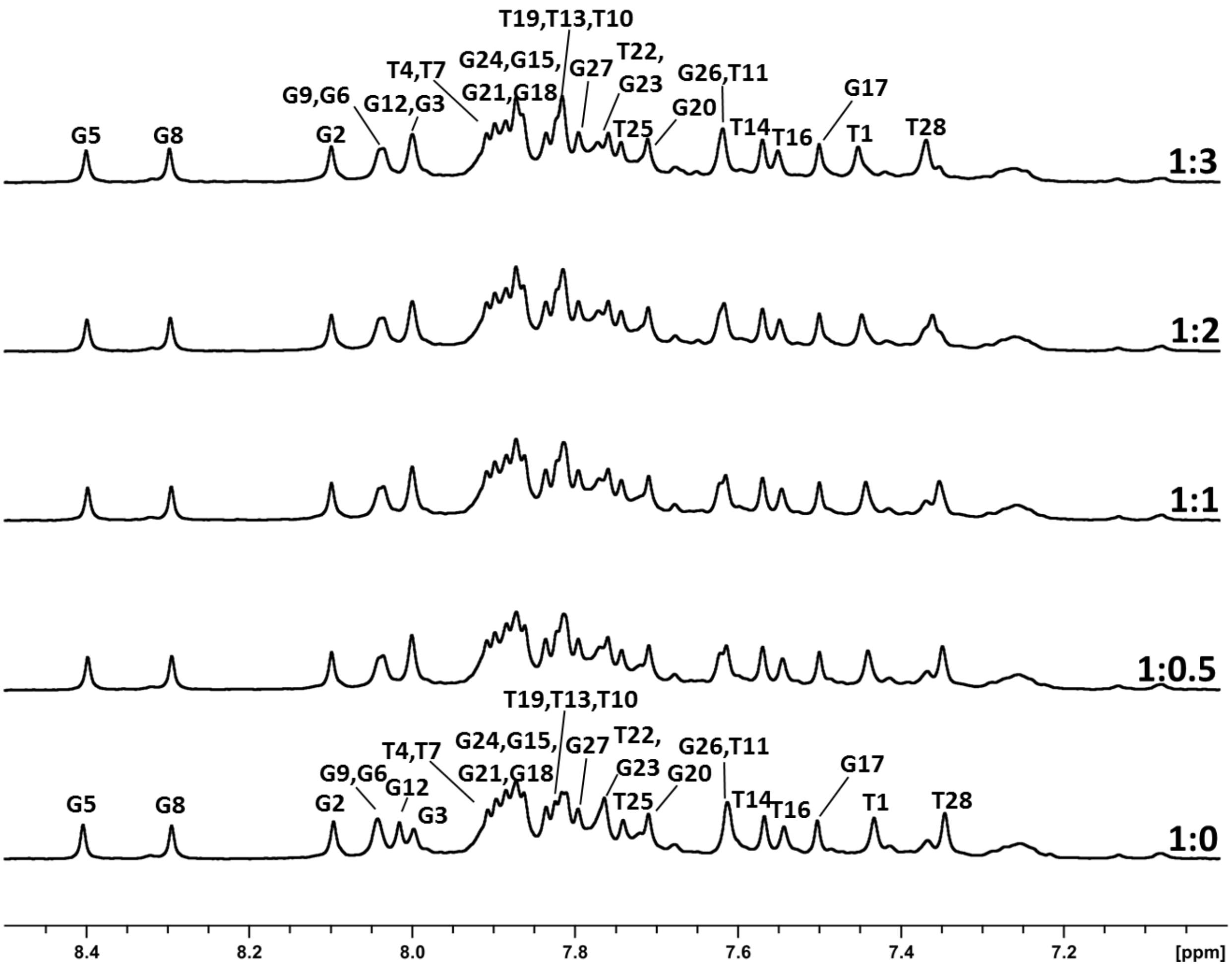

Figure S17. Aromatic regions of the ^{1}H NMR spectra of AT11 G-quadruplex upon titration with rotenone (from 0.5 to 3 equivalents). AT11/rotenone ratios are shown on the right of the corresponding spectrum. The ^{1}H NMR spectra were recorded in 90%/10% H_2O/D_2O at 0.2 mM DNA, 70 mM KCl, 20 mM potassium phosphate buffer (pH 7.0) and 25 °C.

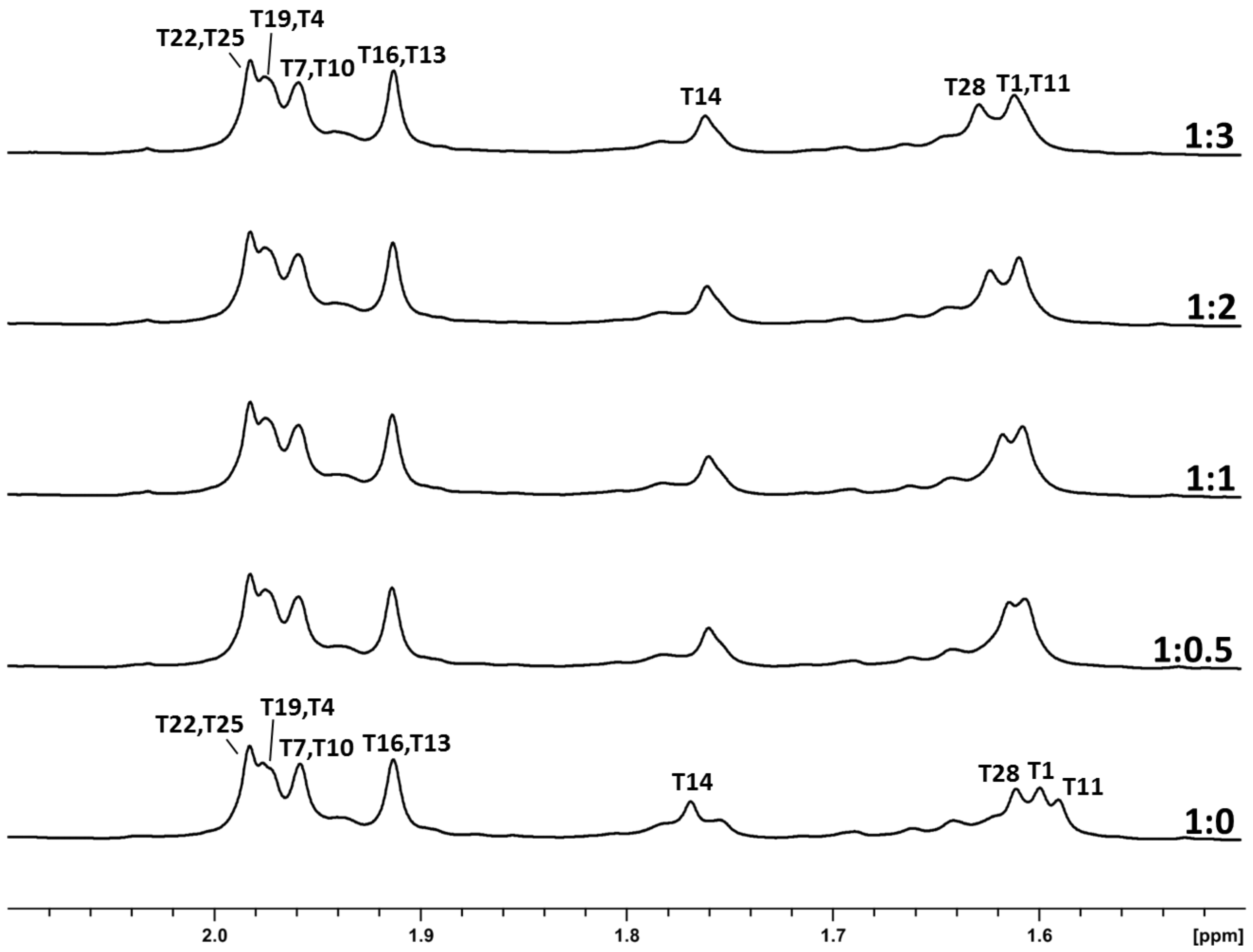

Figure S18. Methyl regions of the ^{1}H NMR spectra of AT11 G-quadruplex upon titration with rotenone (from 0.5 to 3 equivalents). AT11/rotenone ratios are shown on the right of the corresponding spectrum. The ^{1}H NMR spectra were recorded in 90%/10% H_2O/D_2O at 0.2 mM DNA, 70 mM KCl, 20 mM potassium phosphate buffer (pH 7.0) and 25 °C.

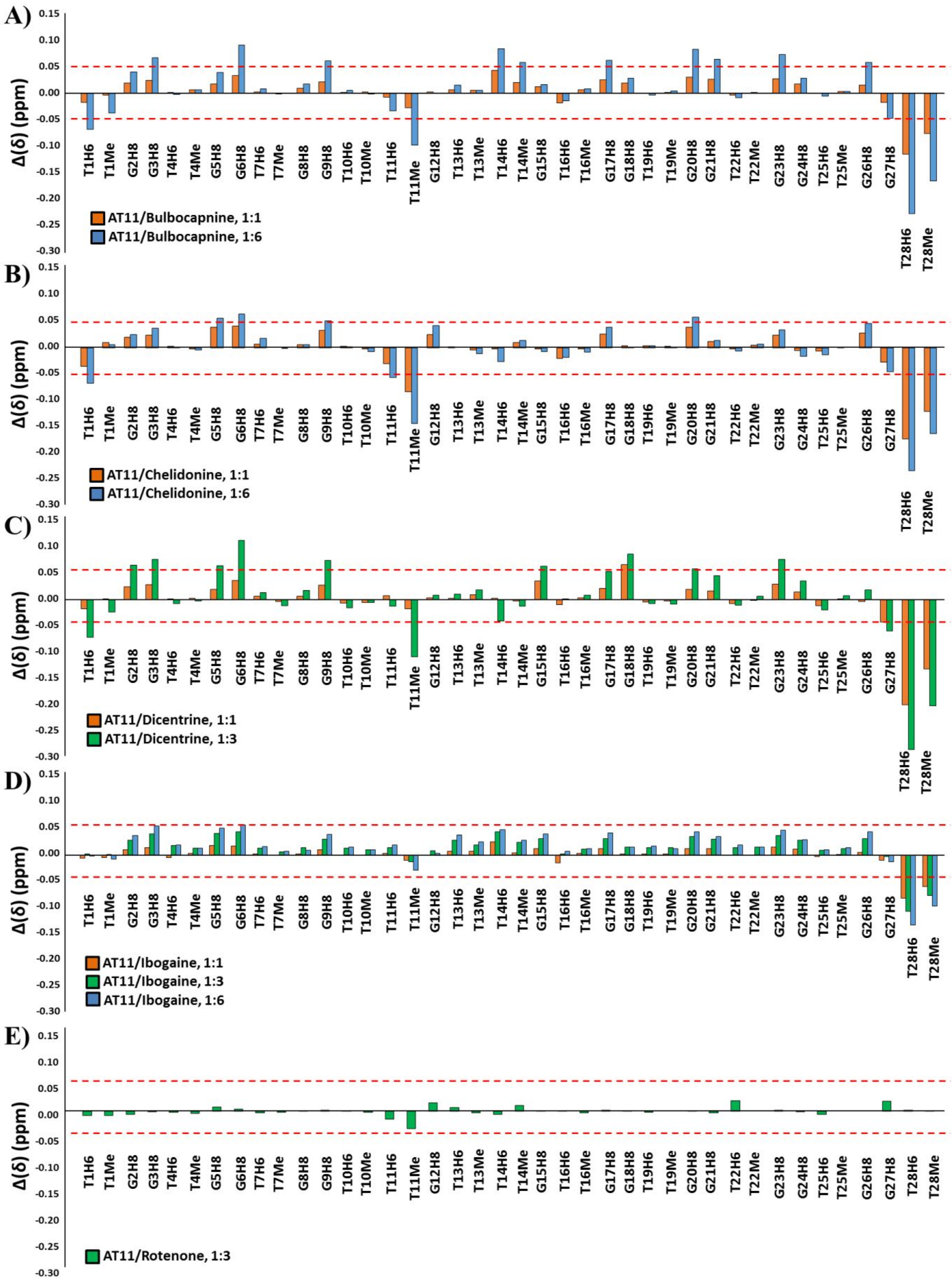

Figure S19. ^{1}H NMR chemical shift differences for aromatic and methyl protons of AT11 G-quadruplex in the presence of 1 (orange bars), 3 (green bars) or 6 (blue bars) molar equivalents of A) bulbocapnine, B) chelidonine, C) dicentrine, D) ibogaine and E) rotenone with respect to free AT11 G-quadruplex. The errors associated with chemical shift differences are within ±0.05 ppm. Threshold lines were drawn as dashed red lines.

Table S1. ^{1}H NMR chemical shifts of AT11 G-quadruplex in the absence and presence of small percentages of DMSO. The chemical shifts are given in ppm and correspond to spectra recorded in 90%/10% H_2O/D_2O at 0.2 mM DNA, 70 mM KCl, 20 mM potassium phosphate buffer (pH 7.0) and 25 °C. For a comparison, ^{1}H NMR chemical shifts of AT11 G-quadruplex taken from the previous work on AT11 are also reported.[1]

	**AT11 (No DMSO)**		**AT11 (previous report)**		**AT11 (0.5% DMSO)**		**AT11 (3% DMSO)**	
**Residue/ Proton**	H6/H8	H1/Me	H6/H8	H1/Me	H6/H8	H1/Me	H6/H8	H1/Me
**T1**	7.43	1.60	7.45	1.62	7.44	1.60	7.45	1.61
**G2**	8.10	11.52	8.12	11.56	8.09	11.52	8.09	11.52
**G3**	8.00	11.18	8.03	11.21	8.00	11.18	8.00	11.19
**T4**	7.91	1.98	7.94	2.00	7.91	1.97	7.91	1.97
**G5**	8.40	11.55	8.43	11.57	8.4	11.55	8.41	11.56
**G6**	8.04	11.29	8.07	11.31	8.04	11.29	8.04	11.29
**T7**	7.90	1.96	7.93	1.99	7.90	1.96	7.90	1.96
**G8**	8.29	11.45	8.33	11.47	8.30	11.44	8.30	11.44
**G9**	8.04	11.18	8.08	11.20	8.04	11.18	8.04	11.19
**T10**	7.82	1.96	7.84	1.99	7.82	1.96	7.82	1.96
**T11**	7.61	1.59	7.64	1.61	7.61	1.58	7.60	1.57
**G12**	8.02	11.35	8.05	11.37	8.02	11.35	8.02	11.34
**T13**	7.82	1.91	7.85	1.93	7.82	1.91	7.82	1.91
**T14**	7.57	1.77	7.60	1.82	7.56	1.77	7.56	1.78
**G15**	7.87	11.17	7.90	11.20	7.87	11.17	7.87	11.17
**T16**	7.54	1.91	7.59	1.94	7.55	1.91	7.57	1.91
**G17**	7.50	11.25	7.54	11.28	7.50	11.25	7.50	11.25
**G18**	7.86	11.30	7.89	11.33	7.86	11.30	7.87	11.30
**T19**	7.84	1.98	7.87	2.00	7.83	1.98	7.83	1.98
**G20**	7.71	11.17	7.74	11.19	7.71	11.17	7.71	11.17
**G21**	7.87	11.29	7.90	11.32	7.87	11.29	7.87	11.29
**T22**	7.79	1.98	7.83	2.01	7.79	1.98	7.79	1.98
**G23**	7.76	11.41	7.79	11.43	7.76	11.40	7.76	11.40
**G24**	7.89	11.17	7.91	11.19	7.89	11.17	7.89	11.17
**T25**	7.74	1.98	7.78	2.01	7.74	1.98	7.74	1.98
**G26**	7.61	11.11	7.65	11.13	7.62	11.11	7.62	11.11
**G27**	7.82	11.18	7.84	11.21	7.82	11.18	7.82	11.19
**T28**	7.35	1.61	7.38	1.64	7.36	1.62	7.39	1.64

[1] Do, N. Q.; Chung, W. J.; Truong, T. H. A.; Heddi, B.; Phan, A. T. G-Quadruplex Structure of an Anti-Proliferative DNA Sequence. *Nucleic Acids Res.* **2017**, *45* (12), 7487–7493. https://doi.org/10.1093/nar/gkx274.

Table S2. ^{1}H NMR chemical shifts of AT11 G-quadruplex in the presence of 1 molar equivalent of the indicated natural compound. The chemical shifts are given in ppm and correspond to spectra recorded in 90%/10% H_2O/D_2O at 0.2 mM DNA, 70 mM KCl, 20 mM potassium phosphate buffer (pH 7.0) and 25 °C.

	**AT11/Bulbocapnine 1:1**		**AT11/Chelidonine 1:1**		**AT11/Dicentrine 1:1**		**AT11/Ibogaine 1:1**	
**Residue/ Proton**	H6/H8	H1/Me	H6/H8	H1/Me	H6/H8	H1/Me	H6/H8	H1/Me
**T1**	7.45	1.60	7.47	1.59	7.45	1.60	7.44	1.61
**G2**	8.08	11.46	8.07	11.45	8.07	11.46	8.08	11.49
**G3**	7.97	11.15	7.97	11.13	7.97	11.15	7.98	11.16
**T4**	7.91	1.97	7.90	1.97	7.90	1.97	7.91	1.97
**G5**	8.39	11.49	8.37	11.55	8.38	11.50	8.39	11.52
**G6**	8.01	11.26	8.00	11.27	8.00	11.25	8.02	11.28
**T7**	7.89	1.96	7.89	1.95	7.89	1.96	7.89	1.96
**G8**	8.28	11.44	8.29	11.47	8.29	11.42	8.29	11.44
**G9**	8.02	11.16	8.01	11.19	8.01	11.16	8.03	11.17
**T10**	7.81	1.96	7.81	1.96	7.82	1.96	7.82	1.95
**T11**	7.62	1.62	7.64	1.67	7.60	1.60	7.60	1.59
**G12**	8.01	11.36	7.99	11.33	8.01	11.35	8.02	11.36
**T13**	7.82	1.91	7.82	1.92	7.82	1.90	7.81	1.90
**T14**	7.52	1.75	7.57	1.76	7.56	1.78	7.54	1.77
**G15**	7.86	11.10	7.87	11.13	7.83	11.14	7.86	11.16
**T16**	7.56	1.91	7.57	1.91	7.56	1.91	7.56	1.91
**G17**	7.48	11.21	7.47	11.21	7.48	11.15	7.49	11.23
**G18**	7.84	11.35	7.86	11.23	7.79	11.15	7.86	11.25
**T19**	7.84	1.98	7.83	1.97	7.84	1.98	7.83	1.97
**G20**	7.68	11.21	7.67	11.12	7.69	11.13	7.70	11.13
**G21**	7.85	11.21	7.86	11.21	7.85	11.21	7.86	11.26
**T22**	7.80	1.98	7.80	1.98	7.80	1.98	7.79	1.98
**G23**	7.73	11.36	7.74	11.40	7.73	11.31	7.75	11.37
**G24**	7.87	11.07	7.89	11.17	7.87	11.06	7.87	11.14
**T25**	7.74	1.98	7.74	1.98	7.75	1.98	7.74	1.98
**G26**	7.60	11.10	7.59	11.11	7.62	11.08	7.61	11.10
**G27**	7.83	11.21	7.84	11.18	7.86	11.04	7.82	11.15
**T28**	7.47	1.69	7.54	1.74	7.57	1.76	7.44	1.68

Table S3. ^{1}H NMR chemical shifts of AT11 G-quadruplex in the presence of 3 molar equivalents of the indicated natural compound. The chemical shifts are given in ppm and correspond to spectra recorded in 90%/10% H_2O/D_2O at 0.2 mM DNA, 70 mM KCl, 20 mM potassium phosphate buffer (pH 7.0) and 25 °C.

	**AT11/Dicentrine 1:3**		**AT11/Ibogaine 1:3**		**AT11/Rotenone 1:3**	
**Residue/ Proton**	H6/H8	H1/Me	H6/H8	H1/Me	H6/H8	H1/Me
**T1**	7.52	1.63	7.44	1.60	7.45	1.61
**G2**	8.02	11.30	8.06	11.45	8.10	11.51
**G3**	7.92	11.07	7.96	11.13	8.00	11.18
**T4**	7.91	1.97	7.89	1.96	7.91	1.98
**G5**	8.34	11.33	8.36	11.46	8.40	11.54
**G6**	7.92	11.16	7.99	11.26	8.04	11.29
**T7**	7.88	1.97	7.88	1.95	7.90	1.96
**G8**	8.28	11.39	8.28	11.43	8.30	11.47
**G9**	7.96	11.13	8.01	11.16	8.04	11.17
**T10**	7.83	1.96	7.80	1.94	7.82	1.96
**T11**	7.62	1.69	7.58	1.59	7.62	1.61
**G12**	8.01	11.37	8.01	11.35	8.00	11.33
**T13**	7.81	1.89	7.79	1.89	7.82	1.91
**T14**	7.61	1.79	7.52	1.75	7.57	1.76
**G15**	7.81	11.08	7.84	11.13	7.87	11.17
**T16**	7.55	1.90	7.54	1.90	7.55	1.91
**G17**	7.45	11.07	7.47	11.21	7.50	11.25
**G18**	7.77	11.06	7.85	11.21	7.87	11.30
**T19**	7.84	1.98	7.82	1.96	7.84	1.98
**G20**	7.65	11.07	7.67	11.12	7.71	11.17
**G21**	7.82	11.13	7.84	11.20	7.87	11.29
**T22**	7.80	1.97	7.78	1.97	7.77	1.98
**G23**	7.68	11.24	7.72	11.35	7.76	11.40
**G24**	7.85	10.97	7.85	11.10	7.89	11.17
**T25**	7.76	1.97	7.73	1.97	7.74	1.98
**G26**	7.60	11.05	7.59	11.09	7.62	11.12
**G27**	7.88	10.95	7.82	11.12	7.80	11.18
**T28**	7.67	1.84	7.48	1.71	7.37	1.63

Table S4. ^{1}H NMR chemical shifts of AT11 G-quadruplex in the presence of 6 molar equivalents of the indicated natural compound. The chemical shifts are given in ppm and correspond to spectra recorded in 90%/10% H_2O/D_2O at 0.2 mM DNA, 70 mM KCl, 20 mM potassium phosphate buffer (pH 7.0) and 25 °C.

	**AT11/Bulbocapnine 1:6**		**AT11/Chelidonine 1:6**		**AT11/Ibogaine 1:6**	
**Residue/ Proton**	H6/H8	H1/Me	H6/H8	H1/Me	H6/H8	H1/Me
**T1**	7.50	1.64	7.52	1.60	7.45	1.61
**G2**	8.05	11.40	8.07	11.40	8.05	11.41
**G3**	7.93	11.10	7.96	11.20	7.94	11.11
**T4**	7.91	1.97	7.90	1.98	7.88	1.96
**G5**	8.36	11.36	8.35	11.47	8.36	11.43
**G6**	7.95	11.20	7.97	11.25	7.98	11.26
**T7**	7.89	1.96	7.88	1.96	7.88	1.95
**G8**	8.28	11.43	8.30	11.49	8.29	11.43
**G9**	7.98	11.16	7.99	11.19	8.00	11.17
**T10**	7.81	1.96	7.82	1.96	7.80	1.94
**T11**	7.65	1.70	7.66	1.72	7.58	1.60
**G12**	8.02	11.39	7.97	11.34	8.01	11.37
**T13**	7.81	1.91	7.82	1.92	7.78	1.88
**T14**	7.48	1.71	7.59	1.76	7.51	1.75
**G15**	7.86	11.12	7.88	11.12	7.83	11.12
**T16**	7.56	1.91	7.57	1.92	7.55	1.90
**G17**	7.44	11.14	7.46	11.21	7.46	11.19
**G18**	7.83	11.07	7.87	11.18	7.85	11.19
**T19**	7.84	1.97	7.83	1.97	7.81	1.96
**G20**	7.62	11.09	7.65	11.11	7.67	11.10
**G21**	7.81	11.21	7.85	11.18	7.83	11.18
**T22**	7.80	1.98	7.80	1.97	7.77	1.97
**G23**	7.69	11.31	7.73	11.39	7.71	11.34
**G24**	7.86	11.11	7.90	11.12	7.85	11.10
**T25**	7.75	1.98	7.75	1.98	7.73	1.97
**G26**	7.55	10.99	7.57	11.11	7.58	11.09
**G27**	7.87	11.01	7.86	11.08	7.83	11.11
**T28**	7.59	1.79	7.63	1.81	7.52	1.74

Table S5. Intermolecular NOE cross-peaks between the indicated natural compound and AT11 G-quadruplex observed at the reported AT11/ligand ratios, NOESY mixing times (τ_m) and temperatures.

**Chemical structure**		**AT11/ligand ratio, τ_m, t**		
	Chelidonine protons	**1:1, 200 ms, 25 °C**	**1:6, 200 ms, 25 °C**	
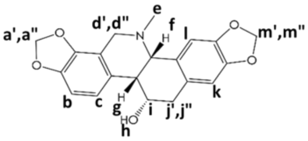	a′,a″	T1H6, T1Me, G2H8, G8H8, G12H4′	T1H6, T1Me, G2H8, G8H8, G12H4′	
	m′,m″	T1H6, T1Me, G2H8, G8H8, G12H4′	T1H6, T1Me, G2H8, G8H8, G12H4′	

	Dicentrine protons	**1:1, 200 ms, 25 °C**	**1:1, 400 ms, 25 °C**	**1:3, 400 ms, 25 °C**
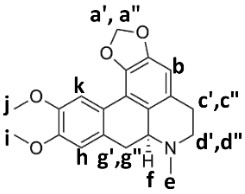	i	G18H8, G18H1′, G18H2′, G18H2″, G21H8, G24H2″, G27H8	G17H2″, G18H8, G18H1′, G18H2′, G18H2″, G21H8, G24H8, G24H2″, G27H8, G27H2′, G27H2″	G2H8, G3H8, G5H1, G5H8, G8H1, G9H8, G12H8, G18H8, G18H2′, G18H2″, G18H8, G21H8, G24H8, G24H2″, G27H8
	j	G18H8, G18H2′, G18H2″, G21H8, G24H8, G27H8	G18H8, G18H2′, G18H2″, G21H8, G24H8, G27H8, G27H2′, G27H2″	G6H8, G8H8, G18H8, G18H2′, G18H2″, G21H8, G24H8, G27H8

	Ibogaine protons	**1:1, 400 ms, 25 °C**	**1:3, 400 ms, 25 °C**	**1:6, 400 ms, 25 °C**
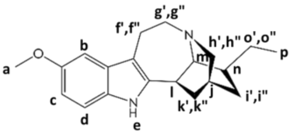	a	G21H8	G21H8	G15H8, G21H8, G24H8, G27H8
	c	-	G24H8	G24H8, G27H8
	m	G21H1	G24H1	G18H1, G20H1, G21H1, G27H1
	o	G21H1, G27H1	G17H1, G18H1, G21H1	G17H1, G18H1, G21H1, G26H1

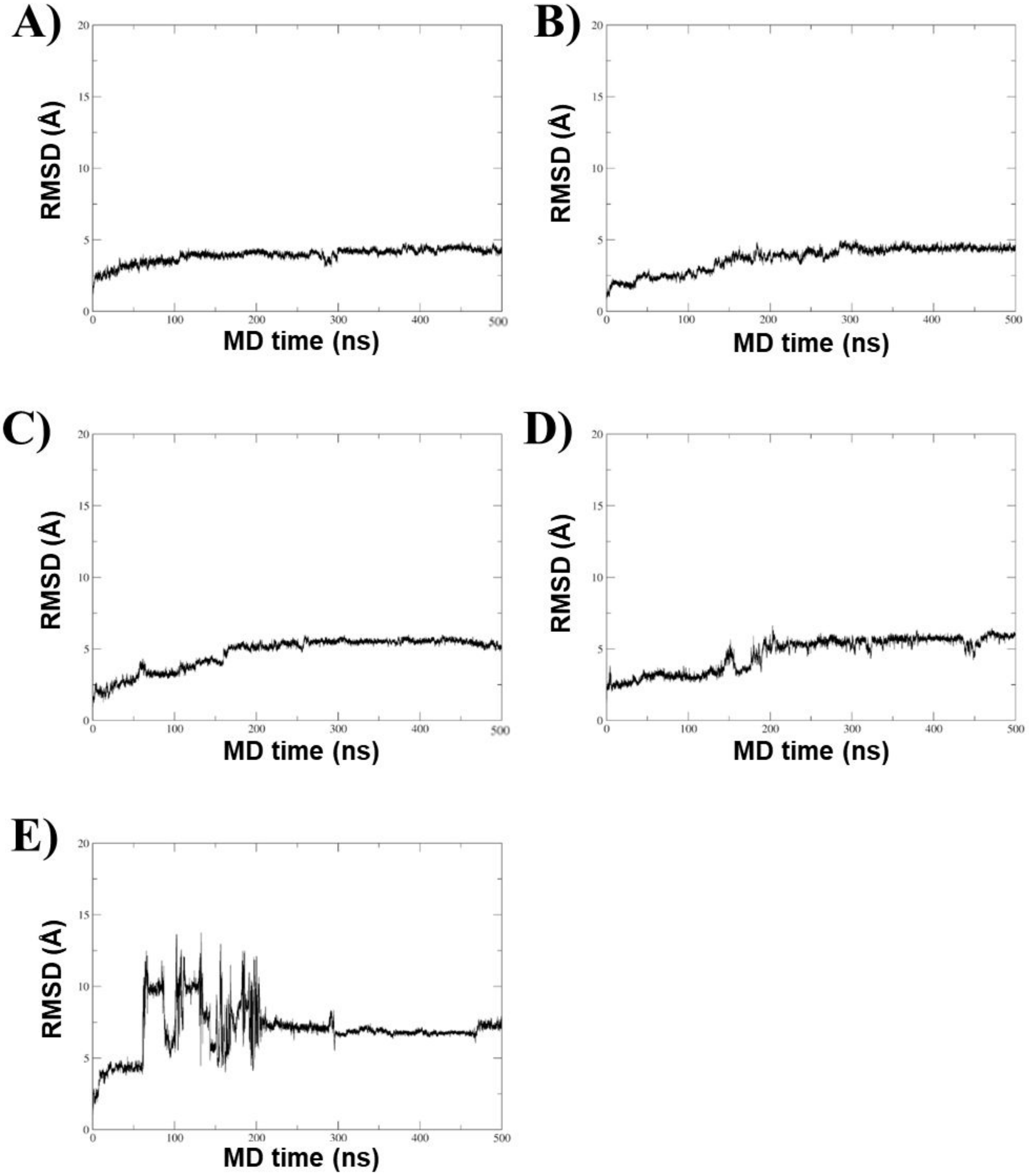

Figure S20. All-atom RMSD values of A) AT11/bulbocapnine, B) AT11/chelidonine, C) AT11/dicentrine, D) AT11/ibogaine and E) AT11/rotenone complexes along MD simulation time. RMSD values were calculated taking as reference the first frame of each MD simulation. Solvent molecules were not included in RMSD values calculation.

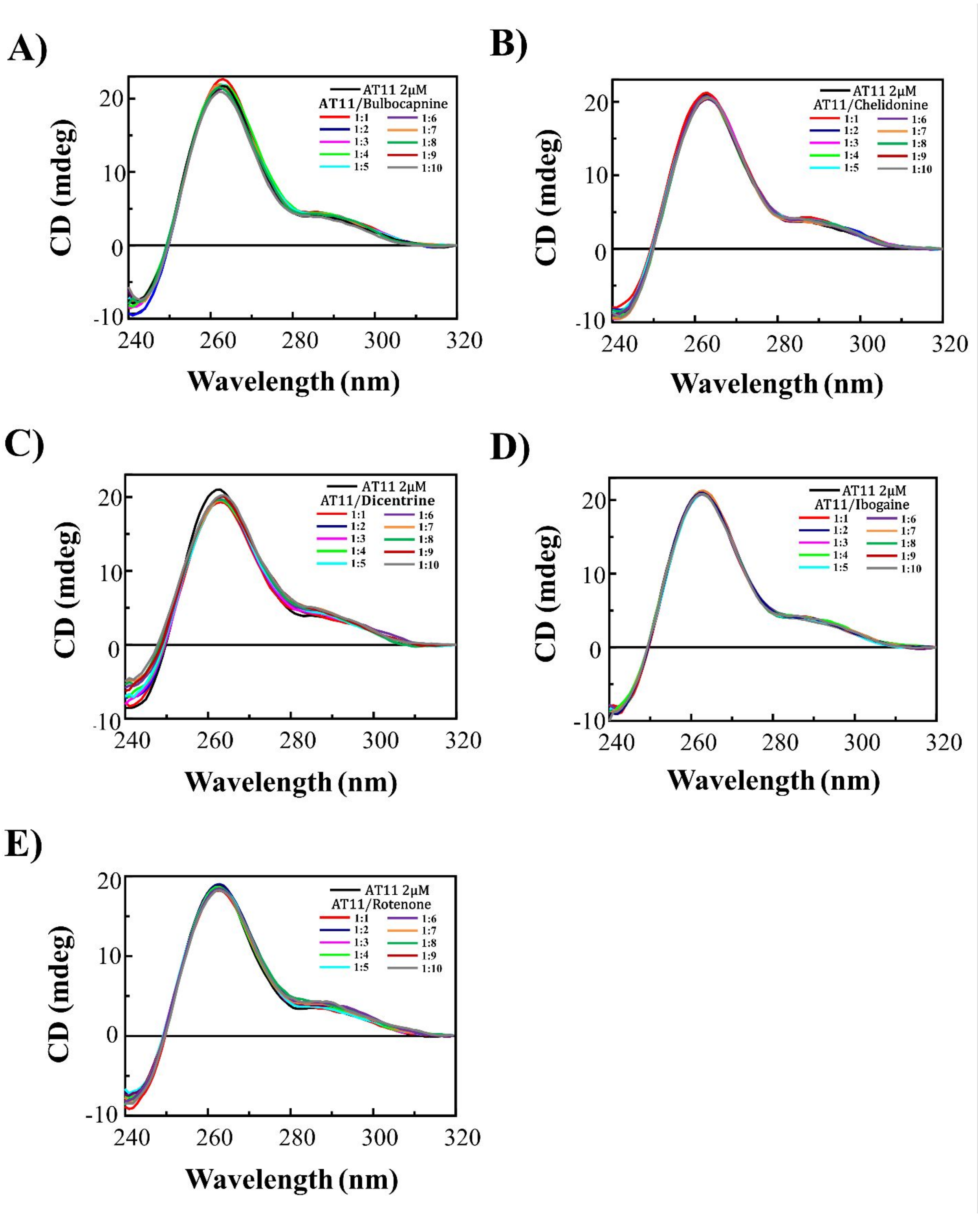

Figure S21. CD spectra of 2 μM solutions of AT11 G-quadruplex in 70 mM KCl, 20 mM potassium phosphate buffer (pH 7.0) in the presence of increasing amounts (up to 10 equivalents) of A) bulbocapnine, B) chelidonine, C) dicentrine, D) ibogaine and E) rotenone (the contribution to the CD spectra given by the individual ligands – see Figure S22 - was here subtracted).

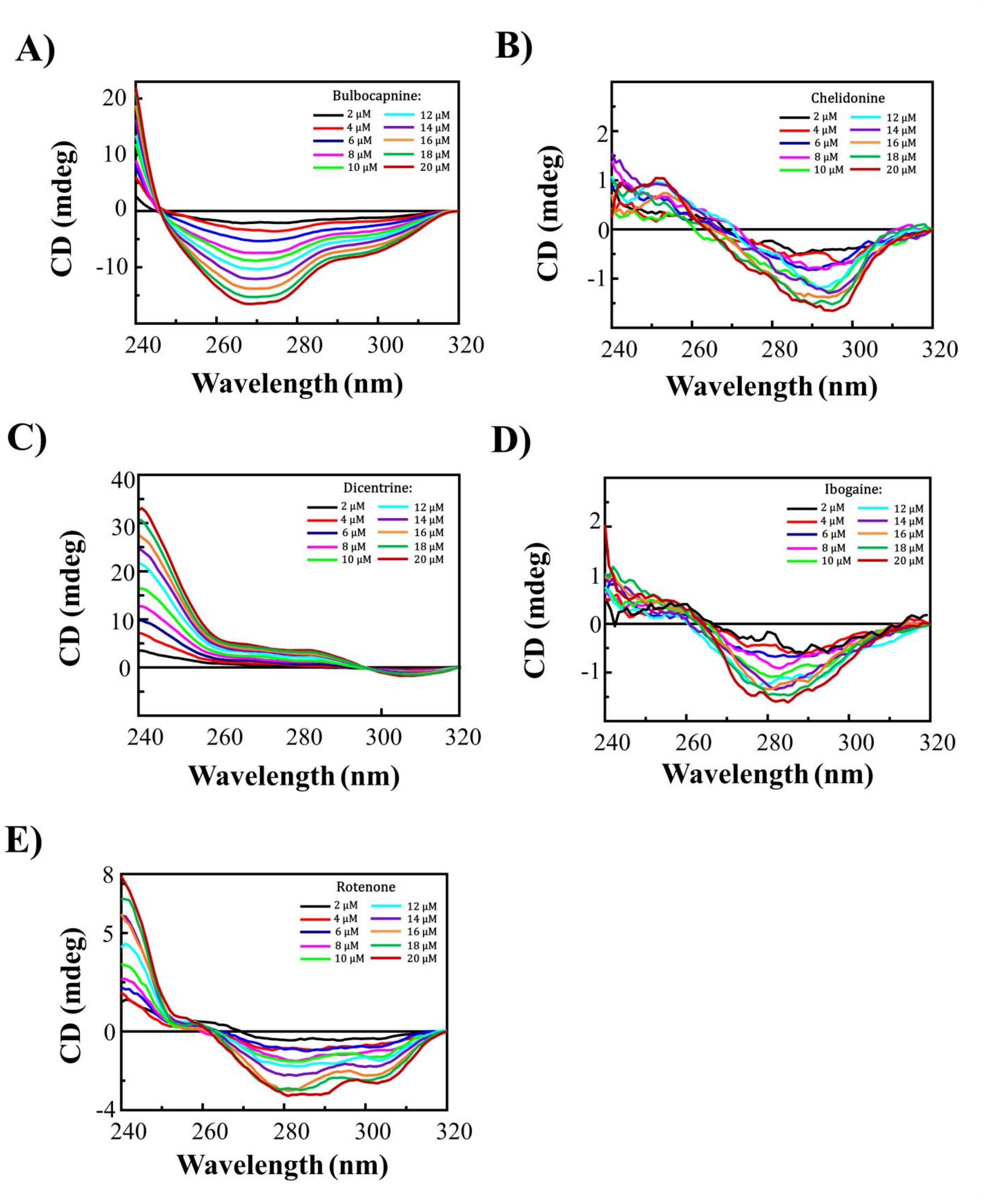

Figure S22. CD spectra of solutions (from 2 to 20 μM) of A) bulbocapnine, B) chelidonine, C) dicentrine, D) ibogaine and E) rotenone in 70 mM KCl, 20 mM potassium phosphate buffer (pH 7.0).

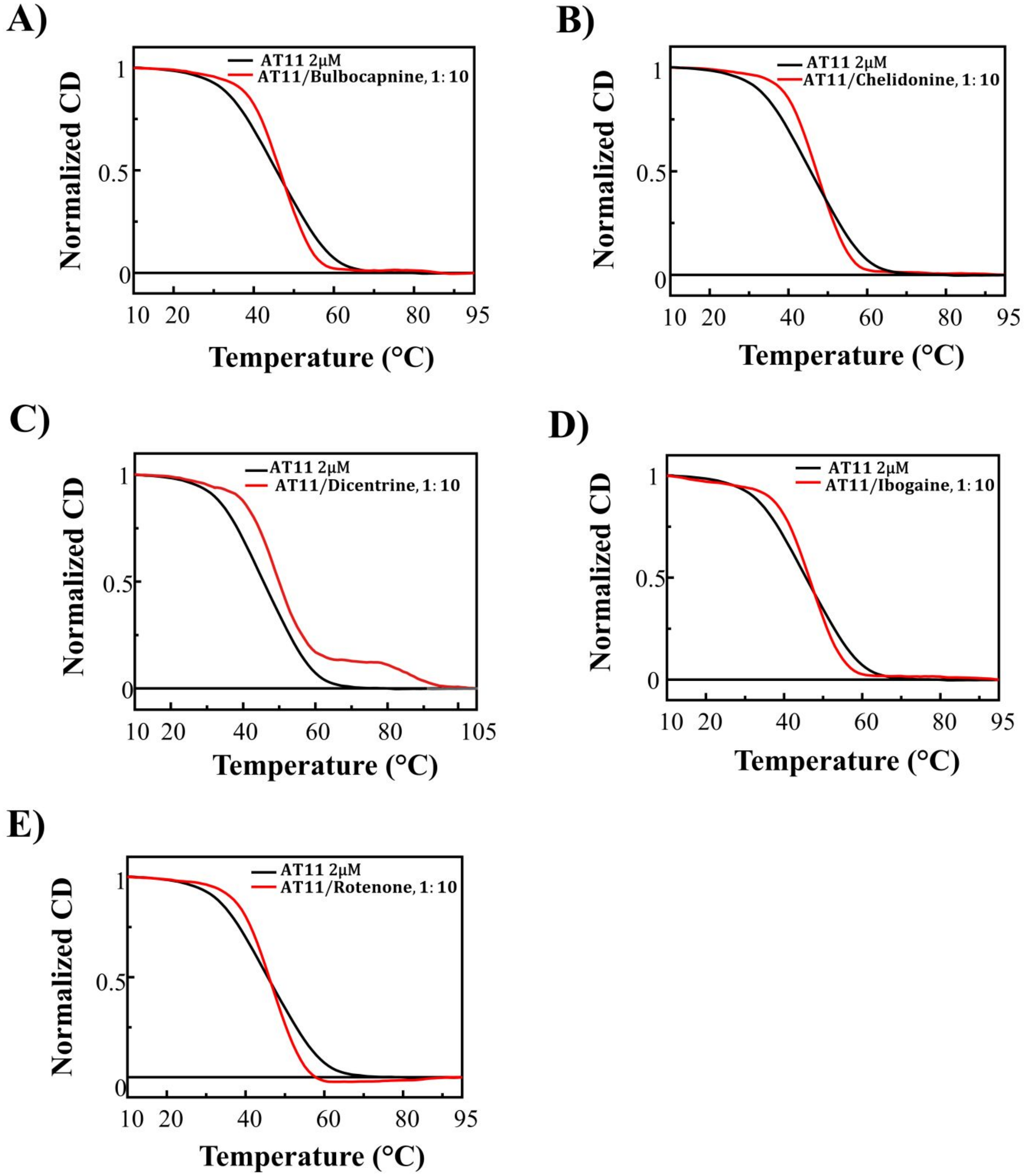

Figure S23. Normalized CD melting curves of AT11 G-quadruplex in the absence and presence of 10 molar equivalents of A) bulbocapnine, B) chelidonine, C) dicentrine, D) ibogaine and E) rotenone in 70 mM KCl, 20 mM potassium phosphate buffer (pH 7.0), recorded at 262 nm.

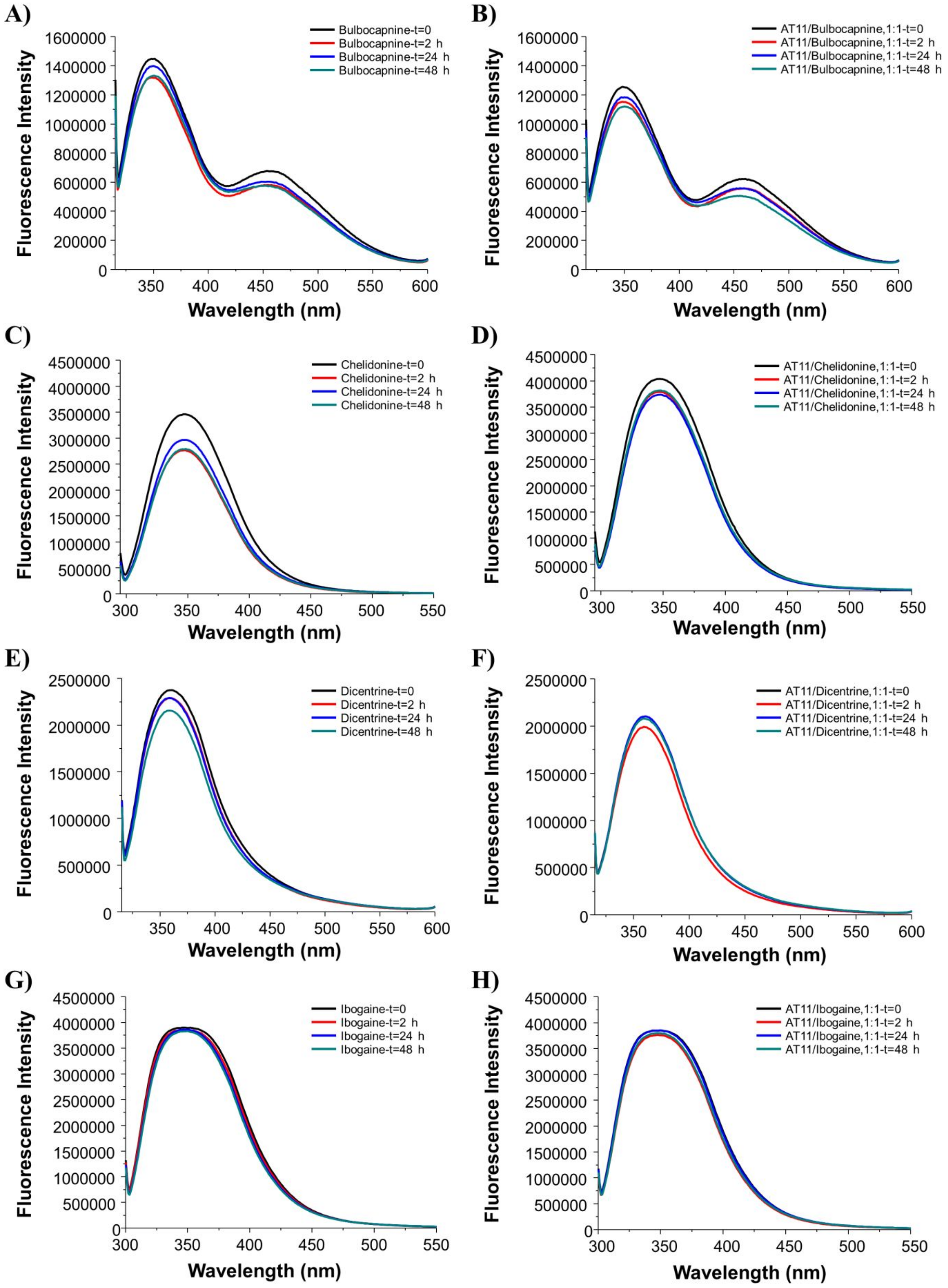

Figure S24. Fluorescence emission spectra of solutions of free A) bulbocapnine, C) chelidonine, E) dicentrine and G) ibogaine and of 1:1 B) AT11/bulbocapnine, D) AT11/chelidonine, F) AT11/dicentrine and H) AT11/ibogaine complexes, at 2 μM ligand and DNA concentration, in 70 mM KCl, 20 mM potassium phosphate buffer (pH 7.0) with 10% FBS, after the indicated time of incubation at 37 °C.

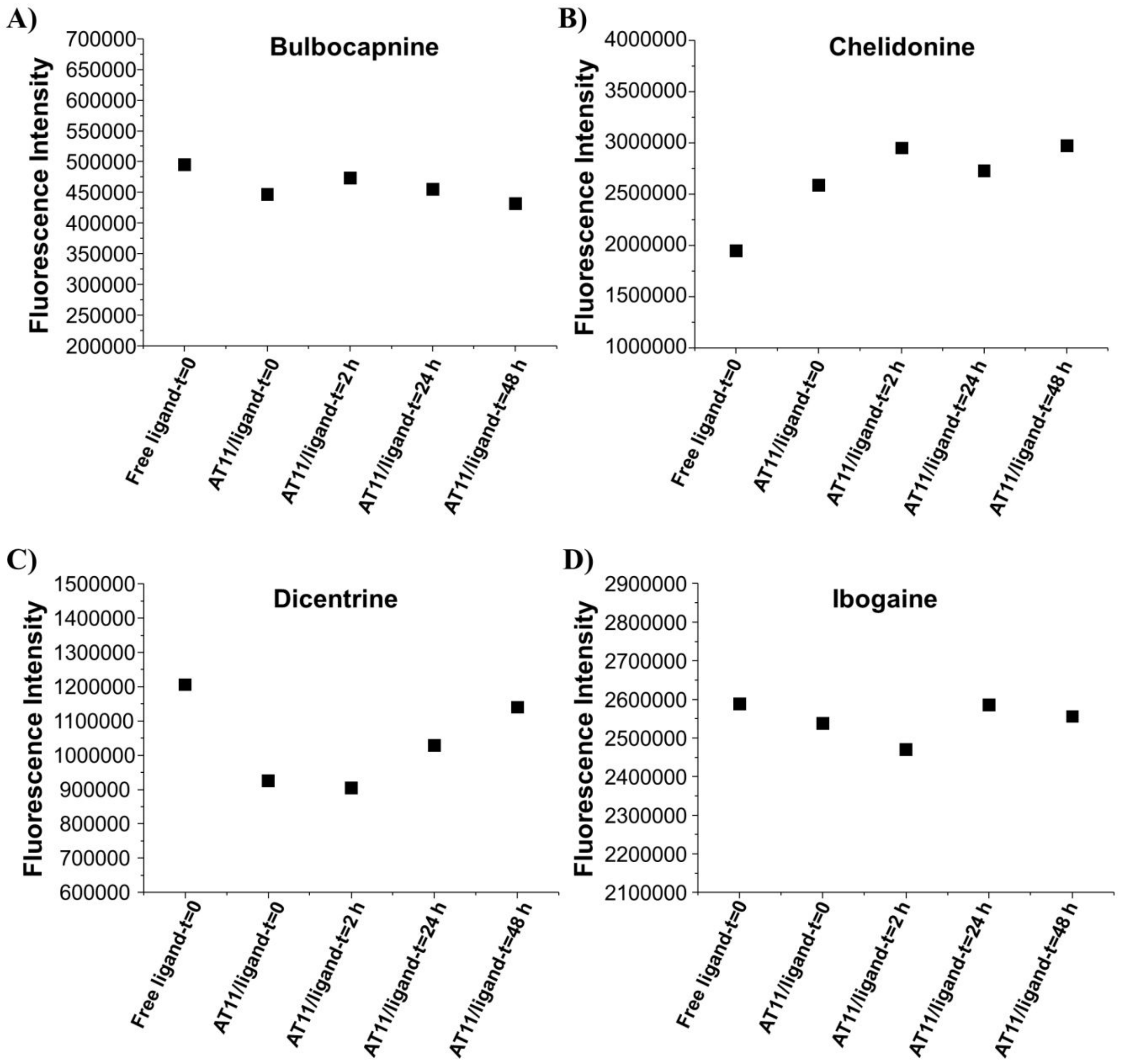

Figure S25. Fluorescence intensity for free ligands and 1:1 AT11/ligand complexes for A) bulbocapnine, B) chelidonine, C) dicentrine and D) ibogaine, obtained for solutions at 2 μM ligand and DNA concentration, in 70 mM KCl, 20 mM potassium phosphate buffer (pH 7.0) with 10% FBS, after the indicated time of incubation at 37 °C.

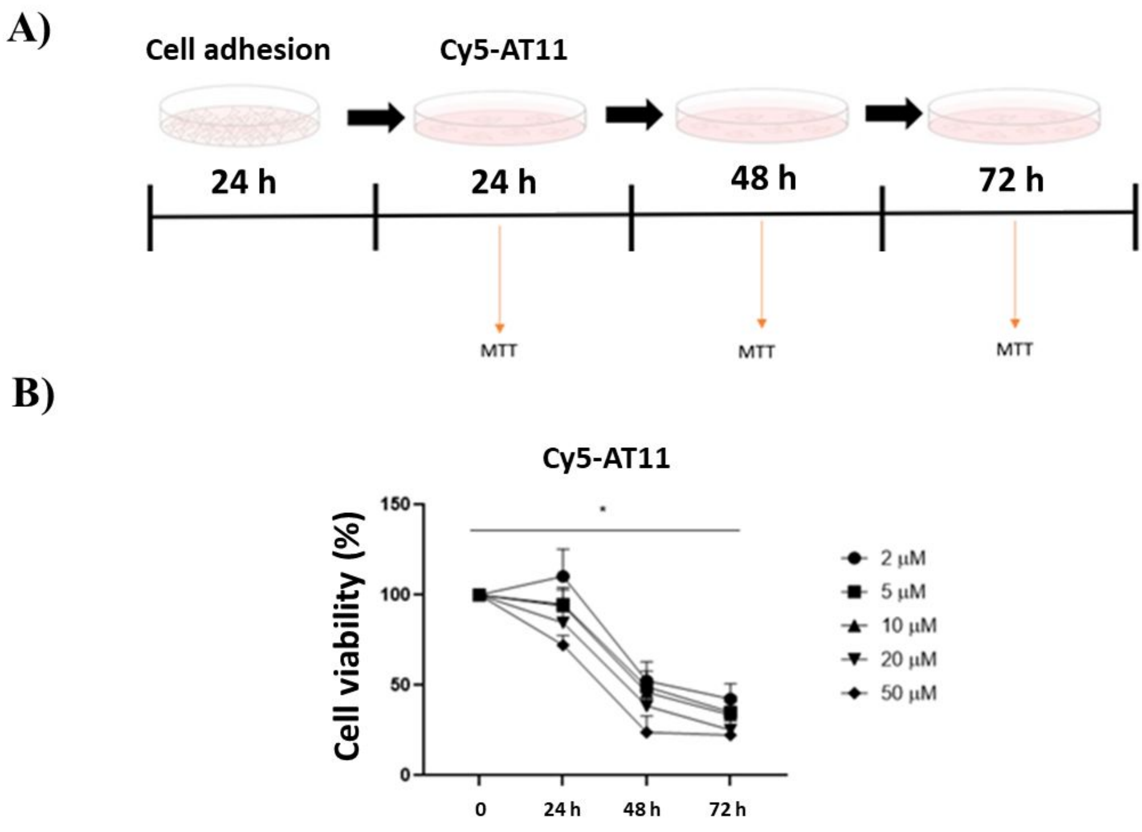

Figure S26. A) Schematic representation of the experimental protocol adopted for the cell viability assay of Cy5-AT11. B) AGS cells were treated for 24, 48 and 72 h at five different concentrations (2, 5, 10, 20 and 50 μM) with Cy5-AT11 and cell viability was measured by MTT assay. Data are expressed as mean ± SD (standard deviation), n = 3. The significance was determined using 2-way ANOVA *($p < 0.05$).

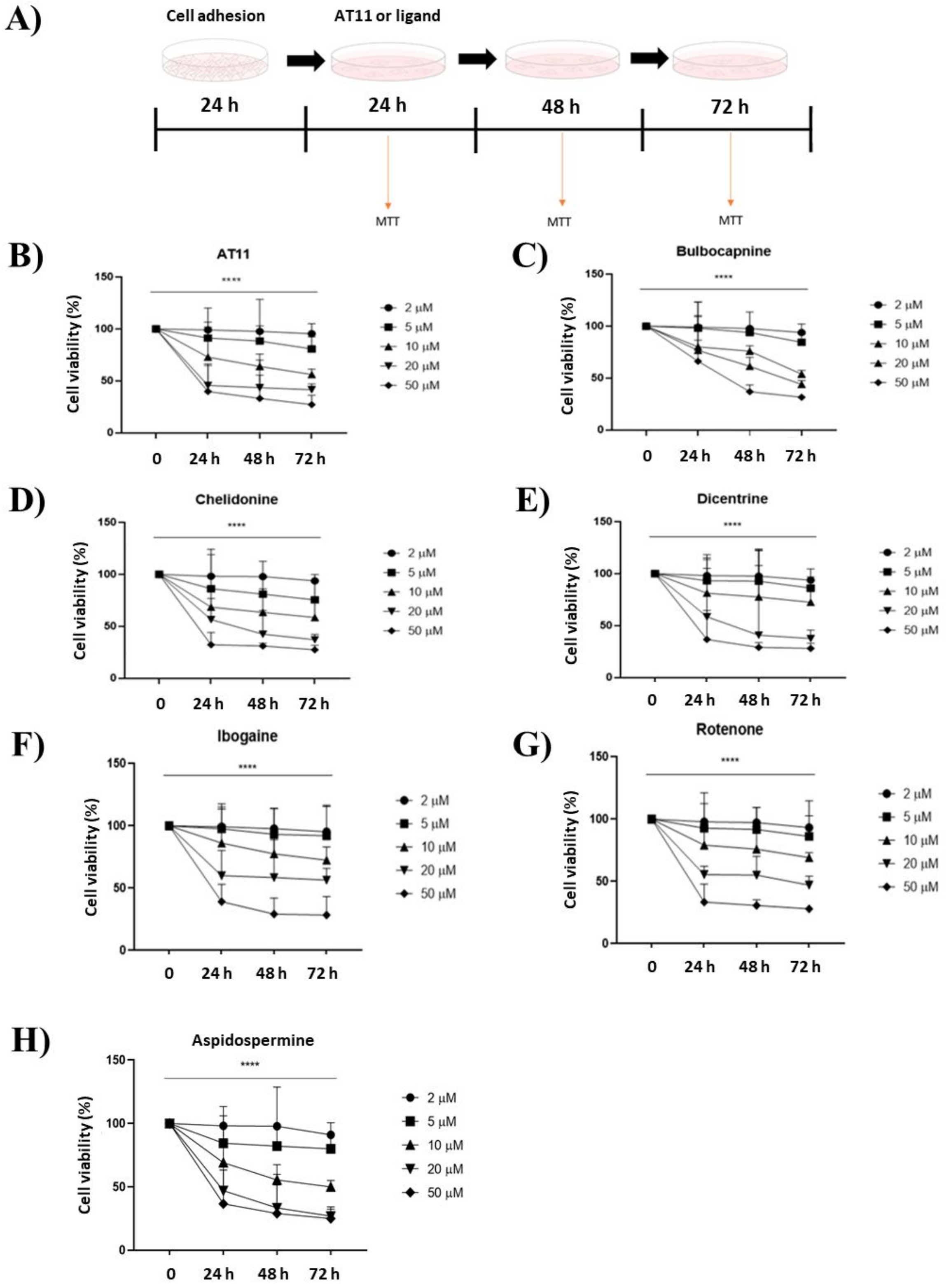

Figure S27. A) Schematic representation of the experimental protocol adopted for the cell viability assay of free AT11 or free ligands. AGS cells were treated for 24, 48 and 72 h with B) AT11, C) bulbocapnine, D) chelidonine, E) dicentrine, F) ibogaine, G) rotenone and H) aspidospermine at five different concentrations (2, 5, 10, 20 and 50 μM) and cell viability was measured by MTT assay. Data are expressed as mean ± SD (standard deviation), n = 3. The significance was determined using 2-way ANOVA ****($p < 0.001$).

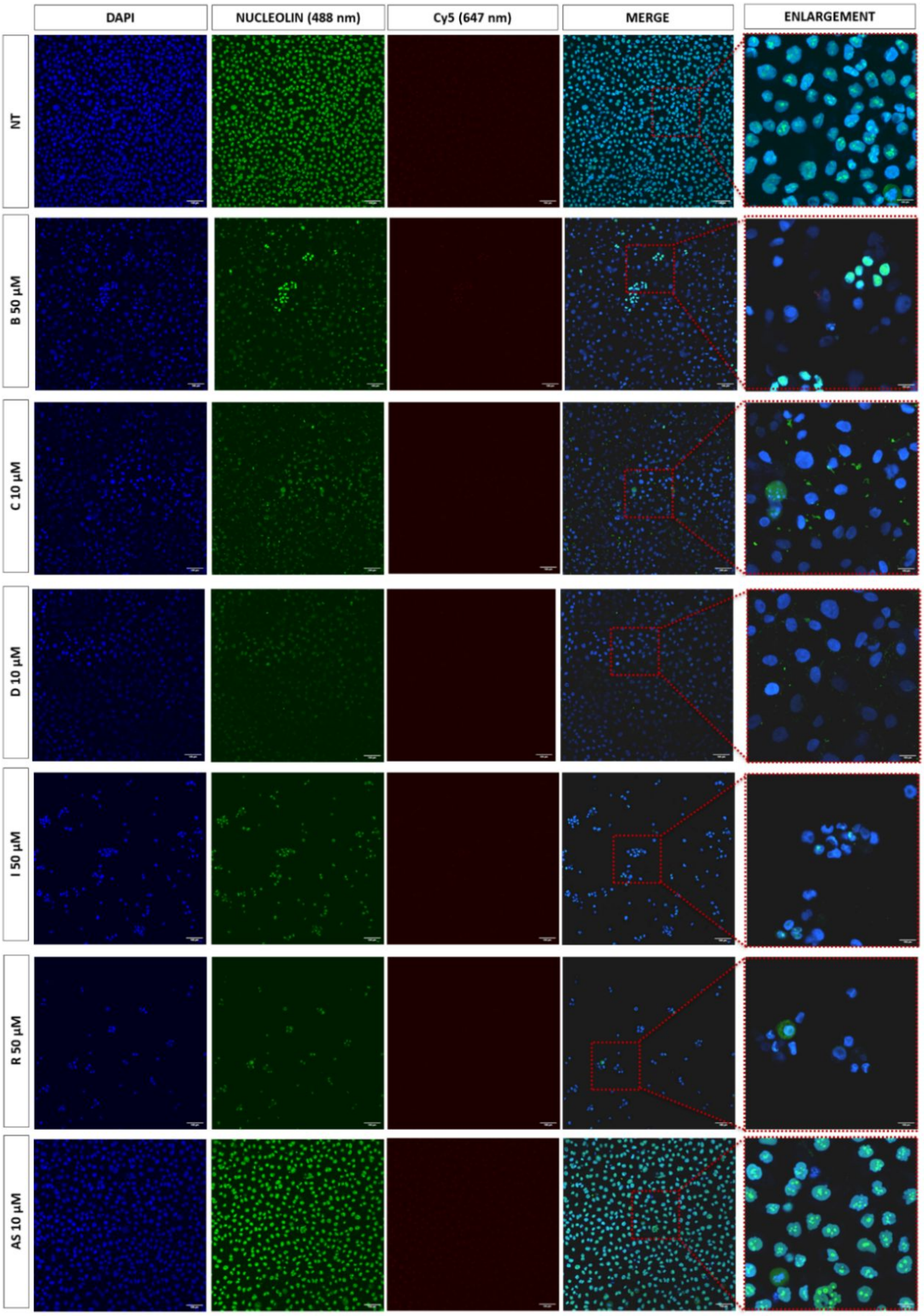

Figure S28. Immunofluorescence images of AGS cells non-treated (NT) or treated for 72 h with the indicated concentrations of free bulbocapnine (B), chelidonine (C), dicentrine (D), ibogaine (I), rotenone (R) and aspidospermine (AS). Nucleolin was stained with Alexa Fluor 488 anti-rabbit secondary antibody (green), while nuclei were stained with DAPI (blue). The acquisitions with the use of Cy5 channel (647 nm) are also shown. Scale bar: 100 µm. Acquisition: 20X (without zoom) or 20X (with 4X zoom) by Olympus IX83 confocal microscope.

Table S6. Minimum (Min) and maximum (Max) intensity values measured by using ImageJ software in AGS cells non-treated (NT) or treated with the indicated concentrations of free Cy5-AT11, free natural compounds or their complexes. The area indicates the surface of the selected region of interest (ROI) expressed in pixel^2. The minimum refers to the lowest pixel intensity value within the selected region, while the maximum represents the highest pixel intensity value within the selected region. Data are expressed as relative positivity percentages, normalized to the maximum intensity value observed in the non-treated sample (set as 100%).

	**Area**	**Min**	**Max**	**% of positivity**
**NT**	1048576	9	154	100.0
**Cy5-AT11 (50 µM)**	1048576	9	165	107.1
**Bulbocapnine (50 µM)**	1048576	9	135	87.7
**Chelidonine (10 µM)**	1048576	9	147	95.5
**Dicentrine (10 µM)**	1048576	9	114	74.0
**Ibogaine (50 µM)**	1048576	9	151	98.1
**Rotenone (50 µM)**	1048576	8	136	88.3
**Aspidospermine (10 µM)**	1048576	9	142	92.2
**Cy5-AT11 (50 µM) + Bulbocapnine (50 µM)**	1048576	9	175	113.6
**Cy5-AT11 (10 µM) + Chelidonine (10 µM)**	1048576	9	175	113.6
**Cy5-AT11 (10 µM) + Dicentrine (10 µM)**	1048576	9	166	107.8
**Cy5-AT11 (50 µM) + Ibogaine (50 µM)**	1048576	9	198	128.6
**Cy5-AT11 (50 µM) + Rotenone (50 µM)**	1048576	9	199	129.2
**Cy5-AT11 (10 µM) + Aspidospermine (10 µM)**	1048576	8	152	98.7

Table S7. IC_{50} values for free AT11 G-quadruplex and free ligands in AGS cells treated for 72 h.

	**IC_{50} (µM)**
**AT11**	8.8
**Bulbocapnine**	39.5
**Chelidonine**	9.2
**Dicentrine**	15.9
**Ibogaine**	48.5
**Rotenone**	27.0
**Aspidospermine**	11.5

Table S8. Analysis of synergistic effects by the Bliss independence model for the AT11/natural compounds complexes compared to the free AT11 G-quadruplex and free ligands in AGS cells treated for 72 h.

	E_{AB}	E_{Bliss}
**AT11 (50 µM) + Bulbocapnine (50 µM)**	0.65	0.59
**AT11 (10 µM) + Chelidonine (10 µM)**	0.72	0.68
**AT11 (10 µM) + Dicentrine (10 µM)**	0.89	0.83
**AT11 (50 µM) + Ibogaine (50 µM)**	0.88	0.61
**AT11 (50 µM) + Rotenone (50 µM)**	0.87	0.51
**AT11 (10 µM) + Aspidospermine (10 µM)**	0.60	0.60

Table S9. Number of cells counted as DAPI-positive cells and number of Cy5-AT11-positive cells measured by using ImageJ software for AGS cells non-treated (NT) or treated with the indicated concentrations of free Cy5-AT11 or its complexes with the natural compounds. The area indicates the surface of the selected region of interest (ROI) expressed in pixel². Data are expressed as relative positivity percentages: (Number of Cy5-AT11 positive cells/Number of cells counted in the selected area)*100.

	**Area**	**Number of cells**	**Number of Cy5-AT11 positive cells**	**% of positivity**
**NT**	1048576	400	0	0.0
**Cy5-AT11 (50 µM)**	1048576	200	67	33.5
**Cy5-AT11 (50 µM) + Bulbocapnine (50 µM)**	1048576	125	80	64.0
**Cy5-AT11 (10 µM) + Chelidonine (10 µM)**	1048576	130	81	62.3
**Cy5-AT11 (10 µM) + Dicentrine (10 µM)**	1048576	125	73	58.4
**Cy5-AT11 (50 µM) + Ibogaine (50 µM)**	1048576	135	85	63.0
**Cy5-AT11 (50 µM) + Rotenone (50 µM)**	1048576	128	83	64.8
**Cy5-AT11 (10 µM) + Aspidospermine (10 µM)**	1048576	260	37	14.2

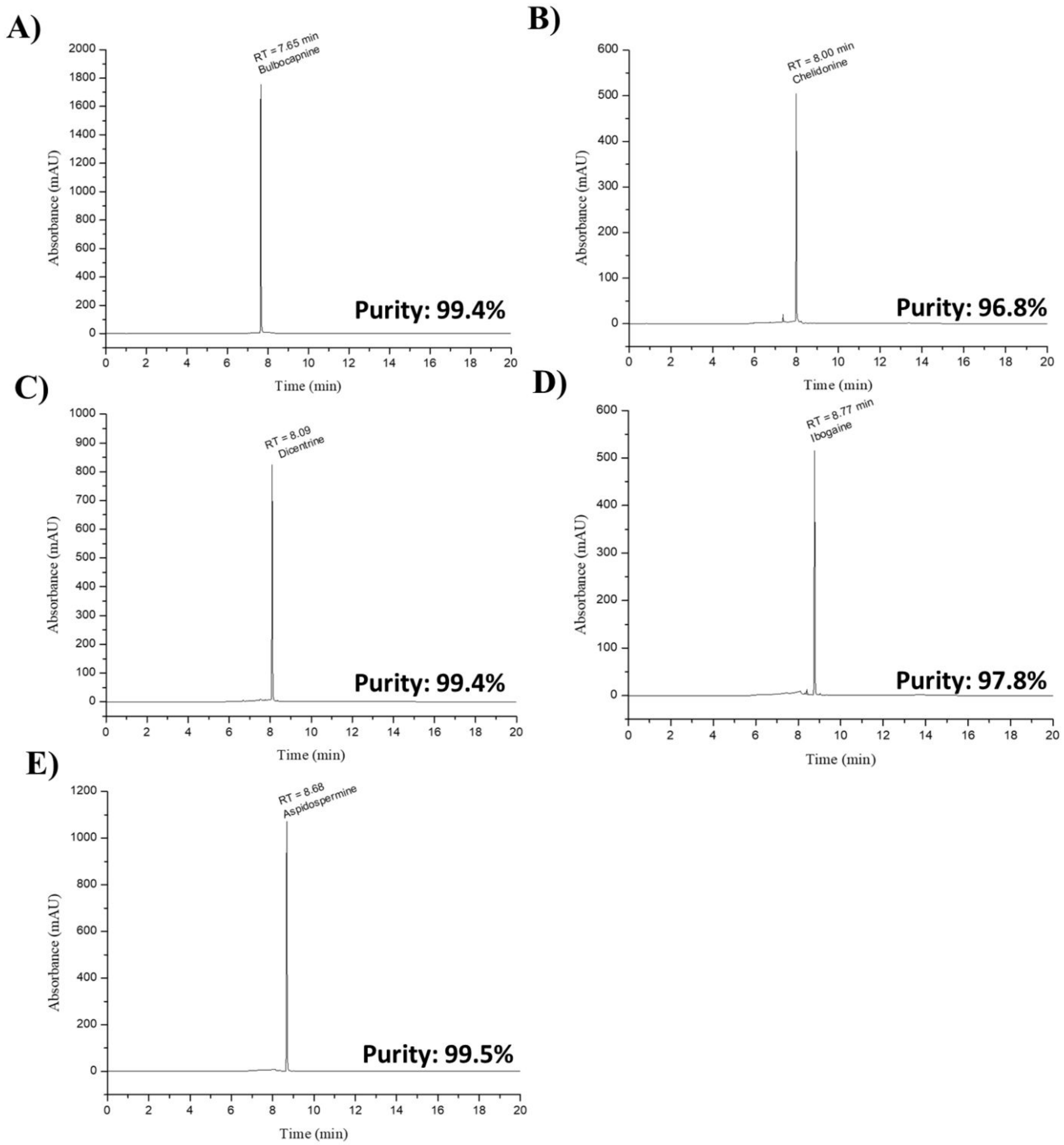

Figure S29. HPLC traces for A) bulbocapnine, B) chelidonine, C) dicentrine, D) ibogaine and E) aspidospermine. Instrument: Ultimate 3000 ultra-high-performance liquid chromatograph (Thermo Fisher Scientific, Waltham, MA, USA). Software: Chromeleon 7.2 Chromatography Data System software (Thermo Fisher Scientific, 1.0.5. v). Column: Ascentis® Express 90Å C18 (100 × 3 mm, l × i.d., 2 μm) (Sigma-Aldrich, St. Louis, MO, USA). Mobile phase: (*A*) 20 mM ammonium acetate and (*B*) acetonitrile. Gradient: 5% *B* (0 min), 5% *B* (3 min), 100% *B* (6 min), 100% *B* (11 min), 5% *B* (12 min), and 5% *B* (20 min). Flow rate: 0.5 mL/min. Column temperature: 40 °C. Injection volume: 5 μL. Detection wavelength: 260 nm.
